# Exploring the role of polymorphic interspecies structural variants in reproductive isolation and adaptive divergence in *Eucalyptus*

**DOI:** 10.1093/gigascience/giae029

**Published:** 2024-06-13

**Authors:** Scott Ferguson, Ashley Jones, Kevin Murray, Rose L Andrew, Benjamin Schwessinger, Helen Bothwell, Justin Borevitz

**Affiliations:** Research School of Biology, Australian National University, Canberra, Australian Capital Territory, 2600 Australia; Research School of Biology, Australian National University, Canberra, Australian Capital Territory, 2600 Australia; Research School of Biology, Australian National University, Canberra, Australian Capital Territory, 2600 Australia; Department of Molecular Biology, Max Planck Institute for Biology Tübingen, Tübingen, 72076 Germany; Botany & N.C.W. Beadle Herbarium, School of Environmental and Rural Science, University of New England, Armidale, NSW 2351, Australia; Research School of Biology, Australian National University, Canberra, Australian Capital Territory, 2600 Australia; Research School of Biology, Australian National University, Canberra, Australian Capital Territory, 2600 Australia; Warnell School of Forestry & Natural Resources, University of Georgia, Athens 30602 GA, United States; Research School of Biology, Australian National University, Canberra, Australian Capital Territory, 2600 Australia

**Keywords:** *Eucalyptus*, structural variations, adaptive evolution, genome divergence, comparative genomics

## Abstract

Structural variations (SVs) play a significant role in speciation and adaptation in many species, yet few studies have explored the prevalence and impact of different categories of SVs. We conducted a comparative analysis of long-read assembled reference genomes of closely related *Eucalyptus* species to identify candidate SVs potentially influencing speciation and adaptation. Interspecies SVs can be either fixed differences or polymorphic in one or both species. To describe SV patterns, we employed short-read whole-genome sequencing on over 600 individuals of *Eucalyptus melliodora* and *Eucalyptus sideroxylon*, along with recent high-quality genome assemblies. We aligned reads and genotyped interspecies SVs predicted between species reference genomes. Our results revealed that 49,756 of 58,025 and 39,536 of 47,064 interspecies SVs could be typed with short reads in *E. melliodora* and *E. sideroxylon*, respectively. Focusing on inversions and translocations, symmetric SVs that are readily genotyped within both populations, 24 were found to be structural divergences, 2,623 structural polymorphisms, and 928 shared structural polymorphisms. We assessed the functional significance of fixed interspecies SVs by examining differences in estimated recombination rates and genetic differentiation between species, revealing a complex history of natural selection. Shared structural polymorphisms displayed enrichment of potentially adaptive genes. Understanding how different classes of genetic mutations contribute to genetic diversity and reproductive barriers is essential for understanding how organisms enhance fitness, adapt to changing environments, and diversify. Our findings reveal the prevalence of interspecies SVs and elucidate their role in genetic differentiation, adaptive evolution, and species divergence within and between populations.

## Introduction

Structural mutations that alter stretches of DNA greater than 50 bp in length have the potential to drastically change phenotypes [1–3] and contribute to population divergence and speciation [4, 5]. Typically termed chromosomal rearrangements or structural variations (SVs), these large mutations include inversions, translocations, duplications, insertions, and deletions [6]. Until recently, however, technological constraints—namely, sequencing read lengths—have inhibited their discovery [7], and their role in population evolutionary processes remains poorly understood [8]. Using third-generation long-read sequencing, such as those offered by Oxford Nanopore Technologies and PacBio, evolutionary genomic studies can now affordably assemble highly contiguous genomes of several individuals across related species [9, 10]. The next challenge is to perform population-scale SV discovery and examine the role of SVs in population divergence and speciation.

Structural variation can occur in all parts of the genome: coding, noncoding, and repetitive regions such as transposons, telomeres, and centromeres. When they occur within coding regions, they may alter regulatory elements, introns, exons, whole genes, or multiple genes [11, 12]. Even when they do not occur within coding regions, they can change the chromatin structure and impact gene expression [13, 14]. Different types of SVs are known or predicted to have different genomic effects. Inversions can inhibit recombination between different arrangements, reducing the overall recombination rates between homologous chromosome pairs and fixing the alleles captured within their bounds [15]. Inversion-linked, cosegregating alleles can become reproductively isolated and purged through underdominant selection, due to increased sterility of heterozygous individuals [16–18]. However, a novel inversion, if adaptive, may provide enough selective advantages to outweigh its disadvantages, be selected for, and rise to high frequency within populations [19, 20]. Translocations, while less studied than other rearrangements [21], may have similar genomic effects as inversions [22]. Duplications, highly common and also likely to be selected against [23, 24], could be preserved due to their ability to acquire new function (neofunctionalization) or by retaining a subset of original function (subfunctionalization) [24–27]. Large (>50 bp) insertions and deletions, which are often genotyped as presence/absence variants (PAVs), copy number variations, or gene duplications, are also very common within genomes [8, 28]. These SVs are known to impact genes and gene structure, as well as affect phenotypes [29, 30], although many can also be neutral.

**Figure 1:**
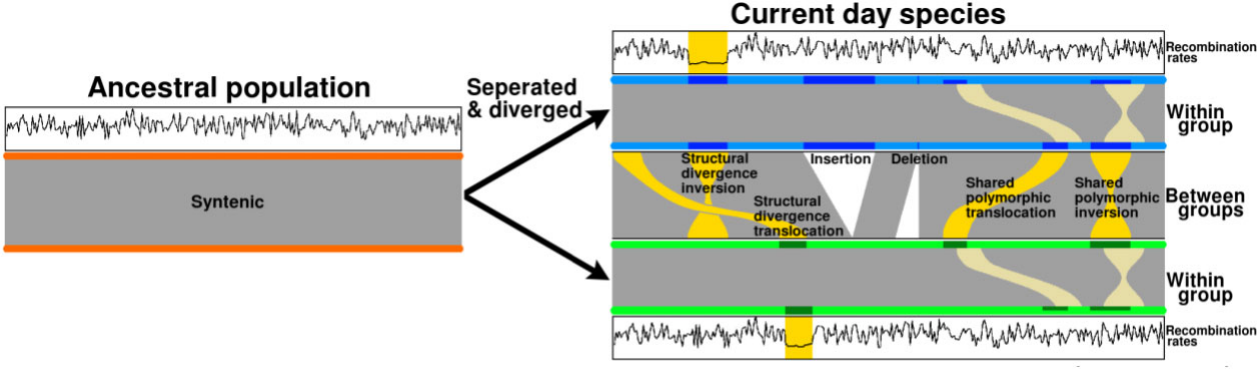
Structural variations within sister species. The once highly syntenic ancestral population separates and divides into 2 non-interbreeding groups. Structural variations, which reduce genome-wide synteny, discovered between the 2 groups may be genotyped within populations as fixed or polymorphic. When fixed in a single population, SVs become a structural divergence (SD). If polymorphic within 1 population, SVs become structural polymorphisms (SPs) or, if polymorphic in both populations, shared structural polymorphisms (SSPs). The different classes of population genotyped SVs may have different impacts on recombination rates, divergence, and adaptation.

An ancestral population, once highly syntenic, undergoes division into 2 non-interbreeding groups, with SVs emerging between them, as we have illustrated in Fig. 1. These interspecies SVs can be genotyped as fixed within 1 species, leading to structural divergence (SD), or polymorphic within 1 species, termed structural polymorphisms (SPs) [31]. Adding complexity, SVs can also be genotyped as polymorphic in both populations, referred to as shared structural polymorphisms (SSPs). To classify interspecies SVs, genotyping within both species is essential, enabling us to categorize them based on their presence/absence in population 1 and population 2 as fixed/absent (SD), fixed/polymorphic (SP), absent/polymorphic (SP), or polymorphic/polymorphic (SSP). The rate at which SVs are SD, SP, or SSP is unknown; however, rates will depend on the evolutionary distance between populations or species, effective population size, and mutation rate, among other factors. If the status of an SV remains uncertain, inferences of its impact on divergence and adaptation are difficult.

Analyzing the genomic differences between recently diverged species has revealed genome regions involved in reproductive isolation [32], adaptive genes [33], and the genome-wide landscape of diversification between and within chromosomes [34–36]. Here, using 2 closely related *Eucalyptus* species, *Eucalyptus melliodora* and *Eucalyptus sideroxylon* [37, 38], we genotype SVs within their respective populations and calculate their rates of population variability. Structural variation rates are compared to find evidence of SD, SP, and SSP. Additionally, we examine recombination rates (ρ) and fixation index (*F_ST_*) within population fixed SVs to assess allele fixation and accelerated evolution between populations.

## Results

### Genome scaffolding and annotation (repeats and genes)

We generated Hi-C data and performed Hi-C scaffolding to order, orient, and combine contigs into pseudo-chromosomes for *E. melliodora*. Hi-C sequencing generated 45.48 Gbp in 151,590,503 paired reads, giving an estimated genome coverage of 71.14×. After aligning Hi-C reads to *E. melliodora*’s contigs and identifying PCR duplicates, 18,507,548 (12.21%) read pairs were found to contain linkage information. Further examination showed that 9,612,532 (6.34%) read pairs spanned contigs, and 8,895,016 (5.87%) read pairs were contained within a single contig. Noninformative reads were chimeric, unmapped, PCR duplicates or had low mapping quality (MAPQ <30, mostly due to multimapping of short reads to repeat regions). For all Hi-C statistics, see Supplementary Table S1. Using 3D-DNA, *E. melliodora*’s contigs were scaffolded (Supplementary Figs. S1 and S2). Contigs for *E. sideroxylon* were syntenically scaffolded against *E. melliodora*’s Hi-C scaffolded genome. Both BUSCO and long terminal repeat assembly index (LAI) scores indicate that both genomes are highly complete (Table 1). Both genomes were annotated for transposable elements (TEs), simple repeats, and genes (Table 1). Transposable elements and simple repeats were annotated with genome-specific *de novo* repeat libraries. Soft repeat masked genomes were next annotated for genes.

**Table 1:** Genome assembly statistics for *E. melliodora* and *E. sideroxylon*

	*E. melliodora*	*E. sideroxylon*
Scaffolded genome size (bp)	639,266,298	592,154,182
% of genome in scaffolds	97.60%	98.15%
Scaffold N50 (Mbp)	59.47	60.48
Contig N50 (Mbp)	1.87	5.22
Contig count	564	297
BUSCO complete	98.54%	96.47%
LAI	18.31	18.70
Repetitive % (TE %)	48.50% (47.13%)	47.83% (46.58%)
Gene candidates	58,902	57,299
Proportion of genome in gene candidates	21.85%	21.04%

### Synteny and structural variation annotation

**Figure 2:**
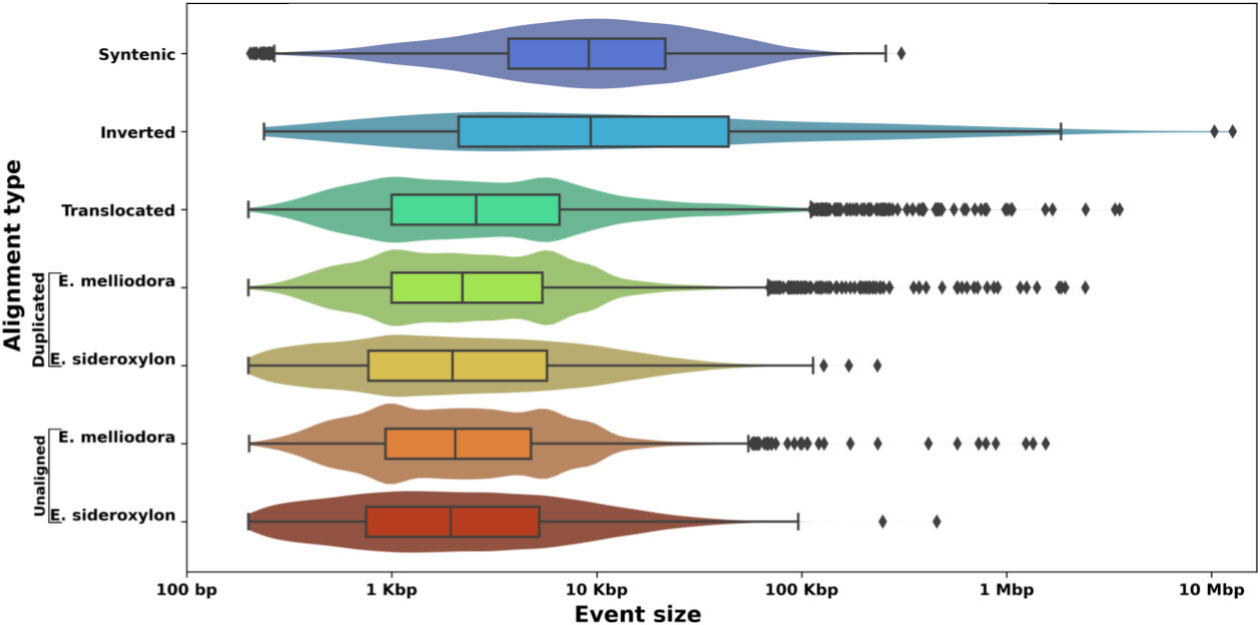
Synteny, rearranged, and unaligned event sizes. As syntenic, inverted, and translocated regions are approximately the same size within each genome (differing only by small indels), these alignment types are only shown for *E. melliodora*. Duplications and unaligned regions are unique to each genome and as such are shown for both *E. melliodora* and *E. sideroxylon*. See Supplementary Fig. S4 for all event sizes for both genomes.

Shared sequences between *E. melliodora* and *E. sideroxylon* were identified, classified as syntenic, inverted, translocated, or duplicated, and both genomes were accordingly annotated. Additionally, unaligned regions in each genome, arising from insertions, deletions, or divergence, were annotated. An estimated 85.94% of *E. melliodora*’s genome was found to be shared with *E. sideroxylon*’s genome; conversely, 87.70% of *E. sideroxylon*’s genome was found to be shared with *E. melliodora*’s genome. The majority of shared sequences were syntenic. A more detailed analysis of alignment types showed that syntenic regions are, on average, frequent and large; inversions are rare and typically very large; translocations are moderately sized and frequent; duplications are very frequent and small; and unaligned regions are very frequent and small (Table 2, Fig. 2). The distribution of synteny, inverted, translocated, and duplicated regions between the genomes of *E. melliodora* and *E. sideroxylon* was also examined (Supplementary Fig. S3). Briefly, all chromosomes exhibited a substantial number of rearrangements distributed across their entire length. Notably, chromosomes 9 and 10 were found to contain a particularly prominent inversion. These observations highlight the complexity of genome structural evolution and emphasize the need to investigate their functional implications and evolutionary significance.

**Table 2:** Proportion, number of regions, and total amount of the genome that was found to be syntenic, rearranged, and unaligned within *E. melliodora* and *E. sideroxylon* when their genomes were aligned

Genome	Statistic	Syntenic	Inversion	Translocation	Duplication	Unaligned
*E. melliodora*	Count	19,137	232	10,645	26,762	20,386
	Average size (Kbp)	16.18 ± 20.93	202.96 ± 1097.87	11.41 ± 69.65	5.25 ± 32.57	4.30 ± 7.13
	Total (Mbp)	309.60	47.09	121.49	140.63	87.74
	Proportion	49.62%	7.55%	19.47%	22.54%	14.06%
*E. sideroxylon*	Count	19,137	232	10,645	20,102	18,777
	Average size (Kbp)	16.14 ± 20.87	177.67 ± 851.99	11.29 ± 65.34	4.30 ± 33.33	3.81 ± 6.67
	Total (Mbp)	308.78	41.22	120.22	86.44	71.51
	Proportion	53.13%	7.09%	20.69%	14.87%	12.30%

### Variant calling and PCA

**Figure 3:**
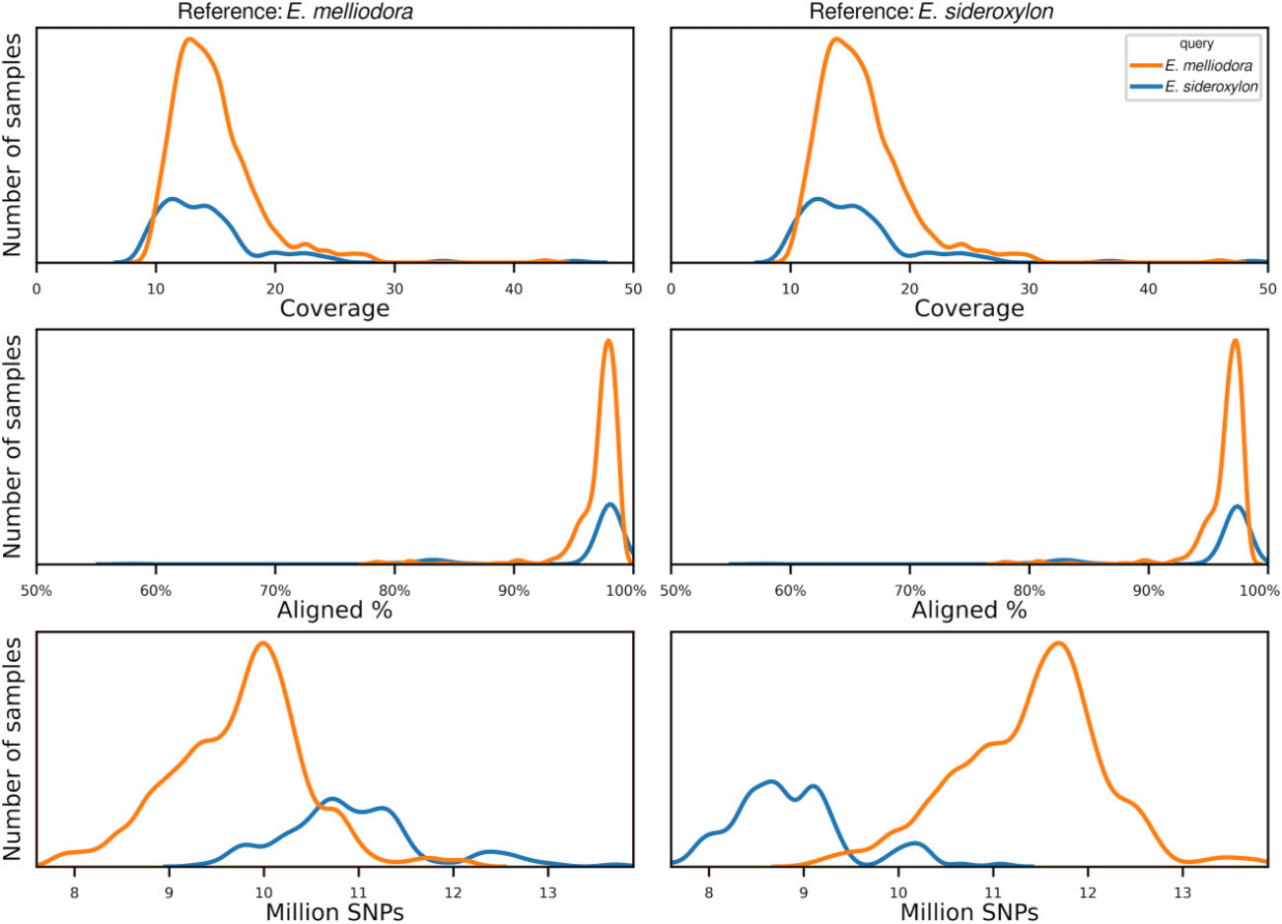
Sample coverage, alignment, and SNP distribution**s**. Left figures use *E. melliodora* as the reference, showing the per sample density of sample coverage, percentage of reads successfully aligned to reference, and the number of SNPs detected. Right figures use *E. sideroxylon* as the reference.

For every short-read sequencing dataset in the 2 populations, the total number of sequenced bases was calculated and samples that had low coverage (<10×) were removed. *E. melliodora*’s samples yielded on average 9.49 Gbp (range: 6.27–27.22 Gbp); similarly, *E. sideroxylon*’s samples yielded on average 9.10 Gbp (range: 5.82–28.87 Gbp). Examined across both populations and both reference genomes, coverage averaged 15.40× (range: 10.00×–48.7×). After aligning both population sequences to both reference genomes and filtering out samples with low alignment (< 75%), an average of 96.55% (range: 77.91%–98.80%) of reads aligned to both genomes. Variants were called for the remaining samples, resulting in 4 datasets (reference genome–population species): *E. melliodora–E. melliodora, E. melliodora–E. sideroxylon, E. sideroxylon–E. melliodora*, and *E. sideroxylon–E. sideroxylon* (Table 3, Fig. 3).

**Table 3:** Short-read sequencing, alignment, SNP, and recombination rate estimate statistics

	Reference species	*E. melliodora*		*E. sideroxylon*	
	Population species	*E. melliodora*	*E. sideroxylon*	*E. melliodora*	*E. sideroxylon*
	All samples	459	154	459	154
	Filtered samples	425	138	425	138
Estimated read coverage	Average	14.90	16.03	14.82	15.52
	Range	10.00–42.58	10.59–45.97	10.11–45.16	10.06–48.76
Read alignment	Average	97.06%	96.43%	96.32%	95.81%
	Range	78.40%–98.80%	78.70%–98.56%	77.91%–98.10%	78.38%–98.02%
SNPs (million)	Average	9.74	10.93	11.36	8.88
	Range	6.77–13.50	8.30–13.80	7.07–14.80	7.61–12.05
	Total	23.16	25.39	24.96	21.87
	Grand total	32.46		31.28	
Recombination rate	Genome-wide	0.050	—	0.049	—
estimates	Chromosome average range	0.049–0.052	—	0.047–0.049	—

**Figure 4:**
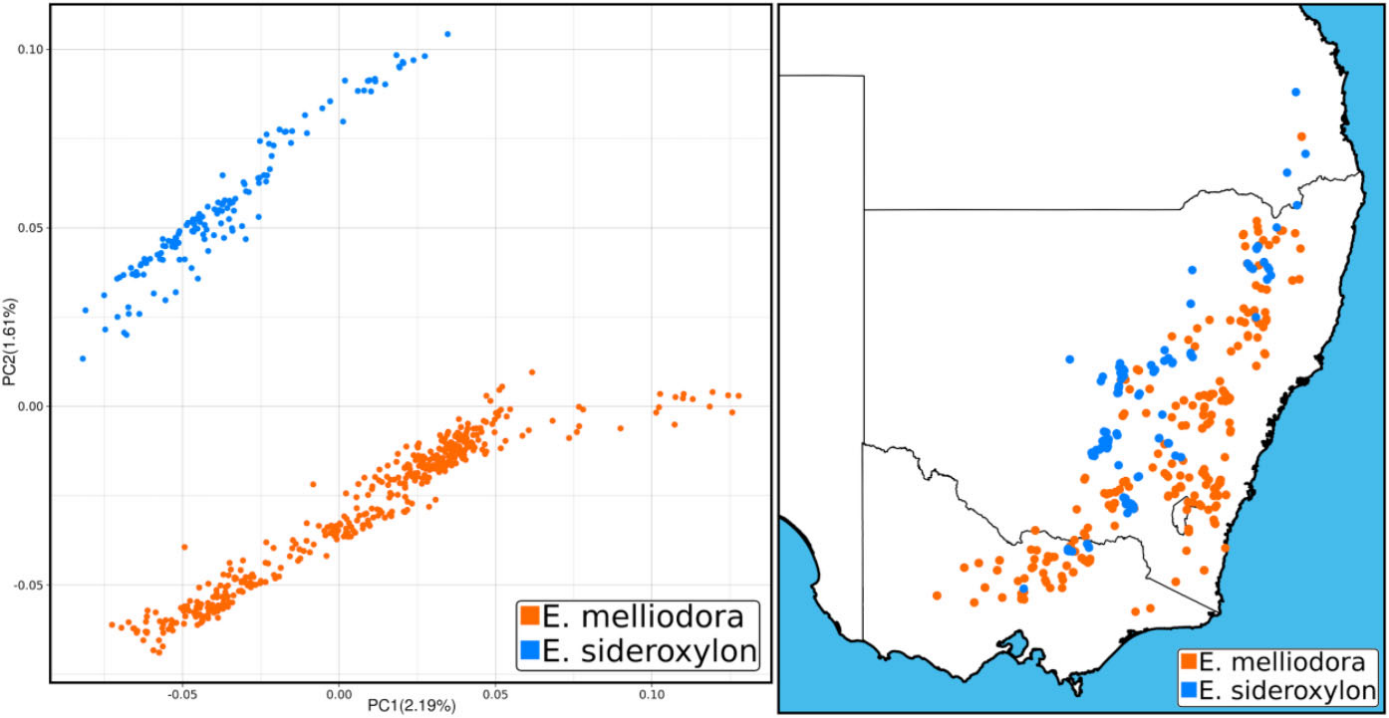
Principal component analysis (PCA) and sample distribution. Left PCA plot uses *E. melliodora* as the reference genome following the removal of mislabeled, hybrid, and outlier samples. Right map shows the spatial distribution of samples across southeastern Australia. For PCA using *E. sideroxylon* as the reference, see Supplementary Fig. S6.

Principal component analysis (PCA) identified 15 samples that were most likely misidentified or an uncharacterized hybrid, which were removed (Supplementary Fig. S5). After removal of these samples, the PCA showed 2 distinct species groups (Fig. 4). Within the combined *E. melliodora* dataset, 32.45 million sites, or 5.20% of the genome, were found to be variable. Of these single nucleotide polymorphisms (SNPs), 49.61% were found segregating within both species, 21.76% were private to *E. melliodora*, and as expected, a larger proportion, 28.63% were private to the nonreference species *E. sideroxylon*. Within the combined *E .sideroxylon* SNP dataset, we observed the same pattern; 31.28 million SNPs (5.38% of the genome) were found, of which 49.68% segregated within both species, and 20.24% were private to *E. sideroxylon*, while a larger proportion, 30.08%, was found within the nonreference species (Table 3).

### Structural variation genotyping

Interspecies SVs identified between *E. melliodora* and *E. sideroxylon* may be categorized as SD, SP, or SSP. Structural divergences are any event fixed within 1 species and absent from the other. Structural polymorphisms are any event fixed or absent in 1 species and polymorphic in the other. Shared structural polymorphisms are SVs that are polymorphic in both populations (Fig. 1). Genotyping an SV as SD, SP, or SSP requires examination within both species. While symmetric rearrangements, such as inversions and translocations, can be directly genotyped in both populations, duplications pose challenges due to their asymmetry. Although converting duplications into insertions for short-read genotyping is possible, accurately placing them within the opposite genome is difficult and may result in false-negative genotypes. Additionally, genotyping unaligned regions introduces uncertainties, especially as they may represent insertions, deletions, or divergent sequences. Short-read alignments with low mapping scores may confound genotyping of unaligned regions [39, 40]. Hence, we approach unaligned regions with caution; refrain from categorizing duplications as SD, SP, or SSP; and focus our analysis on inversions and translocations for more reliable results. All analyses are performed per allele (2 × population size), not per sample.

**Figure 5:**
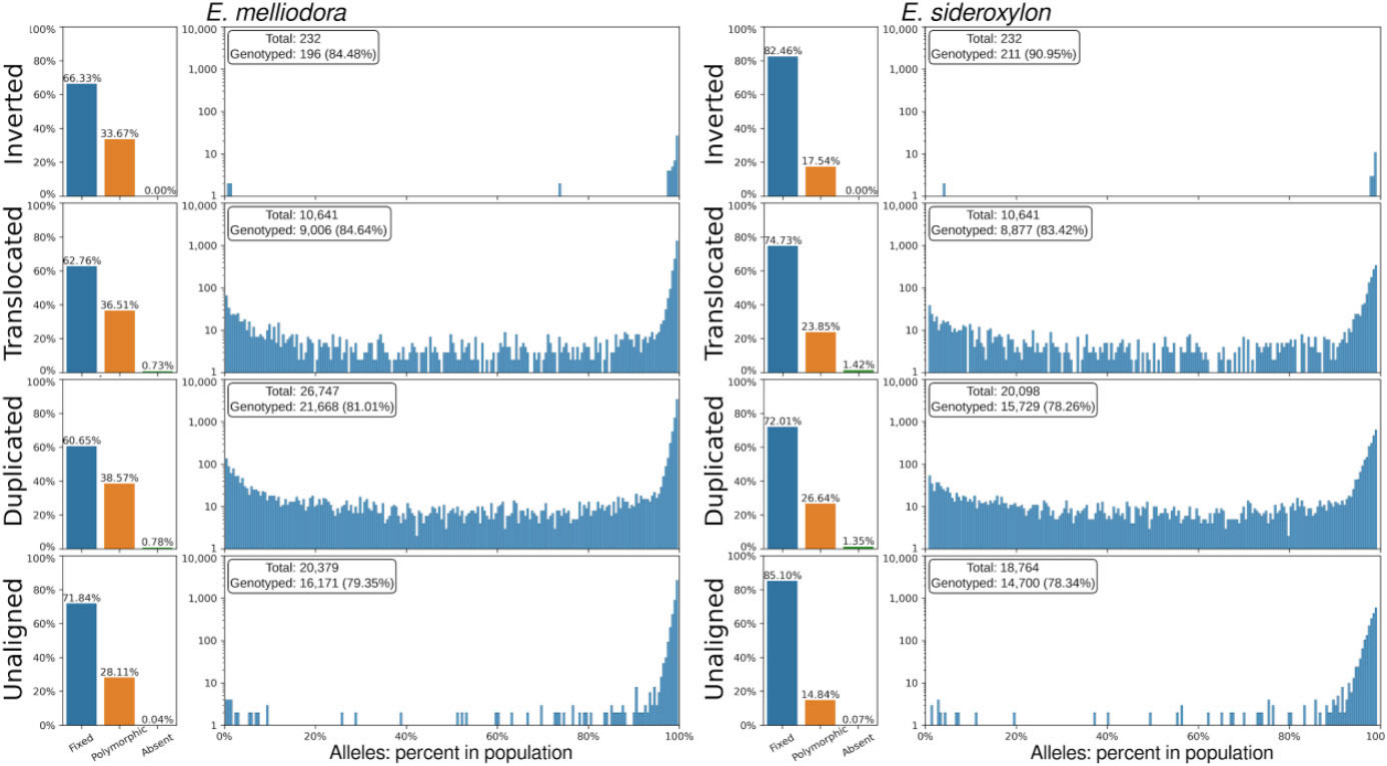
Interspecies SVs and unaligned region frequencies within *E. melliodora* and *E. sideroxylon*.

Genotyping SVs with short-read alignments resulted in the successful genotyping of 81.11% and 79.46% of SVs in *E. melliodora* and *E. sideroxylon*, respectively (Fig. 5). Most SVs were found to be fixed (60.65%–85.10%) or polymorphic (14.84%–38.57%), with the remaining small proportion (0%–1.45%) being private to the reference or assembly/scaffolding artifacts. To categorize symmetric interspecies SVs as SD, SP, or SSP, we combined the status of fixed inversions (*E. melliodora*: 130; *E. sideroxylon*: 174), polymorphic inversions (*E. melliodora*: 66; *E. sideroxylon*: 37), fixed translocations (*E. melliodora*: 5,652; *E. sideroxylon*: 6,634), and polymorphic translocations (*E. melliodora*: 3,288; *E. sideroxylon*: 2,117) across both species (Table 4). The analysis revealed that most inversions and translocations were either fixed in both species or not successfully genotyped in both species. The remaining proportion consisted of SPs or SSPs, and a small number of SD. For details on inversion and translocation classification within both species and subsequent SD, SPP, or SP classification, see Supplementary Table S2.

**Table 4:** Categorization of interspecies inversions and translocations as SD, SP, and SPP

	Shared structural polymorphism	Structural polymorphism	Structural divergence		Private to reference or artifact
Inversion	18 (7.79%)	60 (25.98%)	*E. melliodora*	0	153 (66.23%)
			*E. sideroxylon*	0	
Translocation	910 (8.81%)	2,563 (24.80%)	*E. melliodora*	16 (0.15%)	6,825 (66.06%)
			*E. sideroxylon*	18 (0.17%)	

Examination of polymorphic SVs revealed a bimodal distribution of alleles containing the SV (Fig. 5). Polymorphic SVs were either very frequently genotyped (>90%) or very infrequently genotyped (<10%) within the 2 species. However, while bimodally distributed, the very frequent SV peak was found to be much higher than the very infrequent SV peak.

### Structural variation linkage

**Figure 6:**
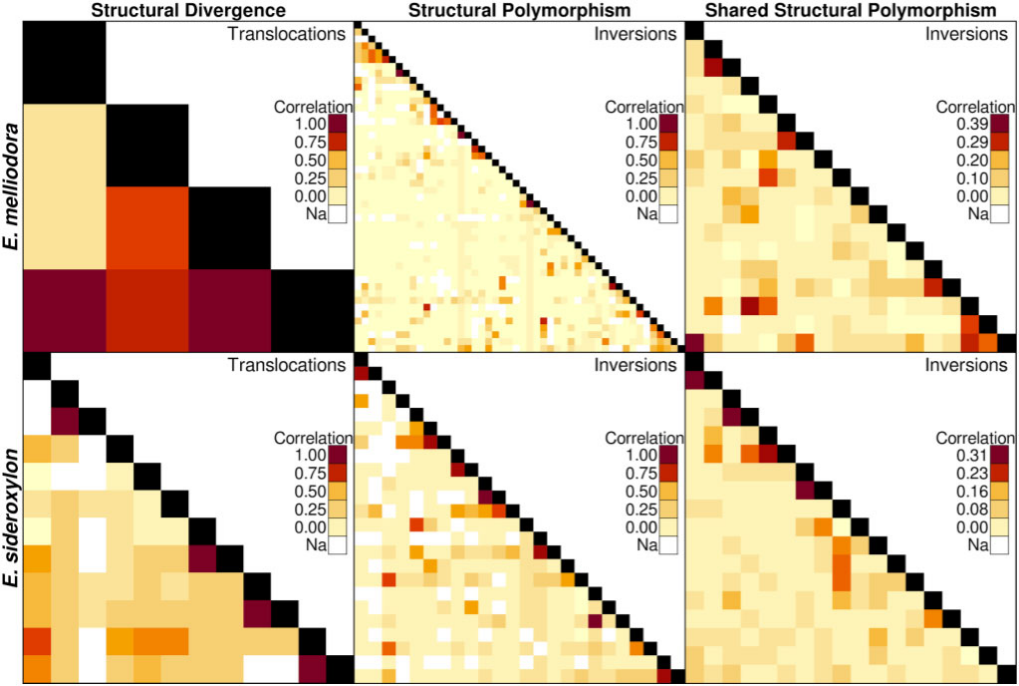
Correlation of SVs between samples. A positive correlation between SV implies that SVs exhibit a nonrandom association and suggests that these variants tend to co-occur within the population. Categories of SV not present were either empty, as in the case of inversion SD, or contained too many SV to visualize clearly, as in the case of translocation SP and translocation SSP. Undefined correlations, resulting from the failure of short reads to resolve presence/absence of SVs, were removed.

Linked variations are those that co-occur more often than would be expected by random chance. Structural variations may be linked by physical proximity, drift, or evolution. Evolutionarily linked SVs are likely to contribute to an individual’s survivability and be required for gamete viability and/or the offspring’s adaptive potential. To find evidence of SV linkage, we measured correlations among all inversions and translocations for all individuals within both species. For efficient analysis, inversions and duplications were grouped by type (SD, SP, and SSP). Inspection of the resulting correlation heatmaps shows 40,118 SVs are linked (*R*^2^ ≥ 0.6) across all categories (Fig. 6). To examine the potential role of physical proximity on SV linkage, we examined the distance between correlated SV pairs. Of SV pairs, 89.24% were found on different chromosomes. When on the same chromosome, SVs were at least 221 Kbp separated.

### Shared structural polymorphisms Clusters of Orthologous Groups (COG) terms

**Figure 7:**
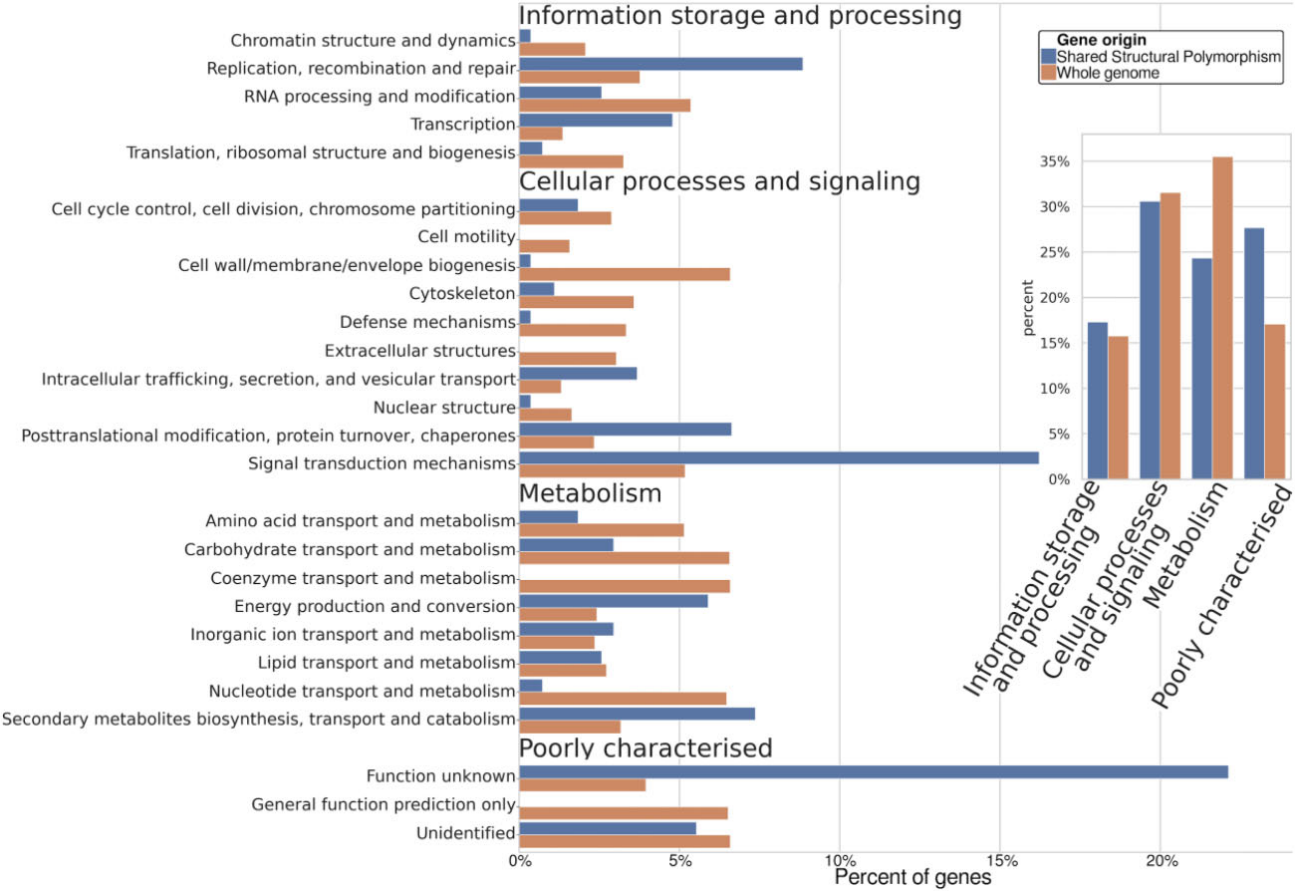
Clusters of Orthologous Groups (COG) terms for all genes and genes found within SSPs.

As SSPs are likely ancestral SVs that have survived drift, underdominant selection, and lineage divergence, they may contain genes of adaptive or other evolutionarily significant value. After attempting to functionally annotate all genes across the genomes and placing them within Clusters of Orthologous Groups (COG) categories [41], 247 of the total 281 gene candidates in SSPs were annotated (Fig. 7). These genes were enriched for DNA replication, DNA recombination, DNA repair, posttranslational modification, protein turnover, chaperones, signal transduction, intercellular communication, and unexplored aspects of biology. SSP genes were found to be underrepresented in categories related to fundamental cellular functions, such as protein synthesis, defense against pathogens, maintaining cellular integrity, providing structural support, and regulating crucial molecular processes involving amino acids, nucleotides, and coenzymes. Additionally, we performed a Gene Ontology (GO) [42] enrichment test for all genes identified in SSPs. We found 51 GO terms (shared: 31; *E. melliodora*: 11; *E. sideroxylon*: 9) to be significantly less represented in SSP genes compared to all genes, with no GO terms found to be significantly higher. GO terms were associated with biological process (28), cellular component (15), and molecular function (8). Further details can be found in Supplementary Table S3.

### Effect of syntenic, rearranged, unaligned regions and genes on recombination rate (ρ)

After annotating SVs in both species and determining their frequencies, we calculated ρ for fixed SVs longer than 2 Kbp across the reference genomes. Prior to these calculations, we phased SNPs, initially achieving 20.56% linkage within haplotype blocks using read alignments, and then finalized the phasing using a hidden Markov model (HMM)–based approach. After separation of SNPs into parental haplotypes, we found that *E. sideroxylon* consistently exhibited higher and more variable ρ compared to *E. melliodora*. Chromosome-specific recombination rates displayed notable variability without discernible patterns (Table 3, Supplementary Table S4, and Supplementary Figs. S7 and S8).

**Figure 8:**
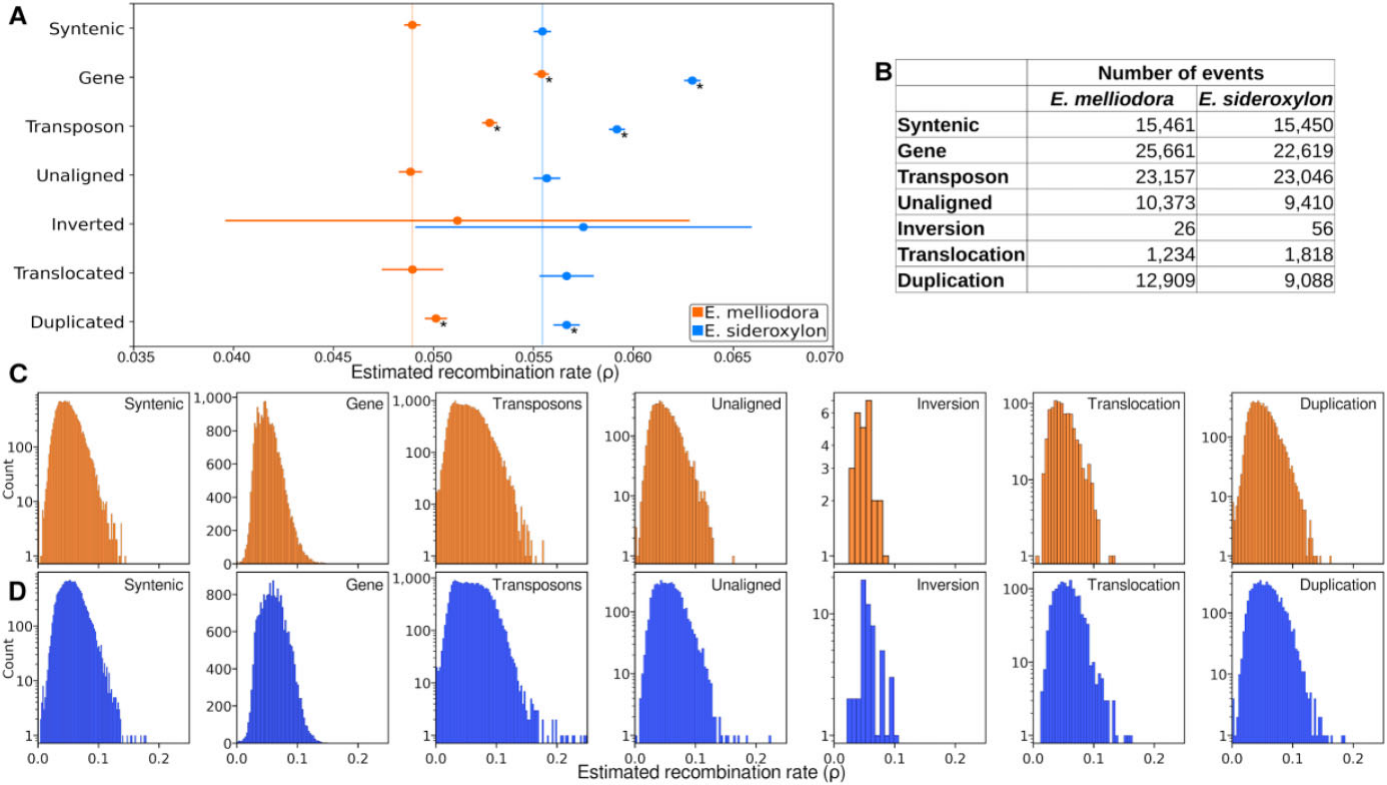
Tukey’s test for estimated recombination rates of fixed SVs, unaligned regions, genes, and transposons. (A) Mean and 95% confidence interval for all events. Vertical lines show average ρ for syntenic regions. Asterisk (*) indicates region types that are significantly different from syntenic regions (*P* ≤ 0.05). (B) The number of events included in the analysis. (C) Estimated recombination rate distribution for *E. melliodora*. (D) Estimated recombination rate distribution for *E. sideroxylon*.

An initial analysis of variance assessment indicated differences in ρ for our different categories of genome regions, for both species (*P* value; *E. melliodora*: 8.35 × 10^−276^ and *E. sideroxylon*: 1.85 × 10^−272^). To determine if any region type(s) were contributing to differences in ρ, we performed Tukey’s test. Tukey’s test adjusted *P* values to account for the total species error rate. Tukey’s test for *E. melliodora* revealed that, in comparison to syntenic regions, average ρ was higher for genes, transposons, inversions, and duplications (Fig. 8A). However, statistically significant differences were observed only for genes, transposons, and duplications. Notably, our results suggest that genes and transposons undergo recombination more frequently than other genomic regions. Consequently, the sequences within genes and transposons passed onto offspring may be the most highly diverse among the regions tested. Furthermore, duplications showed higher values than translocations and unaligned regions. Inversions exhibited a wider confidence interval (CI) due to their lower number of events. A similar pattern was observed by Tukey’s test for *E. sideroxylon*. While genome-wide statistical observations of ρ were unrevealing, many SVs were observed having ρ less than the mean syntenic (Fig. 8C, D). Detailed significance testing results are presented in Supplementary Table S5.

### Effect of syntenic, rearranged, unaligned regions and genes on fixation index (*F_ST_*)

**Figure 9:**
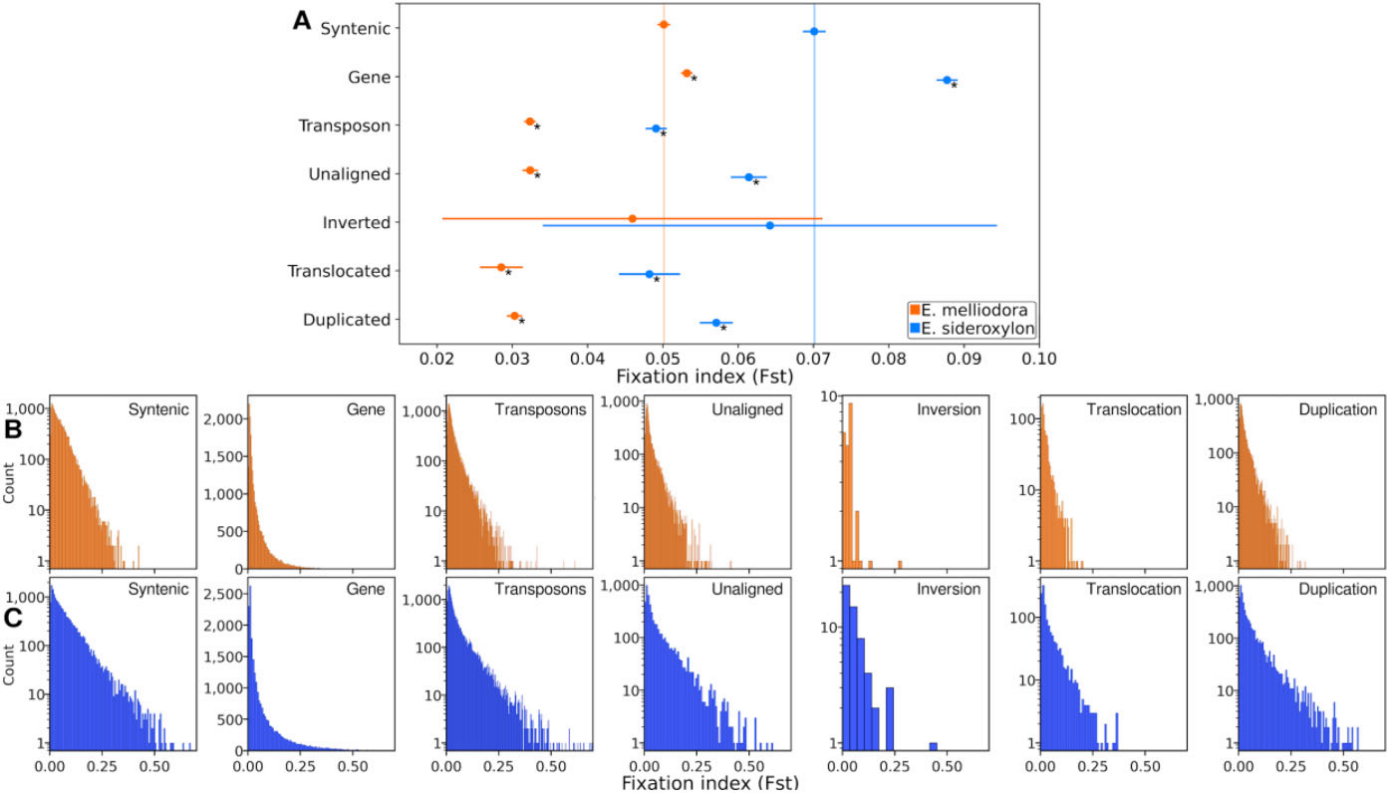
Tukey’s test for fixation index of fixed SVs, unaligned regions, genes, and transposons. (A) Average *F_ST_* and 95% confidence intervals calculated from average *F_ST_* values for all regions. For each reference genome, SNPs from both species were combined and *F_ST_* calculated. Vertical lines show average *F_ST_* for syntenic regions. Asterisk (*) indicates region types that are significantly different from syntenic regions (*P* ≤ 0.05). (B) Fixation index distribution for *E. melliodora*. (C) Fixation index distribution for *E. sideroxylon*. For events counts see Fig. 8B.

As per our examination of ρ, we calculated the average *F_ST_* for all fixed SVs, as well as genes and transposons greater than 2 Kbp in length, and performed Tukey’s test (Fig. 9A). Syntenic regions were used as the reference point to evaluate the extent of genetic differentiation of SVs. Using *E. melliodora* as the reference, all region types had significantly less divergence between species except genes and inversions. Genes had significantly more divergence and inversions were sparse and as such had a wide confidence interval. A similar pattern was observed for *E. sideroxylon*. While genome-wide statistical observations of *F_ST_* were unrevealing, many SVs were observed having *F_ST_* less than the mean syntenic (Fig. 9B, C). Examination of *F_ST_* histograms for all event types showed a left-shifted Poisson distribution, with many events having low *F_ST_* scores. For detailed significance testing results, refer to Supplementary Table S6.

### Effect of syntenic, rearranged, unaligned regions and genes on SNPs

SNP density can significantly impact the precision and resolution of both ρ and *F_ST_* [43–45]. Higher SNP density enables finer-scale mapping of recombination events and more accurate population differentiation measurements, while lower SNP density gives coarser results with reduced precision. Due to inconclusive results in both ρ and *F_ST_* analyses, we examined SNP densities of SVs, genes, and TEs.

**Figure 10:**
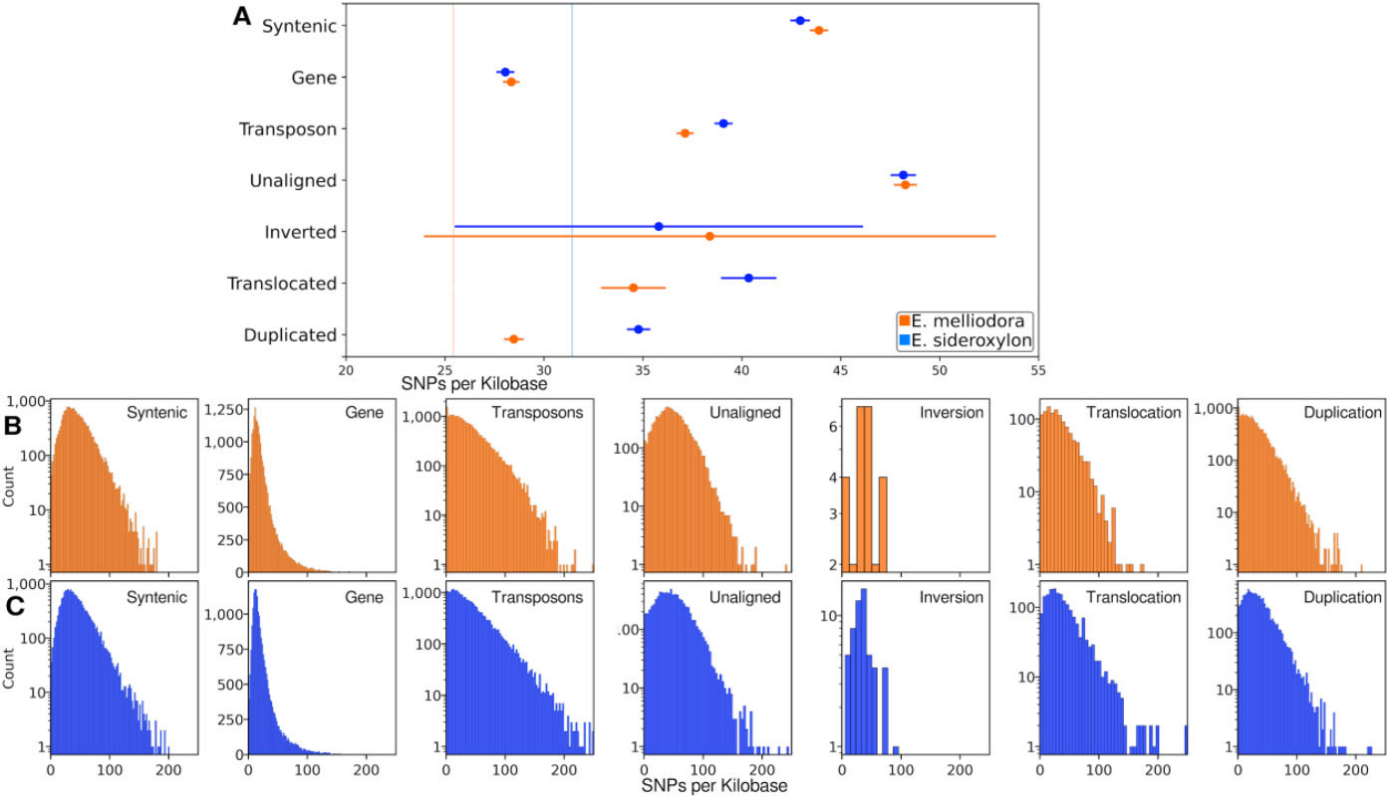
Tukey’s test for SNP density of fixed SVs, unaligned regions, genes, and transposons. (A) Mean and 95% confidence interval for all events. Vertical lines show average SNP density for syntenic regions. (B) SNP density distribution for *E. melliodora*. (C) SNP density distribution for *E. sideroxylon*. For events counts, see Fig. 8B.

As per our ρ and *F_ST_* analyses, we used Tukey’s test and histograms to examine the differences in SNP densities for all fixed SVs and genes and transposons greater than 2 Kbp in length (Fig. 10A–C). For detailed significance testing results, refer to Supplementary Table S7. Reassuring to our SV annotation method, unaligned regions were the most diverged region type, containing the largest number of SNPs. Similarly reassuring for our annotation method, genes were the least diverged, containing the fewest SNPs. No significant correlations between the number of SNPs and ρ were observed. Notably, genes, transposons, and duplications had high ρ, while only transposons had a high SNP density. Conversely, unaligned and translocated regions had low ρ, while only translocations had few SNPs. Similarly, no distinct correlations between SNPs and *F_ST_* values were observed. Genes, despite having few SNPs, contained high *F_ST_* values, whereas unaligned regions, with many SNPs, displayed low *F_ST_* values. Translocated regions, with an intermediate number of SNPs, also exhibited low *F_ST_* values. Although SNP densities contribute to the complex pattern of genomic differentiation, they showed no clear association with ρ and *F_ST_* calculations.

## Discussion

Structural variations are a major form of genomic variation, affecting more nucleotides than SNPs [46]. Despite their prominence, the functional and evolutionary impacts of SVs remain poorly understood [47–49]. To date, most population-scale SV studies have focused on within-population SV discovery and association with environments or phenotypes [50, 51]. Several studies have also directly examined SV and their contribution to functional changes [52, 53]. Here we genotyped interspecies SVs and described their frequencies within and among both species. Of particular novelty is our comparison of translocations and inversions, symmetric SVs that may be present within 1 or both species and at different frequencies. Between our recently diverged *Eucalyptus* species pair, our results demonstrate that SVs contribute to genome divergence, intraspecies genetic diversity, and shared genetic diversity. Potentially of great interest are SSPs; these large mutations predate lineage divergence and remain polymorphic within both species, potentially containing locally adaptive or otherwise important genes and allele combinations. Additionally, examination of average ρ and *F_ST_* within fixed SVs demonstrates the variable effects of these genetic variations on genome differentiation and recombination.

Genetic mutations that promote and reinforce lineage divergence are the genetic basis of reproductive isolation, which is essential to the process of speciation. Structural mutations, by affecting recombination, phenotypes, or altering/removing/subfunctionalizing genes, are of particular importance to speciation processes [5]. Barrier complexity and asymmetry are underappreciated components of reproductive isolation. Barrier complexity involves the combinatorial interplay of genetic barriers that collectively reduce reproductive success between individuals [54]. Successful offspring are survivors of genetic combinations, possessing genomes sufficiently free from barrier loci (genomic loci that create barriers to gene flow among populations [55]) to allow reproduction to occur. Barrier asymmetry refers to the relative effectiveness of reproductive barriers between 2 groups, resulting in different hybridization success rates [56]. *E. melliodora* and *E. sideroxylon* are known to hybridize, and successful hybridization likely results from the complex interplay between the numerous SDs, SPs, and SPPs that come together in a particular hybrid. Evidence of linked SDs, SPs, and SPPs was observed within both *Eucalyptus* species. These linked SV combinations may be required for reproductive success, or there could be some other fitness consequence that is maintaining selection for that linked state. Barrier SVs potentially exhibit a higher degree of reproductive isolation compared to non-SV regions, increasing genetic differentiation within these loci [57–59]. However, variation in *F_ST_* did not provide sufficient evidence on average to support this conclusion, possibly due to the recent divergence of our species and the importance of only a few key interacting loci.

Similar to reproductive isolation, understanding how all types of genetic mutations contribute to the creation and maintenance of genetic diversity is crucial to understanding how organisms improve fitness and adapt to their changing environments [8, 60, 61]. Inversions and translocations aid in adaptive evolution by fixing allele combinations, duplications contribute to the development of new genes, and insertions and deletions, often described as PAVs, modify gene expression and gene content [62–64]. A substantial number of inversions and translocations were successfully genotyped within both species. Most inversions and translocations were SP or SSP, making them candidates for exploring adaptive genes and alleles. Of particular note are SSP inversions and translocations, which showed evidence of gene enrichment in potentially adaptive genes.

Duplications are known to be highly common and an important source of evolutionary novelty [65–66] and were the most common type of SV in our analysis. Most duplications were found to be fixed, with the remainder being almost entirely polymorphic. Given their asymmetry, duplications were genotyped only within their respective host genomes, resulting in an inability to categorize them as SD, SP, or SSP. Nonetheless, duplications successfully genotyped in our study are potential candidates for adaptive loci, likely having withstood the influences of genetic drift and purifying selection. Predicting the adaptive effects of unaligned regions presents a significant challenge, given their potential to encompass insertions, deletions, or highly divergent sequences. When unaligned regions result from highly divergent sequences, short reads will align poorly, confounding genotyping [39, 40]. Genotyped as deletions, most unaligned regions were fixed and the remainder highly frequent. Fixed unaligned regions may correspond to highly divergent regions or deletions in the genome of the other species. Polymorphic unaligned regions could indicate insertions within the host species genome or deletions within the genome of the other species. These difficult to interpret regions may be PAVs, adaptive loci, or selectively neutral or deleterious loci undergoing potential purifying selection. Further investigations are essential to uncover their precise roles and implications.

It is now clear that SVs are of great evolutionary importance and must be considered when studying genetic diversity and genome evolution [64]. To better evaluate the impact of SVs on evolution, a combination of interspecies and intraspecies studies is crucial. While structural polymorphisms may be reproductive barriers or adaptive loci, they could also be neutral or deleterious, especially as these species separated very recently. Given that SVs are rarely conserved (i.e., typically purged over short time scales) [67, 68], and many of the SVs examined here were genotyped at high frequencies, there is potential for common SVs to be investigated for functional associations with traits or environments, thus warranting future scrutiny regarding their contribution to adaptive evolution. Future studies are needed to test whether these SVs contribute to adaptive evolution. To assess their potential role as barrier loci, breeding experiments could be employed. A problem encountered here was the number of SVs within individuals that could not be genotyped. Many statistical tests require all samples to be genotyped for all genetic variants, employing imputation to fill in missing genotypes. However, all current imputation processes are designed for SNPs captured within haplotype blocks. Statistical association programs that can incorporate SVs are needed. With the decreasing cost and increasing accuracy of long-read sequencing, particularly Oxford Nanopore [69], future studies could use high-throughput long-read sequencing to overcome the limitations of short-read SV genotyping. However, advances in analysis software are still a limiting constraint for fully understanding the contribution of SVs to adaptive evolution and speciation.

## Methods

### Population sampling and sequencing

Yellow box (*E. melliodora*) and red ironbark (*E. sideroxylon*) are closely related eucalypts of the box-gum grassy woodland endangered ecological community. These species are often found growing in sympatry or parapatry and widely hybridize throughout their ranges in southeastern Australia. Additionally, these *Eucalyptus* species have been used in genetic adaptation and introgression studies [70–72], contributing to the availability of large genetic datasets for these species, making them ideal candidates for our study. We collected 472 *E. melliodora* and 180 *E. sideroxylon*, all samples being wild and undomesticated. Samples were environmentally stratified to capture major clines in climate-adaptive genomic variation across the species’ distributions. GPS data were recorded for each sample (Fig. 4), and leaf material was dried in silica desiccant.

Twenty 3-mm disc punches (UniCore, Qiagen) from each leaf sample were placed in mini-tubes with a 3-mm ball bearing, frozen with liquid nitrogen, and ground in a TissueLyser II (Qiagen). Genomic DNA was extracted using a 96-well plate column-based kit (Stratec Invisorb DNA Plant HTS 96 Kit/C), according to the manufacturer’s instructions (Stratec SE). DNA was quantified using an Infinite M1000 PRO Tecan fluorescence microplate reader (Tecan Trading AG) and standardized to 1 ng/µL, using a liquid-handling robot. Library preparation was performed using a modified Illumina Nextera DNA Library Prep Kit workflow, which is available in Protocols.io and described in Jones et al. [73]. Libraries were then quantified using GXII and Quant-iT and pooled for equal representation. Prior to size selection, samples were concentrated using 2× binding buffer and 100 µL Sera-Mag Speedbeads Carboxylate-Modified Particles (Thermo Scientific). Size selection was then performed on a Pippin Prep (Sage Science), for 400- to 650-bp fragments. Samples were again concentrated with 2× binding buffer and 100 µL Sera-Mag beads, then quantified using both a Qubit Fluorometer (Thermo Scientific) and Bioanalyzer high-sensitivity DNA chips (Agilent Technologies). Whole-genome sequencing was performed on an Illumina NovaSeq 6000 (RRID:SCR_016387), 150-bp paired-end sequencing, by Novogene (HK) Co., Ltd.

### Genome scaffolding

We performed Hi-C scaffolding, grouping, ordering, and orienting of our previously assembled *E. melliodora* genome into pseudo-chromosomes [31]. The initial draft was created by extracting and sequencing high-molecular weight DNA [74] on the Oxford Nanopore Technologies MinION platform, and assembling with Canu as previously described [75]. Subsequently, fresh leaves were obtained from the reference tree, and a proximity ligation library for chromosome conformation capture was created with a Phase Genomics Proximo Hi-C (Plant) Kit (version 4), according to the manufacturer's instructions (document KT3040B). The restriction enzymes DpnII, HinFI, MseI, and DdeI were used to digest the genome. Sequencing was performed on an Illumina NovaSeq 6000,150 bp paired end sequencing. Hi-C scaffolding began by aligning all Hi-C reads to *E. melliodora*’s contigs using bwa mem (RRID:SCR_022192) [76] (version: 0.7.17; parameters: -5SP). Next, PCR duplicates were identified with Samblaster (RRID:SCR_000468) [77] (version: 0.1.26). Linkage information captured within Hi-C reads was assessed with Juicer (RRID:SCR_017226) [78] (version: 1.6) and scaffolding was performed using 3D-DNA (RRID:SCR_017227) [79] (version: 190,716; parameter: -i 1000). Due to the high repeat content, Hi-C read coverage was highly variable and resulted in poor quality scaffolding. To account for variability in read coverage, we ran 3D-DNA with “–editor-repeat-coverage 5,” altering the misjoin detection threshold. After initial scaffolding the Hi-C contact map was manually edited with Juicebox (RRID:SCR_021172) [80] (version: 2.16). Briefly, the Hi-C contact heatmap was examined for incorrectly joined and separated scaffolds. For example, scaffolds 2 and 3, and 4 and 5 (Supplementary Fig. S1) were manually joined, as indicated by their boundaries (blue boxes) disagreeing with the surrounding heatmap. Additionally, contigs displaying strong off-diagonal signals were reviewed, and if the off-diagonal signal was stronger than the diagonal signal, they were relocated to the origin of the off-diagonal signal. Previously assembled contigs for *E. sideroxylon* [31] were scaffolded with RagTag [81] (version: v2.1.0) using synteny to our Hi-C scaffolded *E. melliodora* genome.

Genome completeness was measured with BUSCO (RRID:SCR_015008) [82] (version 5) and long terminal repeat assembly index [83] (LAI). BUSCO scores genome completeness by identifying and reporting on the proportion of lineage specific highly conserved single-copy genes; more complete genomes have a high proportion of identified BUSCO genes. LAI identifies long terminal repeat (LTR) sequences and reports on the proportion that are intact. Within their publication, Ou et al. [83] established that LAI scores of <10 correspond to draft genomes, scores of 10–20 indicate reference genomes, and scores of 20 or higher represent gold-quality genomes.

### Genome annotation

Genomes were annotated for TEs using genome-specific, *de novo* repeat libraries created with EDTA (RRID:SCR_022063) [84] (version: 1.9.6) and RepeatMasker (RRID:SCR_012954) [85] (version: 4.1.1). RepeatMasker additionally annotated our genomes for simple repeats. Repeat masked genomes were next annotated for genes using BRAKER2 (RRID:SCR_018964) [86] (version 2.1.6). BRAKER2 was run with ProtHint (RRID:SCR_021167) [87] (version2.6.0) and GeneMark-EP [87] (version: 4). ProtHint analyzed training proteins to determine their evolutionary distance to the genome, aiding GeneMark-EP to train a gene detection model. Training protein sequences were obtained from the NCBI [88] and included all available transcripts for Myrtaceae (Taxonomy ID: 3931) and *Arabidopsis thaliana* (Taxonomy ID: 3702).

Candidate genes were functionally annotated for eggNOG orthogroup, COG category, GO term, KEGG term, and PFAM using eggNOG-mapper [89] (version: 2.1.12; parameters: -m diamond –itype CDS –tax_scope Viridiplantae). GO terms were extracted from all eggNOG annotated genes and a GO term enrichment analysis performed using GOATOOLS: A Python library for Gene Ontology analyses [90] (version: 1.3.11).

### Synteny and structural variation annotation

Shared sequences were identified between genomes by alignment with NUCmer (parameters: –maxmatch -l 40 -b 500 -c 200), from the MUMmer (RRID:SCR_018171) [91] (version: 3.23) toolset. NUCmer identifies all shared 40-mers between the 2 genomes and joins all 40-mers within 500 bp into single alignments. After aligning the 2 genomes, MUMmer’s delta-filter (parameters: -i 80 -l 200) tool removes all alignments <200 bp and with an identity <80%. A low sequence identity score (80%) was used due to the high heterozygosity of *Eucalyptus* genomes [71], and a higher score may incorrectly filter out real alignments. Using SyRI (RRID:SCR_023008) [92] (version: 1.5), filtered NUCmer alignments were analyzed and subsequently genomes were annotated for syntenic, inverted, translocated, duplicated, and not-alignable regions. Karyotype plot was created using plotsr [93] (version: 0.5.4).

All inversions, translocations, duplications, and unaligned regions described by SyRI were genotyped for all 563 samples within both species using Paragraph [94] and our short-read alignments.

A 0/1/2 matrix was created for all genotyped SV within both species and for all categories of SV. Using the R [95] function Cor, the correlation between SVs of interest was calculated and visualized with a heatmap.

### Alignment and variant calling

Raw population sequences were trimmed (sequencing adaptors and barcodes), quality filtered (average quality score <20), and merged (overlapping read pairs were combined into single reads) using AdapterRemoval (RRID:SCR_011834) [96] (version: 2.3.0). Genome coverage was estimated for each sample, and samples with low coverage (<10×) were removed. Quality filtered reads were next aligned to both reference genomes (*E. melliodora* and *E. sideroxylon*) using bwa mem (parameters: -p). Samples with <75% alignment were then removed. Aligned reads for all remaining samples were variant called with BCFtools (RRID:SCR_005227) [97] (version: 1.12) mpileup (parameters: MAPQ >30, base quality >15). The default mutation rate (0.0011) was increased to 0.01, making variant calling more robust when calling low-coverage heterozygous SNPs. Variant files were then merged, resulting in 4 datasets (reference genome–population species): *E. melliodora–E. melliodora, E. melliodora–E. sideroxylon, E. sideroxylon–E. melliodora*, and *E. sideroxylon–E. sideroxylon*.

### Variant filtering

Using BCFtools norm [97], multiallelic variants for each variant dataset were decomposed into multiple single variants. Decomposed variants were filtered, removing variants present in <10% of samples and with fewer than 20 supporting reads, within each dataset using the BCFtools view. Variants were next recomposed, all remaining multiallelic variants rejoined, and each dataset further filtered to remove all indels and multiallelic SNPs [98].

High-quality, biallelic SNP datasets for each reference genome were combined and a PCA performed with PCAngsd [99] (version: 1.10). Visual inspection of PCA plots allowed identification and removal of hybrids, outliers, and incorrectly labeled samples.

### SNP phasing and recombination calculation

Before computing ρ (estimated recombination rate) within our 4 datasets, SNPs first required phasing. Phasing links each variant allele, placing them into haplotype blocks, separating maternal and paternal variants. As the linkage information provided by paired-end short reads is not capable of phasing all SNPs, a 2-step phasing process was used. First, individual samples were extracted from species variant files into a single sample variant file and using read alignments, SNPs, when possible, were phased with WhatsHap (RRID:SCR_025319) [100] (version: 1.7). Second, partially phased sample variant files were re-merged and the HMM phaser SHAPEIT4 (RRID:SCR_024335) [101] (version: 4.2.2) inferred haplotypes and phased the remaining unphased SNPs. Parameters (–use-PS 0.0001 –mcmc-iterations 6b,1p,1b,1p,1b,1p,1b,1p,8 m –pbwt-depth 6 –sequencing) specified for SHAPEIT4 were optimized by balancing maximum accuracy and runtime. At the completion of this 2-stage phasing approach, all SNPs for each dataset were phased. After phasing, ρ was calculated for each dataset using LDJump [102] (parameters: alpha = 0.05; version: 0.3.1), specifying a window size of 1 Kbp. LDJump made use of LDHat (RRID:SCR_006298) [103] (version: 2.2a) to decrease runtime.

As low-frequency SVs are unlikely to have a detectable effect on ρ, we considered only fixed SVs and excluded events shorter than 2 Kbp, as ρ was calculated within 1-Kbp windows. We also assessed the impact of genes and transposons larger than 2 Kbp on ρ. Prior to ρ calculations, we phased SNPs, initially achieving 20.56% linkage within haplotype blocks using read alignments, and subsequently completing phasing with an HMM-based approach.

### Fixation index (*F_ST_*)

To measure the amount of shared genetic diversity that exists between *E. melliodora* and *E. sideroxylon*, we combined SNPs for both populations under each reference and calculated the fixation index (*F_ST_*). The fixation index, calculated per SNP, scores the amount of genetic differentiation between populations or species and ranges from 0 to 1, where 0 indicates no difference in allele frequencies and 1 indicates a fixed difference. In real-world usage, per SNP *F_ST_* values are typically far below 1, even in the case of isolated populations and should be interpreted relative to the study [104]. Here we use them to quantify how similar, or dissimilar, all region types are between *E. melliodora* and *E. sideroxylon*. Filtered SNP datasets were combined for each reference genome, and subsequently *F_ST_* was calculated for each SNP using PLINK (RRID:SCR_001757) [105] (version: 1.9). Per SNP, *F_ST_* values were averaged for each region of interest for further analysis.

## Additional Files

**Supplementary Fig. S1**. Hi-C scaffolding of *E. melliodora*’s contigs with 3D DNA (parameter: “–editor-repeat-coverage 5, -i 1000). Due to a high repeat content, Hi-C read coverage is highly variable, resulting in poor scaffolding. Hi-C contacts are visualized with Juicebox.

**Supplementary Fig. S2**. Manually curated, final, Hi-C contact map of *E. melliodora*’s contigs with 3D DNA (parameter: “–editor-repeat-coverage 5, -i 1000). Due to a high repeat content, Hi-C read coverage is highly variable, resulting in poor scaffolding. Hi-C contacts are visualized with Juicebox.

**Supplementary Fig. S3**. SyRI annotations.

**Supplementary Fig. S4**. Synteny, rearranged, and unaligned event sizes. Duplications and unaligned regions are unique to each genome and as such are shown for both *E. melliodora* and *E. sideroxylon*.

**Supplementary Fig. S5**. Raw PCA plots. Left figure uses *E. melliodora* as the reference, and the right figure uses *E. sideroxylon* as the reference.

**Supplementary Fig. S6**. Clean PCA plot, *E. Sideroxylon* as reference.

**Supplementary Fig. S7**. *E. melliodora* recombination.

**Supplementary Fig. S8**. *E. sideroxylon* recombination.

**Supplementary Table S1**. *E. melliodora* Hi-C summary stats, produced by Juicer.

**Supplementary Table S2**. Status (fixed, absent, or polymorphic) of inversions and translocations within both species and subsequent SD, SPP, or SP classification.

**Supplementary Table S3**. Shared structural polymorphism gene enrichment. The concentration of GO terms within genes found in SSPs was tested against all gene GO terms. Significantly higher or lower GO terms are listed.

**Supplementary Table S4**. Recombination rate estimates. Recombination rates were calculated in 1-Kbp windows and averaged across chromosomes. Rates are shown with standard deviation. Chromosomes colored with darker green have higher average recombination rates.

**Supplementary Table S5**. Pairwise rho Tukey’s test *P* values. Green indicates a significant difference (*P* ≤ 0.05).

**Supplementary Table S6**. Pairwise Fst Tukey’s test *P* values. Green indicates a significant difference (*P* ≤ 0.05).

**Supplementary Table S7**. Pairwise SNP per kilobase Tukey’s test *P* values. Green indicates a significant difference (*P* ≤ 0.05).

## Abbreviations

BUSCO: Benchmarking Universal Single-Copy Orthologs; COG: Clusters of Orthologous Groups; GO: Gene Ontology; HMM: hidden Markov model; KEGG: Kyoto Encyclopedia of Genes and Genomes; LAI: long terminal repeat assembly index; MAPQ: mapping quality; NCBI: National Center for Biotechnology Information; PAV: presence/absence variant; PCA: principal component analysis; SD: structural divergence; SNP: single nucleotide polymorphism; SP: structural polymorphism; SSP: shared structural polymorphism; SV: structural variation; TE: transposable element.

## Supplementary Material

giae029_Supplemental_File

giae029_GIGA-D-23-00337_Original_Submission

giae029_GIGA-D-23-00337_Revision_1

giae029_Response_to_Reviewer_Comments_Original_Submission

giae029_Reviewer_1_Report_Original_Submission Jakob Butler -- 12/3/2023

giae029_Reviewer_2_Report_Original_Submission Lejun Ouyang -- 1/27/2024

giae029_Reviewer_3_Report_Original_Submission Danqing Li -- 2/15/2024

## Data Availability

Sequencing data and reference genomes generated in this project are publicly available on the Sequence Read Archive (SRA) and NCBI genome repository under BioProject PRJNA509734 and PRJNA578806. Gene predictions, repeat annotations, SNP vcf, eggNog annotations, PCA data, recombination rate estimates (ρ), fixation index (F_ST_), BUSCO results, samples metadata, and SyRI output have been deposited in FigShare [106]. All analysis scripts created and used by this project have been deposited within the GitHub repository [107]. All additional supporting data are available in the *GigaScience* repository, GigaDB [108].

## References

[1] Alonge M, Wang X, Benoit M, et al. Major impacts of widespread structural variation on gene expression and crop improvement in tomato. Cell. 2020;182:145–61.e23. 10.1016/j.cell.2020.05.021. 32553272 PMC7354227

[2] Imprialou M, Kahles A, Steffen JG, et al. Genomic rearrangements in Arabidopsis considered as quantitative traits. Genetics. 2017;205:1425–41. 10.1534/genetics.116.192823. 28179367 PMC5378104

[3] Weischenfeldt J, Symmons O, Spitz F, et al. Phenotypic impact of genomic structural variation: insights from and for human disease. Nat Rev Genet. 2013;14:125–38. 10.1038/nrg3373. 23329113

[4] Marques DA, Meier JI, Seehausen O. A combinatorial view on speciation and adaptive radiation. Trends Ecol Evol. 2019;34:531–44. 10.1016/j.tree.2019.02.008. 30885412

[5] Zhang L, Reifová R, Halenková Z, et al. How important are structural variants for speciation?. Genes. 2021;12:1084. 10.3390/genes12071084. 34356100 PMC8305853

[6] Savocco J, Piazza A. Recombination-mediated genome rearrangements. Curr Opin Genet Dev. 2021;71:92021. 10.1016/j.gde.2021.06.008 34325160

[7] Sedlazeck FJ, Rescheneder P, Smolka M, et al. Accurate detection of complex structural variations using single-molecule sequencing. Nat Methods. 2018;15:461–68. 10.1038/s41592-018-0001-7. 29713083 PMC5990442

[8] Pokrovac I, Pezer Ž. Recent advances and current challenges in population genomics of structural variation in animals and plants. Front Genet. 2022;13:1060898. 10.3389/fgene.2022.1060898. 36523759 PMC9745067

[9] Marx V . Method of the year: long-read sequencing. Nat Methods. 2023;20:6–11. 10.1038/s41592-022-01730-w. 36635542

[10] Kovaka S, Ou S, Jenike KM, et al. Approaching complete genomes, transcriptomes and epi-omes with accurate long-read sequencing. Nat Methods. 2023;20:12–16. 10.1038/s41592-022-01716-8. 36635537 PMC10068675

[11] Radke DW, Lee C. Adaptive potential of genomic structural variation in human and mammalian evolution. Brief Funct Genomics. 2015;14:358–68. 10.1093/bfgp/elv019. 26003631 PMC6278953

[12] Stewart NB, Rogers RL. Chromosomal rearrangements as a source of new gene formation in Drosophila yakuba. PLoS Genet. 2019;15:e1008314. 10.1371/journal.pgen.1008314. 31545792 PMC6776367

[13] Kim K, Eom J, Jung I. Characterization of structural variations in the context of 3D chromatin structure. Mol Cells. 2019;42:512–22. 10.14348/molcells.2019.0137. 31362468 PMC6681866

[14] Shanta O, Noor A, Chaisson MJP, et al. The effects of common structural variants on 3D chromatin structure. BMC Genomics. 2020;21:95. 10.1186/s12864-020-6516-1. 32000688 PMC6990566

[15] Thompson MJ, Jiggins CD. Supergenes and their role in evolution. Heredity. 2014;113:1–8. 10.1038/hdy.2014.20. 24642887 PMC4815649

[16] Kirkpatrick M, Barton N. Chromosome inversions, local adaptation and speciation. Genetics. 2006;173:419–34. 10.1534/genetics.105.047985. 16204214 PMC1461441

[17] Lande R . The fixation of chromosomal rearrangements in a subdivided population with local extinction and colonization. Heredity. 1985;54:323–32. 10.1038/hdy.1985.43. 4019220

[18] Walsh JB . Rate of accumulation of reproductive isolation by chromosome rearrangements. Am Nat. 1982;120:510–32. 10.1086/284008.

[19] Rieseberg LH . Chromosomal rearrangements and speciation. Trends Ecol Evol. 2001;16(7):351–58. 10.1016/S0169-5347(01)02187-5. 11403867

[20] Harringmeyer OS, Hoekstra HE. Chromosomal inversion polymorphisms shape the genomic landscape of deer mice. Nat Ecol Evol. 2022;6:1965–79. 10.1038/s41559-022-01890-0. 36253543 PMC9715431

[21] Robberecht C, Voet T, Esteki MZ, et al. Nonallelic homologous recombination between retrotransposable elements is a driver of de novo unbalanced translocations. Genome Res. 2013;23:411–18. 10.1101/gr.145631.112. 23212949 PMC3589530

[22] Ortiz-Barrientos D, Engelstädter J, Rieseberg LH. Recombination rate evolution and the origin of species. Trends Ecol Evol. 2016;31:226–36. 10.1016/j.tree.2015.12.016. 26831635

[23] Flagel LE, Wendel JF. Gene duplication and evolutionary novelty in plants. New Phytol. 2009;183:557–64. 10.1111/j.1469-8137.2009.02923.x. 19555435

[24] Wu B, Cox MP. Greater genetic and regulatory plasticity of retained duplicates in Epichloë endophytic fungi. Mol Ecol. 2019;28:5103–14. 10.1111/mec.15275. 31614039 PMC7004115

[25] Braasch I, Gehrke AR, Smith JJ, et al. The spotted gar genome illuminates vertebrate evolution and facilitates human-teleost comparisons. Nat Genet. 2016;48:427–37. 10.1038/ng.3526. 26950095 PMC4817229

[26] Freeling M, Scanlon MJ, Fowler JE. Fractionation and subfunctionalization following genome duplications: mechanisms that drive gene content and their consequences. Curr Opin Genet Dev. 2015;35:110–18. 10.1016/j.gde.2015.11.002. 26657818

[27] Lien S, Koop BF, Sandve SR, et al. The Atlantic salmon genome provides insights into rediploidization. Nature. 2016;533:200–5. 10.1038/nature17164. 27088604 PMC8127823

[28] Conrad DF, Hurles ME. The population genetics of structural variation. Nat Genet. 2007;39:S30–36. 10.1038/ng2042. 17597779 PMC2716079

[29] Sun Y, Wang J, Li Y, et al. Pan-genome analysis reveals the abundant gene presence/absence variations among different varieties of melon and their influence on traits. Front Plant Sci. 2022;13. 10.3389/fpls.2022.835496. PMC8990847 35401600

[30] Yuan Y, Bayer PE, Batley J, et al. Current status of structural variation studies in plants. Plant Biotechnol J. 2021;19:2153–63. 10.1111/pbi.13646. 34101329 PMC8541774

[31] Ferguson S, Jones A, Murray K, et al. Interspecies genome divergence is predominantly due to frequent small scale rearrangements in Eucalyptus. Mol Ecol. 2023;32:1271–87. 10.1111/mec.16608. 35810343

[32] Hejase HA, Salman-Minkov A, Campagna L, et al. Genomic islands of differentiation in a rapid avian radiation have been driven by recent selective sweeps. Proc Natl Acad Sci USA. 2020;117:30554–65. 10.1073/pnas.2015987117. 33199636 PMC7720181

[33] Eshel G, Araus V, Undurraga S, et al. Plant ecological genomics at the limits of life in the Atacama Desert. Proc Natl Acad Sci USA. 2021;118:e2101177118. 10.1073/pnas.2101177118. 34725254 PMC8609638

[34] Henderson EC, Brelsford A. Genomic differentiation across the speciation continuum in three hummingbird species pairs. BMC Evol Biol. 2020;20:113. 10.1186/s12862-020-01674-9. 32883209 PMC7469328

[35] Piatkowski B, Weston DJ, Aguero B, et al. Divergent selection and climate adaptation fuel genomic differentiation between sister species of Sphagnum (peat moss). Ann Bot. 2023;132:499–512. 10.1093/aob/mcad104. 37478307 PMC10666999

[36] Zhang J, Zhang S, Zheng Z, et al. Genomic divergence between two sister Ostrya species through linked selection and recombination. Ecol Evol. 2022;12:e9611. 10.1002/ece3.9611. 36540075 PMC9754895

[37] Ferguson S, Jones A, Murray K, et al. Plant genome evolution in the genus Eucalyptus driven by structural rearrangements that promote sequence divergence. Genome Research. 2024;34:606–19. 10.1101/gr.277999.123. 38589251 PMC11146599

[38] Thornhill AH, Crisp MD, Külheim C, et al. A dated molecular perspective of eucalypt taxonomy, evolution and diversification. Aust Syst Bot. 2019;32:29–48. 10.1071/SB18015.

[39] Alser M, Rotman J, Deshpande D, et al. Technology dictates algorithms: recent developments in read alignment. Genome Biol. 2021;22:249. 10.1186/s13059-021-02443-7. 34446078 PMC8390189

[40] Valiente-Mullor C, Beamud B, Ansari I, et al. One is not enough: on the effects of reference genome for the mapping and subsequent analyses of short-reads. PLoS Comput Biol. 2021;17:e1008678. 10.1371/journal.pcbi.1008678. 33503026 PMC7870062

[41] Galperin MY, Wolf YI, Makarova KS, et al. COG database update: focus on microbial diversity, model organisms, and widespread pathogens. Nucleic Acids Res. 2021;49:D274–81. 10.1093/nar/gkaa1018. 33167031 PMC7778934

[42] Gene Ontology Consortium , AleksanderSA, Balhoff J, et al. The Gene Ontology knowledgebase in 2023. Genetics. 2023;224:iyad031. 10.1093/genetics/iyad031. 36866529 PMC10158837

[43] Akey JM, Zhang G, Zhang K, et al. Interrogating a high-density SNP map for signatures of natural selection. Genome Res. 2002;12:1805–14. 10.1101/gr.631202. 12466284 PMC187574

[44] Bhatia G, Patterson N, Sankararaman S, et al. Estimating and interpreting FST: the impact of rare variants. Genome Res. 2013;23:1514–21. 10.1101/gr.154831.113. 23861382 PMC3759727

[45] Chan AH, Jenkins PA, Song YS. Genome-wide fine-scale recombination rate variation in Drosophila melanogaster. PLoS Genet. 2012;8:e1003090. 10.1371/journal.pgen.1003090. 23284288 PMC3527307

[46] Escaramís G, Docampo E, Rabionet R. A decade of structural variants: description, history and methods to detect structural variation. Brief Funct Genomics. 2015;14:305–14. 10.1093/bfgp/elv014. 25877305

[47] Chain FJJ, Feulner PGD. Ecological and evolutionary implications of genomic structural variations. Front Genet. 2014;5:326. 10.3389/fgene.2014.00326. 25278961 PMC4165313

[48] Ho SS, Urban AE, Mills RE. Structural variation in the sequencing era. Nat Rev Genet. 2020;21:171–89. 10.1038/s41576-019-0180-9. 31729472 PMC7402362

[49] Yan SM, Sherman RM, Taylor DJ, et al. Local adaptation and archaic introgression shape global diversity at human structural variant loci. eLife. 2021;10:e67615. 10.7554/eLife.67615. 34528508 PMC8492059

[50] Gui S, Wei W, Jiang C, et al. A pan-Zea genome map for enhancing maize improvement. Genome Biol. 2022;23:178. 10.1186/s13059-022-02742-7. 35999561 PMC9396798

[51] Hufford MB, Seetharam AS, Woodhouse MR, et al. De novo assembly, annotation, and comparative analysis of 26 diverse maize genomes. Science. 2021;373(6555):655–62. 10.1126/science.abg5289. 34353948 PMC8733867

[52] Ishikawa A, Kabeya N, Ikeya K, et al. A key metabolic gene for recurrent freshwater colonization and radiation in fishes. Science. 2019;364:886–89. 10.1126/science.aau5656. 31147520

[53] Zhao Y, Long L, Wan J, et al. A spontaneous complex structural variant in rcan-1 increases exploratory behavior and laboratory fitness of Caenorhabditis elegans. PLoS Genet. 2020;16:e1008606. 10.1371/journal.pgen.1008606. 32092052 PMC7058356

[54] Shang H, Hess J, Pickup M, et al. Evolution of strong reproductive isolation in plants: broad-scale patterns and lessons from a perennial model group. Phil Trans R Soc B. 2020;375:20190544. 10.1098/rstb.2019.0544. 32654641 PMC7423283

[55] Ravinet M, Faria R, Butlin RK, et al. Interpreting the genomic landscape of speciation: a road map for finding barriers to gene flow. J Evol Biol. 2017;30:1450–77. 10.1111/jeb.13047. 28786193

[56] Christie K, Fraser LS, Lowry DB. The strength of reproductive isolating barriers in seed plants: insights from studies quantifying premating and postmating reproductive barriers over the past 15 years. Evolution. 2022;76:2228–43. 10.1111/evo.14565. 35838076 PMC9796645

[57] Berg PR, Star B, Pampoulie C, et al. Three chromosomal rearrangements promote genomic divergence between migratory and stationary ecotypes of Atlantic cod. Sci Rep. 2016;6:23246. 10.1038/srep23246. 26983361 PMC4794648

[58] Huang K, Andrew RL, Owens GL, et al. Multiple chromosomal inversions contribute to adaptive divergence of a dune sunflower ecotype. Mol Ecol. 2020;29:2535–49. 10.1111/mec.15428. 32246540

[59] Lucek K, Gompert Z, Nosil P. The role of structural genomic variants in population differentiation and ecotype formation in *Timema cristinae* walking sticks. Mol Ecol. 2019;28:1224–37. 10.1111/mec.15016. 30636326

[60] Gregory TR . Understanding natural selection: essential concepts and common misconceptions. Evo Edu Outreach. 2009;2:156–75. 10.1007/s12052-009-0128-1.

[61] Loewe L, Hill WG. The population genetics of mutations: good, bad and indifferent. Phil Trans R Soc B. 2010;365:1153–67. 10.1098/rstb.2009.0317. 20308090 PMC2871823

[62] De Oliveira R, Rimbert H, Balfourier F, et al. Structural variations affecting genes and transposable elements of chromosome 3B in wheats. Front Genet. 2020;11:112020. 10.3389/fgene.2020.00891. PMC7461782 33014014

[63] Mérot C, Oomen RA, Tigano A, et al. A roadmap for understanding the evolutionary significance of structural genomic variation. Trends Ecol Evol. 2020;35:561–72. 10.1016/j.tree.2020.03.002. 32521241

[64] Wellenreuther M, Mérot C, Berdan E, et al. Going beyond SNPs: the role of structural genomic variants in adaptive evolution and species diversification. Mol Ecol. 2019;28:1203–9. 10.1111/mec.15066. 30834648

[65] Cohen ZP, Schoville SD, Hawthorne DJ. The role of structural variants in pest adaptation and genome evolution of the Colorado potato beetle, *Leptinotarsa decemlineata* (Say). Mol Ecol. 2023;32:1425–40. 10.1111/mec.16838. 36591939

[66] Hanada K, Zou C, Lehti-Shiu MD, et al. Importance of lineage-specific expansion of plant tandem duplicates in the adaptive response to environmental stimuli. Plant Physiol. 2008;148:993–1003. 10.1104/pp.108.122457. 18715958 PMC2556807

[67] Inoue J, Sato Y, Sinclair R, et al. Rapid genome reshaping by multiple-gene loss after whole-genome duplication in teleost fish suggested by mathematical modeling. Proc Natl Acad Sci USA. 2015;112:14918–23. 10.1073/pnas.1507669112. 26578810 PMC4672829

[68] Naseeb S, Ames RM, Delneri D, et al. Rapid functional and evolutionary changes follow gene duplication in yeast. Proc Biol Sci. 2017;284:20171393. 10.1098/rspb.2017.1393. 28835561 PMC5577496

[69] Ferguson S, McLay T, Andrew RL, et al. Species-specific basecallers improve actual accuracy of nanopore sequencing in plants. Plant Methods. 2022;18:137. 10.1186/s13007-022-00971-2. 36517904 PMC9749173

[70] Alwadani KG, Janes JK, Andrew RL. Chloroplast genome analysis of box-ironbark Eucalyptus. Mol Phylogenet Evol. 2019;136:76–86. 10.1016/j.ympev.2019.04.001. 30954587

[71] Murray KD, Janes JK, Jones A, et al. Landscape drivers of genomic diversity and divergence in woodland Eucalyptus. Mol Ecol. 2019;28:5232–47. 10.1111/mec.15287. 31647597 PMC7065176

[72] Supple MA, Bragg JG, Broadhurst LM, et al. Landscape genomic prediction for restoration of a *Eucalyptus* foundation species under climate change. eLife. 2018;7:e31835. 10.7554/eLife.31835. 29685183 PMC5951681

[73] Jones A, Stanley D, Ferguson S, et al. Cost-conscious generation of multiplexed short-read DNA libraries for whole-genome sequencing. PLoS One. 2023;18:e0280004. 10.1371/journal.pone.0280004. 36706059 PMC9882895

[74] Jones A, Torkel C, Stanley D, et al. High-molecular weight DNA extraction, clean-up and size selection for long-read sequencing. PLoS One. 2021;16:e0253830. 10.1371/journal.pone.0253830. 34264958 PMC8282028

[75] Ferguson S, Jones A, Borevitz J. Plant assemble—plant de novo genome assembly, scaffolding and annotation for genomic studies. protocols.io. 2022. 10.17504/protocols.io.81wgb6zk3lpk/v1. Accessed 4 August 2022.

[76] Li H . Aligning sequence reads, clone sequences and assembly contigs with BWA-MEM. ArXiv13033997 Q-Bio. 2013.; http://arxiv.org/abs/1303.3997

[77] Faust GG, Hall IM. SAMBLASTER: fast duplicate marking and structural variant read extraction. Bioinformatics. 2014;30:2503–5. 10.1093/bioinformatics/btu314. 24812344 PMC4147885

[78] Durand NC, Shamim MS, Machol I, et al. Juicer provides a one-click System for analyzing loop-resolution Hi-C experiments. Cell Syst. 2016;3:95–98. 10.1016/j.cels.2016.07.002. 27467249 PMC5846465

[79] Dudchenko O, Batra SS, Omer AD, et al. De novo assembly of the *Aedes aegypti* genome using Hi-C yields chromosome-length scaffolds. Science. 2017;356:92–95. 10.1126/science.aal3327. 28336562 PMC5635820

[80] Durand NC, Robinson JT, Shamim MS, et al. Juicebox provides a visualization system for Hi-C contact maps with unlimited zoom. Cell Syst. 2016;3:99–101. 10.1016/j.cels.2015.07.012. 27467250 PMC5596920

[81] Alonge M, Lebeigle L, Kirsche M, et al. Automated assembly scaffolding using RagTag elevates a new tomato system for high-throughput genome editing. Genome Biol. 2022;23:258. 10.1186/s13059-022-02823-7. 36522651 PMC9753292

[82] Manni M, Berkeley MR, Seppey M, et al. BUSCO update: novel and streamlined workflows along with broader and deeper phylogenetic coverage for scoring of eukaryotic, prokaryotic, and viral genomes. Mol Biol Evol. 2021;38:4647–54. 10.1093/molbev/msab199. 34320186 PMC8476166

[83] Ou S, Chen J, Jiang N. Assessing genome assembly quality using the LTR Assembly Index (LAI). Nucleic Acids Res. 2018;46:e126. 10.1093/nar/gky730. 30107434 PMC6265445

[84] Ou S, Su W, Liao Y, et al. Benchmarking transposable element annotation methods for creation of a streamlined, comprehensive pipeline. Genome Biol. 2019;20:275. 10.1186/s13059-019-1905-y. 31843001 PMC6913007

[85] Smit A, Hubley R, Green P. RepeatMasker Open-4.0. 2020. http://www.repeatmasker.org. Accessed 11 February 2020.

[86] Brůna T, Hoff KJ, Lomsadze A, et al. BRAKER2: automatic eukaryotic genome annotation with GeneMark-EP+ and AUGUSTUS supported by a protein database. NAR Genomics Bioinforma. 2021;3:lqaa108. 10.1093/nargab/lqaa108. PMC7787252 33575650

[87] Brůna T, Lomsadze A, Borodovsky M. GeneMark-EP+: eukaryotic gene prediction with self-training in the space of genes and proteins. NAR Genomics Bioinforma. 2020;2:lqaa026. 10.1093/nargab/lqaa026. PMC7222226 32440658

[88] Sayers EW, Beck J, Bolton EE, et al. Database resources of the National Center for Biotechnology Information. Nucleic Acids Res. 2021;49:D10–17. 10.1093/nar/gkaa892. 33095870 PMC7778943

[89] Cantalapiedra CP, Hernández-Plaza A, Letunic I, et al. eggNOG-mapper v2: functional annotation, orthology assignments, and domain prediction at the metagenomic scale. Mol Biol Evol. 2021;38:5825–29. 10.1093/molbev/msab293. 34597405 PMC8662613

[90] Klopfenstein DV, Zhang L, Pedersen BS, et al. GOATOOLS: a Python library for Gene Ontology analyses. Sci Rep. 2018;8:10872. 10.1038/s41598-018-28948-z. 30022098 PMC6052049

[91] Kurtz S, Phillippy A, Delcher AL, et al. Versatile and open software for comparing large genomes. Genome Biol. 2004;5(2):R12. 10.1186/gb-2004-5-2-r12. 14759262 PMC395750

[92] Goel M, Sun H, Jiao W-B, et al. SyRI: finding genomic rearrangements and local sequence differences from whole-genome assemblies. Genome Biol. 2019;20:277. 10.1186/s13059-019-1911-0. 31842948 PMC6913012

[93] Goel M, Schneeberger K. plotsr: visualizing structural similarities and rearrangements between multiple genomes. Bioinformatics. 2022;38:2922–26. 10.1093/bioinformatics/btac196. 35561173 PMC9113368

[94] Chen S, Krusche P, Dolzhenko E, et al. Paragraph: a graph-based structural variant genotyper for short-read sequence data. Genome Biol. 2019;20:291. 10.1186/s13059-019-1909-7. 31856913 PMC6921448

[95] R Core Team . R: A Language and Environment for Statistical Computing. Vienna, Austria: R Foundation for Statistical Computing, 2023. https://www.r-project.org/.

[96] Schubert M, Lindgreen S, Orlando L. AdapterRemoval v2: rapid adapter trimming, identification, and read merging. BMC Res Notes. 2016;9:88. 10.1186/s13104-016-1900-2. 26868221 PMC4751634

[97] Danecek P, Bonfield JK, Liddle J, et al. Twelve years of SAMtools and BCFtools. Gigascience. 2021;10: giab008. 10.1093/gigascience/giab008. 33590861 PMC7931819

[98] Murray K . kdm9/Acanthophis: version 0.2.0. Zenodo. 2023. Published: 7 Oct 2023. 10.5281/zenodo.8416057.

[99] Meisner J, Albrechtsen A. Inferring population structure and admixture proportions in low-depth NGS data. Genetics. 2018;210:719–31. 10.1534/genetics.118.301336. 30131346 PMC6216594

[100] Martin M, Patterson M, Garg S, et al. WhatsHap: fast and accurate read-based phasing. Biorxiv. 2016. 10.1101/085050.

[101] Delaneau O, Zagury J-F, Robinson MR, et al. Accurate, scalable and integrative haplotype estimation. Nat Commun. 2019;10:5436. 10.1038/s41467-019-13225-y. 31780650 PMC6882857

[102] Hermann P, Heissl A, Tiemann-Boege I, et al. Estimating variable recombination rates from population genetic data. Mol Ecol Resour. 2019;19:623–38. 10.1111/1755-0998.12994. 30666785 PMC6519033

[103] Auton A, McVean G. Recombination rate estimation in the presence of hotspots. Genome Res. 2007;17:1219–27. 10.1101/gr.6386707. 17623807 PMC1933511

[104] Kitada S, Nakamichi R, Kishino H. Understanding population structure in an evolutionary context: population-specific *F* ST and pairwise *F* ST. G3 (Bethesda). 2021;11:jkab316. 10.1093/g3journal/jkab316. 34549777 PMC8527463

[105] Chang CC, Chow CC, Tellier LC, et al. Second-generation PLINK: rising to the challenge of larger and richer datasets. Gigascience. 2015;4(1):s13742–015-0047-8. 10.1186/s13742-015-0047-8. PMC4342193 25722852

[106] Ferguson S . Exploring polymorphic interspecies structural variants in Eucalyptus: unravelling their role in reproductive isolation and adaptive divergence. Figshare. 2023. https://figshare.com/projects/Exploring_polymorphic_interspecies_structural_variants_in_Eucalyptus_Unravelling_Their_Role_in_Reproductive_Isolation_and_Adaptive_Divergence_/183577. Published: 20 Oct 2023. 10.1093/gigascience/giae029 PMC11170218 38869149

[107] GitHub repository. https://github.com/fergsc/Polymorphic-interspecies-SVs.

[108] Ferguson S, Jones A, Murray K, et al. Supporting data for “Exploring Polymorphic Interspecies Structural Variants in Eucalyptus: Unravelling Their Role in Reproductive Isolation and Adaptive Divergence.”. GigaScience Database. 2024. 10.5524/102519. PMC11170218 38869149

