# Supplementary material for: Exploring the role of polymorphic interspecies structural variants in reproductive isolation and adaptive divergence in Eucalyptus
Source: Gigascience. 2024 Jun 13;13:giae029. doi: 10.1093/gigascience/giae029 (PMC11170218; doi:10.1093/gigascience/giae029)
Supplement: giae029_Supplemental_File [file giae029_supplemental_file.pdf]

	MAPQ >= 30	MAPQ > 0
Sequenced Read Pairs	151,590,503	151,590,503
Normal Paired	45,342,881 (29.91%)	45,342,881 (29.91%)
Chimeric Paired	52,358,576 (34.54%)	52,358,576 (34.54%)
Chimeric Ambiguous	25,426,118 (16.77%)	25,426,118 (16.77%)
Unmapped	28,462,928 (18.78%)	28,462,928 (18.78%)
Ligation Motif Present	0 (0.00%)	0 (0.00%)
Alignable (Normal+Chimeric Paired)	97,701,457 (64.45%)	97,701,457 (64.45%)
Unique Reads	40,318,503 (26.60%)	40,318,503 (26.60%)
PCR Duplicates	54,402,352 (35.89%)	54,402,352 (35.89%)
Optical Duplicates	2,980,602 (1.97%)	2,980,602 (1.97%)
Library Complexity Estimate	46,306,556	46,306,556
Intra-fragment Reads	0 (0.00% / 0.00%)	0 (0.00% / 0.00%)
Below MAPQ Threshold	21,810,955 (14.39%)	14,070,582 (9.28%)
Hi-C Contacts	18,507,548 (12.21%)	26,247,921 (17.32%)
Ligation Motif Present	0 (0.00% / 0.00%)	0 (0.00% / 0.00%)
3' Bias (Long Range)	50% - 50%	50% - 50%
Pair Type %(L-I-O-R)	25% - 25% - 25% - 25%	24% - 26% - 26% - 24%
Inter-chromosomal	9,612,532 (6.34%)	14,833,362 (9.79%)
Intra-chromosomal	8,895,016 (5.87%)	11,414,559 (7.53%)
Short Range (<20Kb)	6,771,738 (4.47%)	8,386,180 (5.53%)
Long Range (>20Kb)	2,113,340 (1.39%)	3,016,215 (1.99%)

Supplementary Table S1. *E. melliodora* Hi-C summary stats, produced by Juicer.

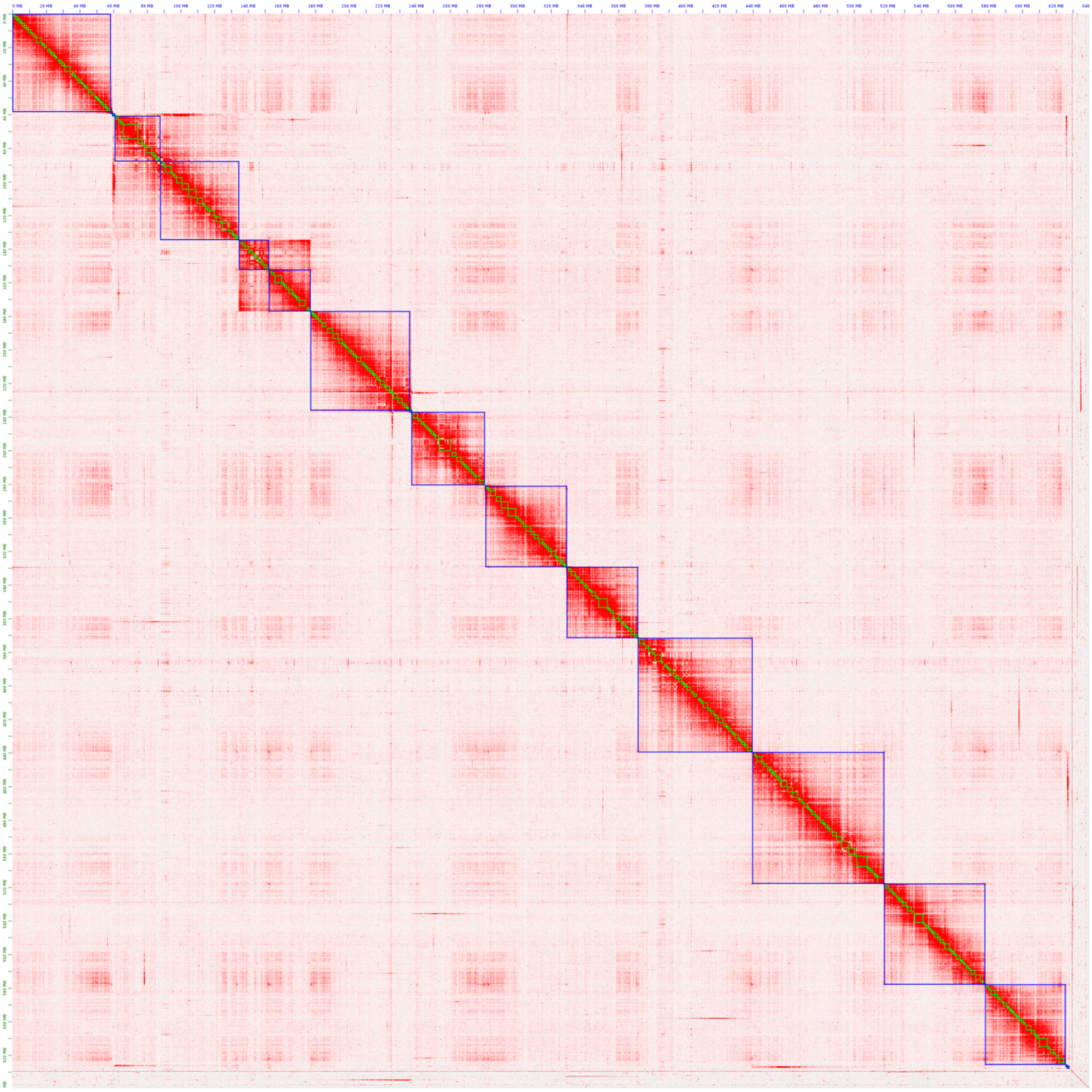

Supplementary Figure S1. Hi-C scaffolding of *E. melliodora's* contigs with 3D DNA (parameter: "--editor-repeat-coverage 5, -i 1000). Due to a high repeat content Hi-C read coverage is highly variable, resulting in poor scaffolding. Hi-C contacts are visualised with Juicebox.

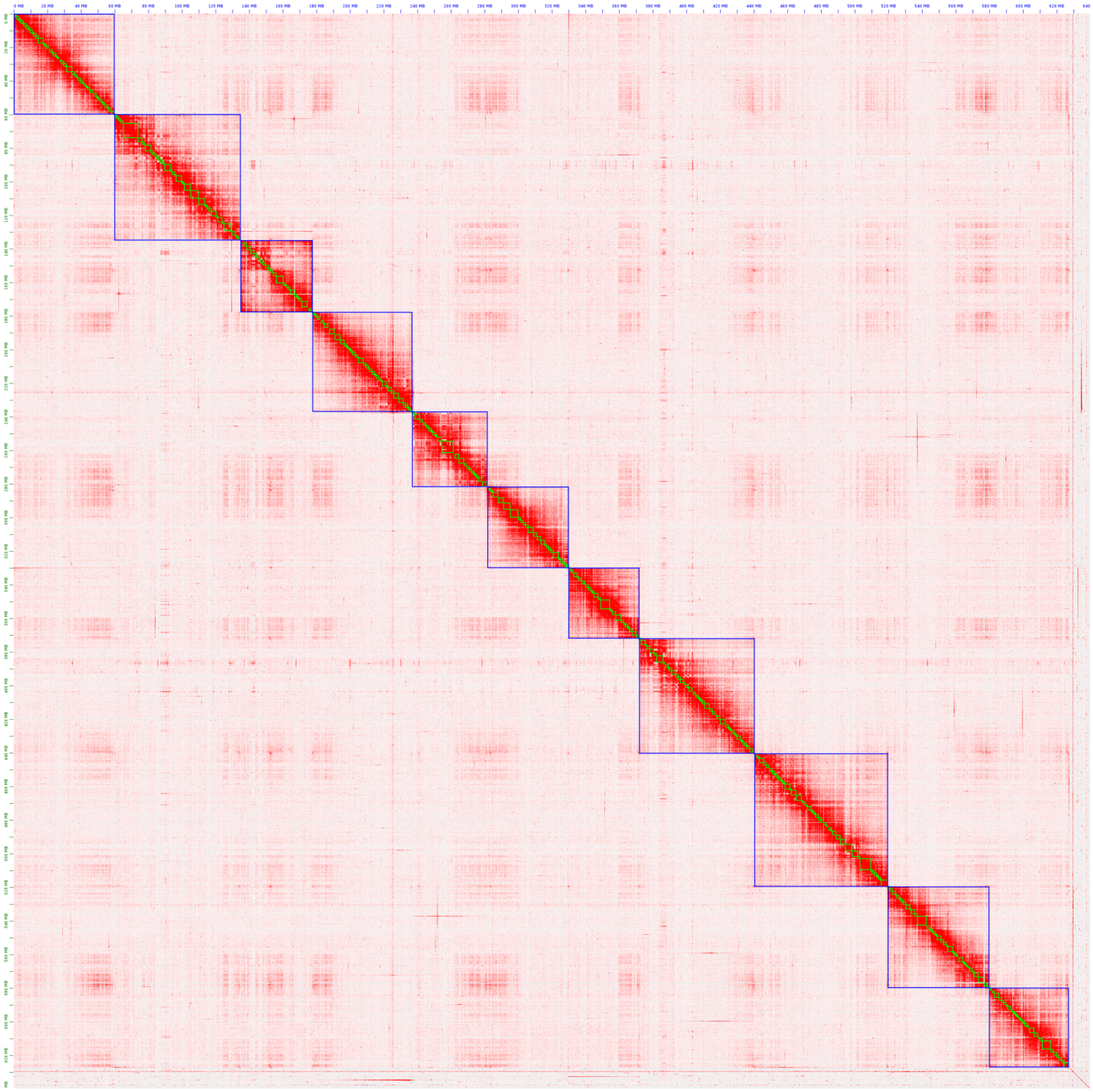

Supplementary Figure S2. Manually curated, final, Hi-C contact map of *E. melliodora's* contigs with 3D DNA (parameter: "--editor-repeat-coverage 5, -i 1000). Due to a high repeat content Hi-C read coverage is highly variable, resulting in poor scaffolding. Hi-C contacts are visualised with Juicebox.

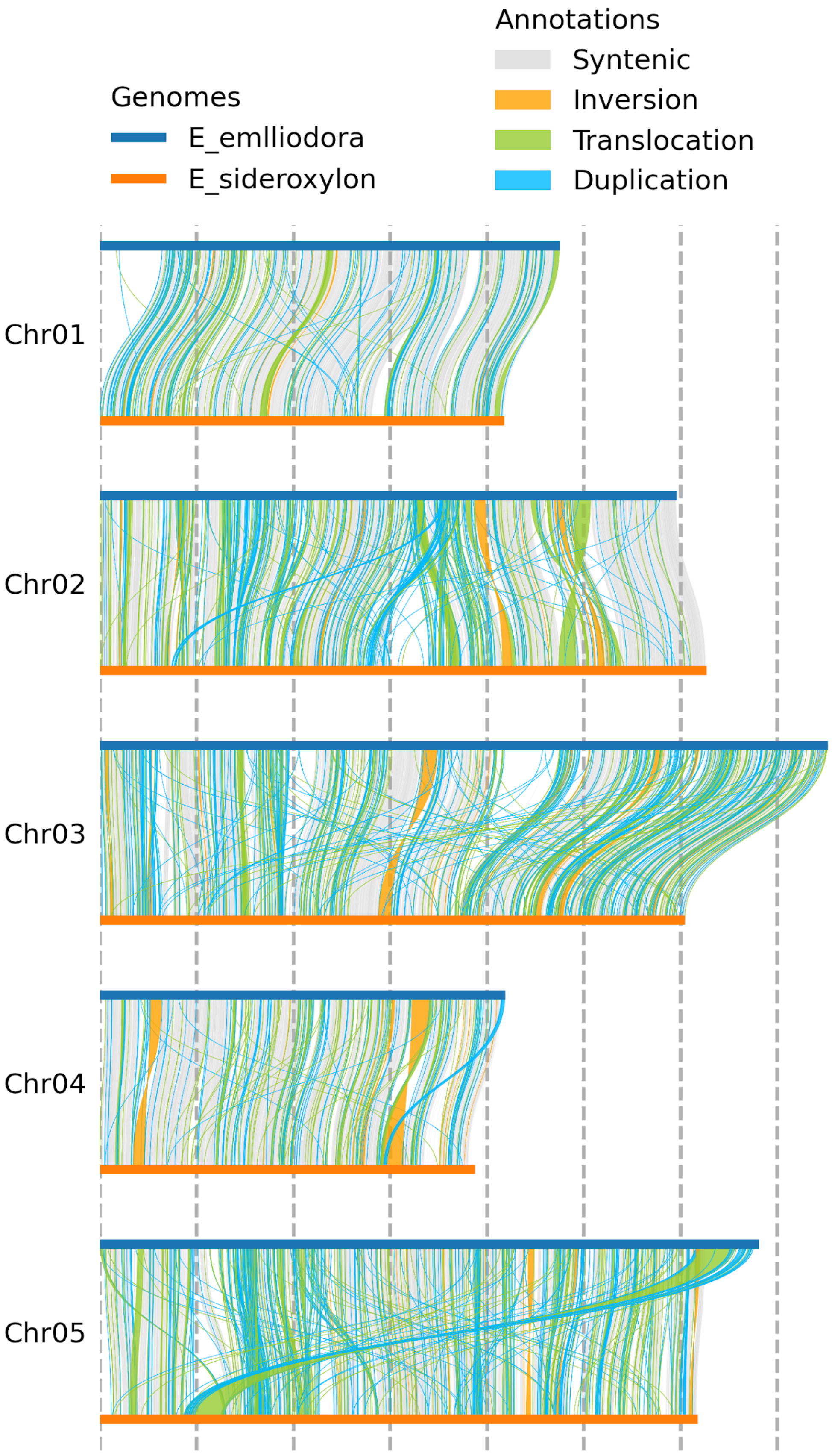

Annotations
Syntenic
Inversion
Translocation
Duplication
Genomes
E_emlliodora
E_sideroxylon
Chr01
Chr02
Chr03
Chr04
Chr05

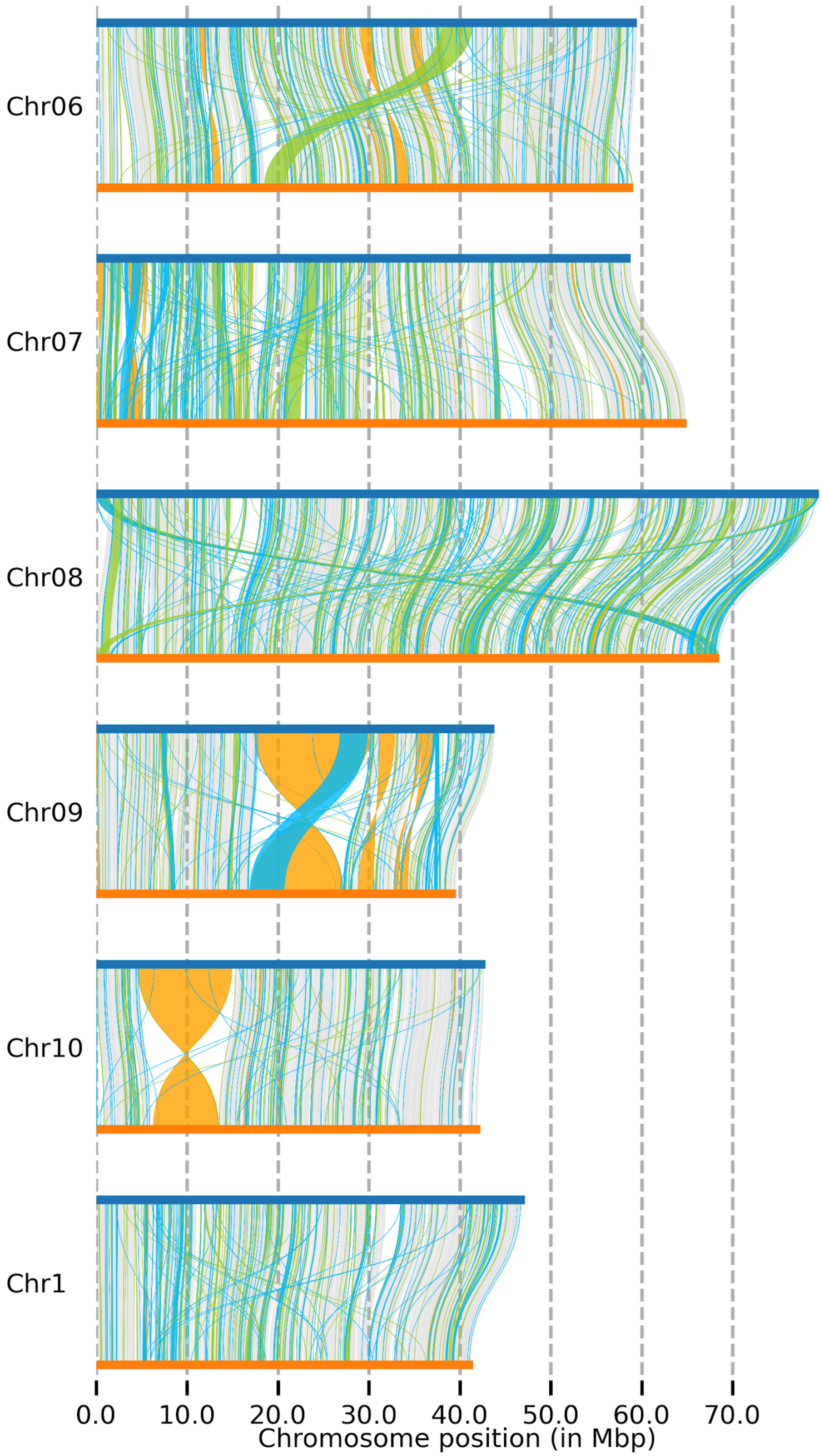

Supplementary Figure S3. SyRI annotations.

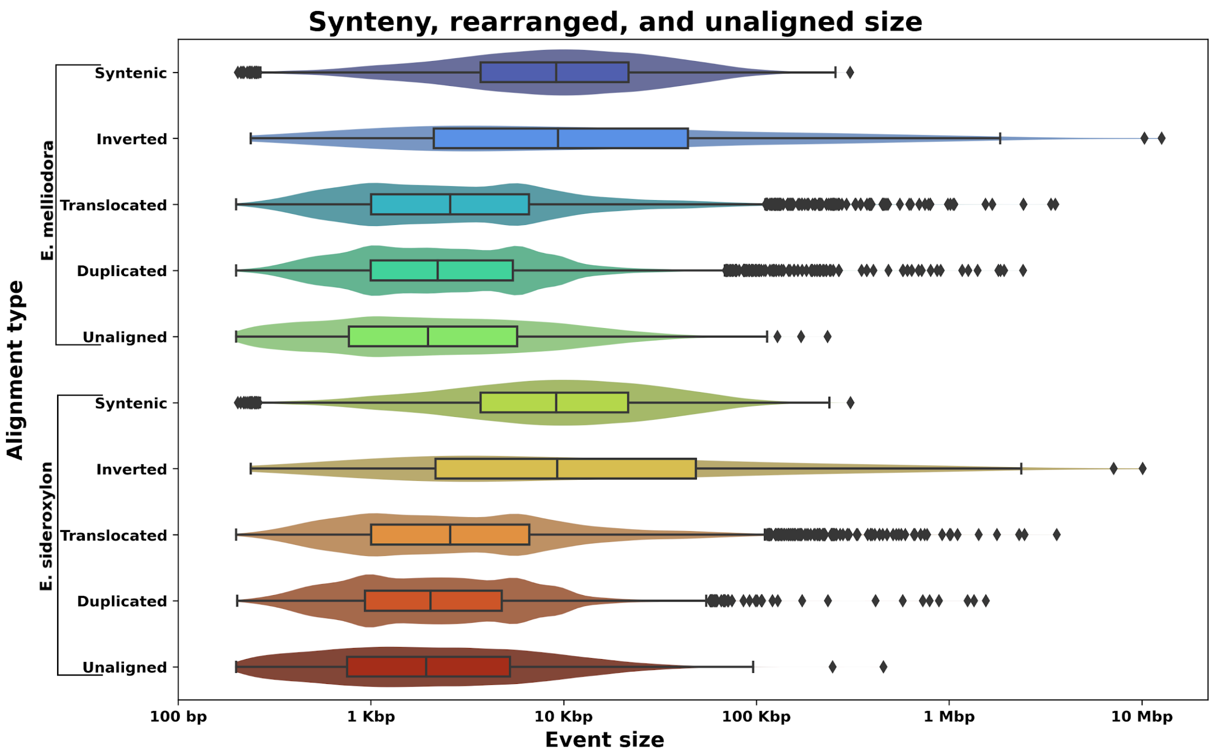

Supplementary Figure S4.

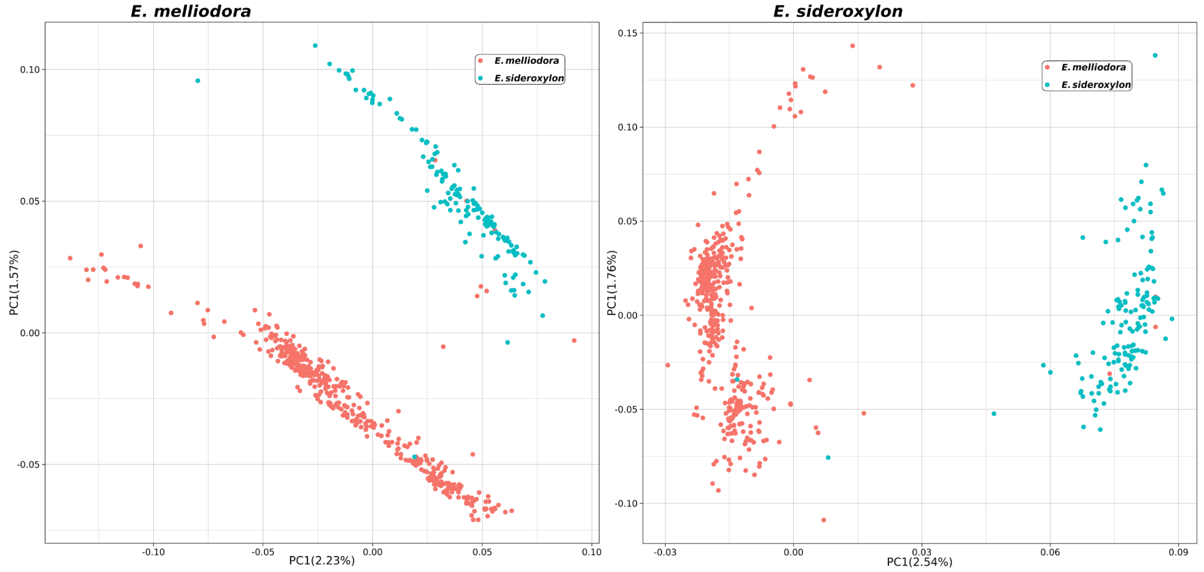

Supplementary Figure S5. Raw PCA plots. Left figure uses *E. melliodora* as the reference, the right figure uses *E. sideroxylon* as the reference.

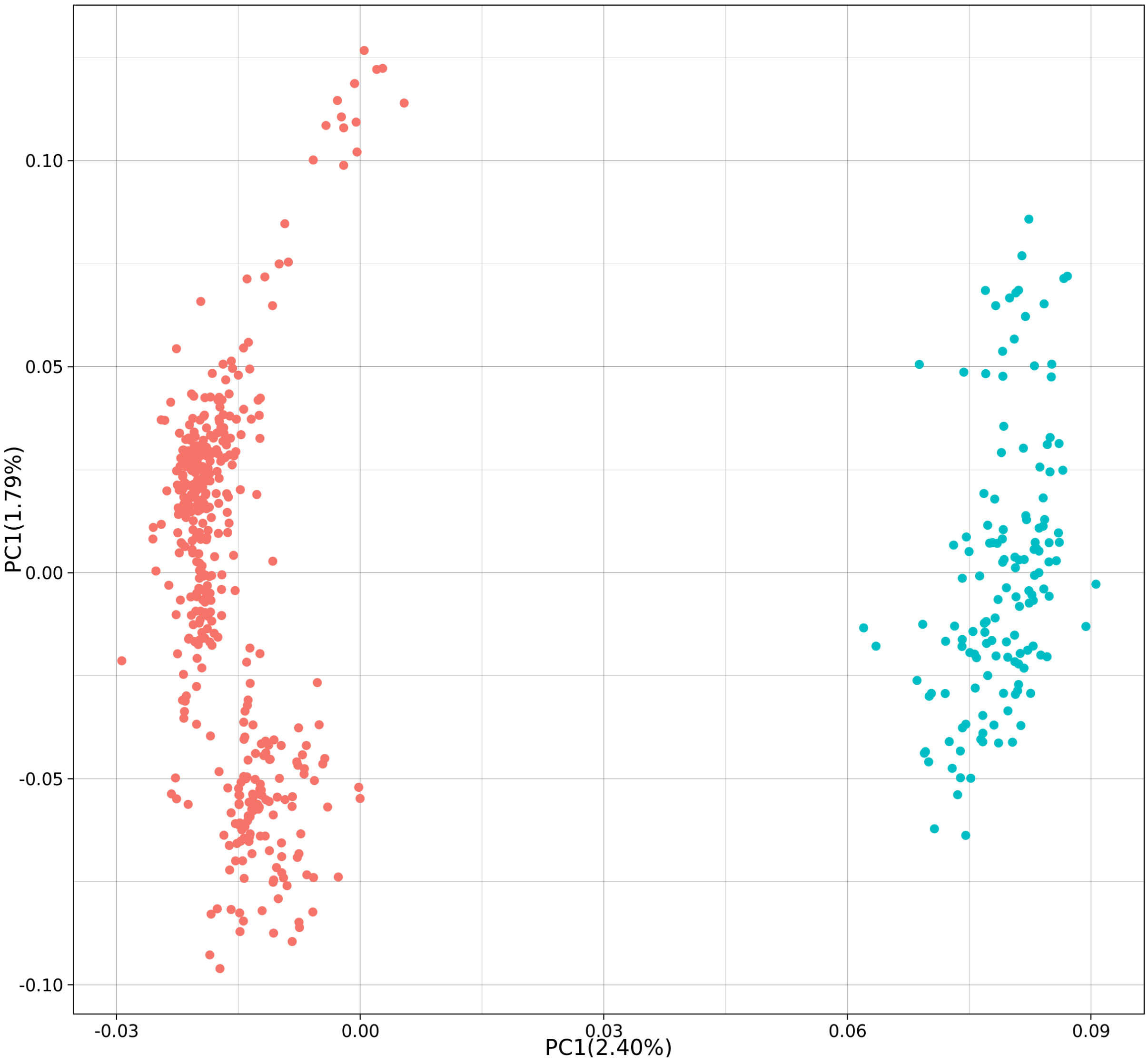

Supplementary Figure S6. Clean PCA plot, *E. Sideroxylon* as reference.

	**Status**				
	E. melliodora	***E. sideroxylon***	**Number of events**	**Proportion of events**	**classification**
**Inversion**	Fixed	Absent	0	0.00%	SD
	Absent	Fixed	0	0.00%	
	Polymorphic	Polymorphic	18	7.79%	SSP
	Fixed	Polymorphic	16	6.93%	SP
	Polymorphic	Fixed	44	19.05%	
	Absent	Polymorphic	0	0.00%	
	Polymorphic	Absent	0	0.00%	
	Absent	Absent	0	0.00%	Other
	Fixed	Fixed	98	42.42%	
	Ungenotyped	Absent/Fixed/Polymorphic/Ungenotyped	55	23.81%	
	Absent/Fixed/Polymorphic/Ungenotyped	Ungenotyped			
**Translocation**	Fixed	Absent	16	0.15%	SD
	Absent	Fixed	18	0.17%	
	Polymorphic	Polymorphic	910	8.81%	SSP
	Fixed	Polymorphic	801	7.75%	SP
	Polymorphic	Fixed	1,665	16.11%	
	Absent	Polymorphic	25	0.24%	
	Polymorphic	Absent	72	0.70%	
	Absent	Absent	10	0.10%	Other
	Fixed	Fixed	4,034	39.04%	
	Ungenotyped	Absent/Fixed/Polymorphic/Ungenotyped	2,781	26.92%	
	Absent/Fixed/Polymorphic/Ungenotyped	Ungenotyped			

Supplementary Table S2.

Species	GO	Category	Enrichment	Name
Both	GO:0003674	Molecular Function	Lower	molecular_function
	GO:0003676	Molecular Function	Lower	nucleic acid binding
	GO:0003824	Molecular Function	Lower	catalytic activity
	GO:0005488	Molecular Function	Lower	binding
	GO:0005575	Cellular Component	Lower	cellular_component
	GO:0005622	Cellular Component	Lower	intracellular anatomical structure
	GO:0005634	Cellular Component	Lower	nucleus
	GO:0005737	Cellular Component	Lower	cytoplasm
	GO:0006139	Biological Process	Lower	nucleobase-containing compound metabolic process
	GO:0006807	Biological Process	Lower	nitrogen compound metabolic process
	GO:0008150	Biological Process	Lower	biological_process
	GO:0008152	Biological Process	Lower	metabolic process
	GO:0009987	Biological Process	Lower	cellular process
	GO:0016020	Cellular Component	Lower	membrane
	GO:0032502	Biological Process	Lower	developmental process
	GO:0036094	Molecular Function	Lower	small molecule binding
	GO:0043170	Biological Process	Lower	macromolecule metabolic process
	GO:0043226	Cellular Component	Lower	organelle
	GO:0043227	Cellular Component	Lower	membrane-bounded organelle
	GO:0043229	Cellular Component	Lower	intracellular organelle
	GO:0043231	Cellular Component	Lower	intracellular membrane-bounded organelle
	GO:0044237	Biological Process	Lower	cellular metabolic process
	GO:0044238	Biological Process	Lower	primary metabolic process
	GO:0050789	Biological Process	Lower	regulation of biological process
	GO:0050794	Biological Process	Lower	regulation of cellular process
	GO:0050896	Biological Process	Lower	response to stimulus
	GO:0065007	Biological Process	Lower	biological regulation
	GO:0071704	Biological Process	Lower	organic substance metabolic process
	GO:0097159	Molecular Function	Lower	organic cyclic compound binding
	GO:0110165	Cellular Component	Lower	cellular anatomical entity
	GO:1901363	Molecular Function	Lower	heterocyclic compound binding
E. melliodora	GO:0042221	Biological Process	Lower	response to chemical
	GO:0016043	Biological Process	Lower	cellular component organisation
	GO:0071840	Biological Process	Lower	cellular component organisation or biogenesis
	GO:0034641	Biological Process	Lower	cellular nitrogen compound metabolic process
	GO:1901564	Biological Process	Lower	organonitrogen compound metabolic process
	GO:1901360	Biological Process	Lower	organic cyclic compound metabolic process
	GO:0048856	Biological Process	Lower	anatomical structure development
	GO:0009058	Biological Process	Lower	biosynthetic process

	GO:0071944	Cellular Component	Lower	cell periphery
	GO:0005829	Cellular Component	Lower	cytosol
	GO:0005886	Cellular Component	Lower	plasma membrane
E. sideroxylon	GO:0090304	Biological Process	Lower	nucleic acid metabolic process
	GO:0060255	Biological Process	Lower	regulation of macromolecule metabolic process
	GO:0019222	Biological Process	Lower	regulation of metabolic process
	GO:0031323	Biological Process	Lower	regulation of cellular metabolic process
	GO:0032501	Biological Process	Lower	multicellular organismal process
	GO:0051171	Biological Process	Lower	regulation of nitrogen compound metabolic process
	GO:0043228	Cellular Component	Lower	non-membrane-bounded organelle
	GO:0043232	Cellular Component	Lower	intracellular non-membrane-bounded organelle
	GO:0016787	Molecular Function	Lower	hydrolase activity

Supplementary Table S3. Shared structural polymorphism gene enrichment. The concentration of GO terms within genes found in SSPs were tested against all gene GO terms. Significantly higher or lower GO terms are listed.

	E. melliodora	*E. melliodora*
**Genome-wide**	0.05016 ± 0.03074	0.04879 ± 0.03188
**Chromosome 1**	0.04996 ± 0.03134	0.04782 ± 0.03180
**Chromosome 2**	0.05012 ± 0.03069	0.04989 ± 0.03180
**Chromosome 3**	0.04871 ± 0.02954	0.04803 ± 0.03136
**Chromosome 4**	0.04978 ± 0.03159	0.04944 ± 0.03360
**Chromosome 5**	0.04919 ± 0.02971	0.04714 ± 0.03009
**Chromosome 6**	0.05196 ± 0.03126	0.04999 ± 0.03327
**Chromosome 7**	0.04946 ± 0.02985	0.04899 ± 0.03079
**Chromosome 8**	0.05002 ± 0.02996	0.04826 ± 0.03074
**Chromosome 9**	0.05012 ± 0.03186	0.04746 ± 0.03142
**Chromosome 10**	0.05197 ± 0.03194	0.05090 ± 0.03439
**Chromosome 11**	0.05167 ± 0.03208	0.04896 ± 0.03282
**Range of average**	0.04871 - 0.05167	0.04714 - 0.04896

Supplementary Table S4. Recombination rate estimates. Recombination rates were calculated in 1 Kbp windows and averaged across chromosomes. Rates are shown with standard deviation. Chromosomes coloured with darker green have higher average recombination rates.

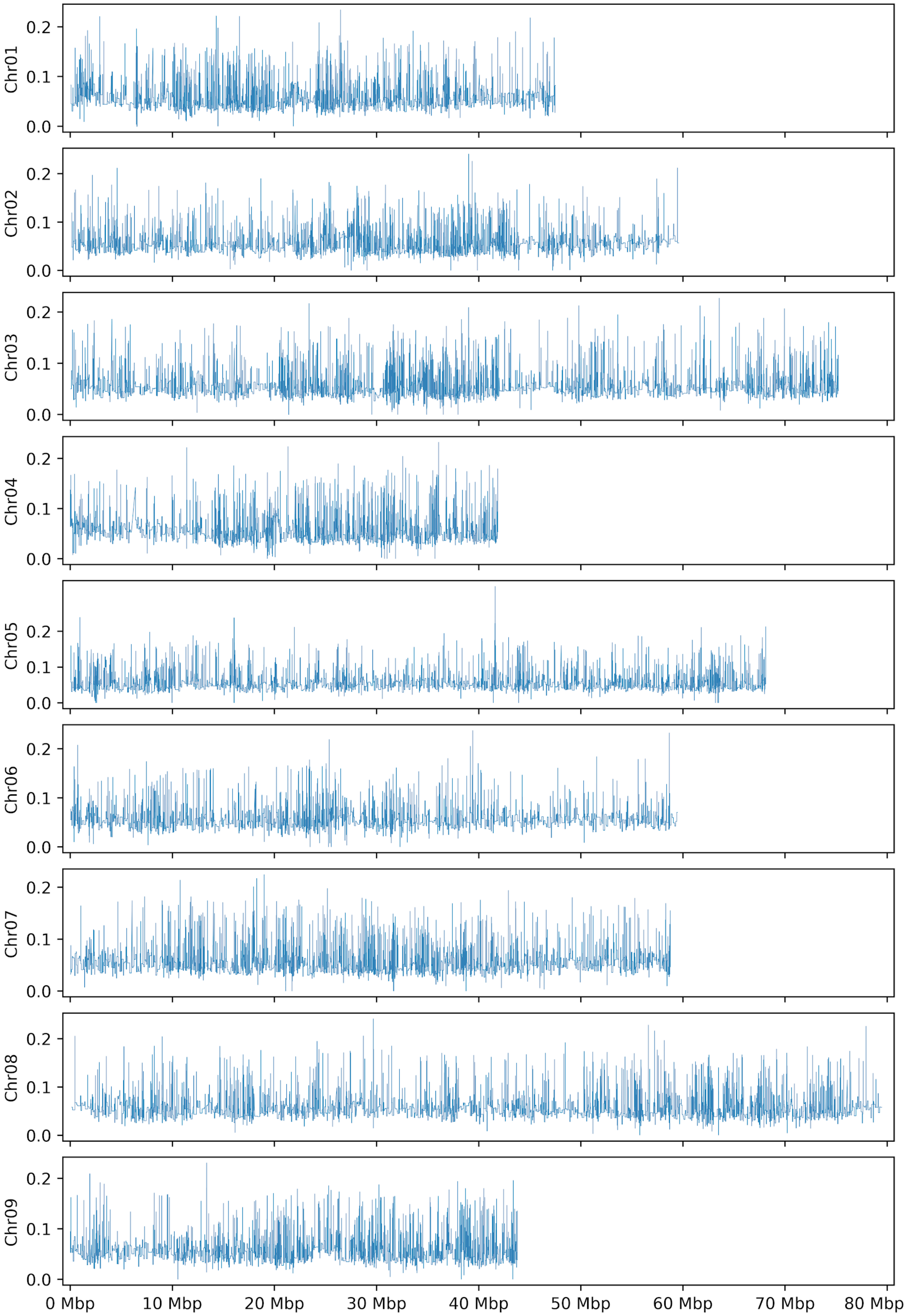
Chr01
Chr02
Chr03
Chr04
Chr05
Chr06
Chr07
Chr08
Chr09
0.0
0.1
0.2
0 Mbp
10 Mbp
20 Mbp
30 Mbp
40 Mbp
50 Mbp
60 Mbp
70 Mbp
80 Mbp

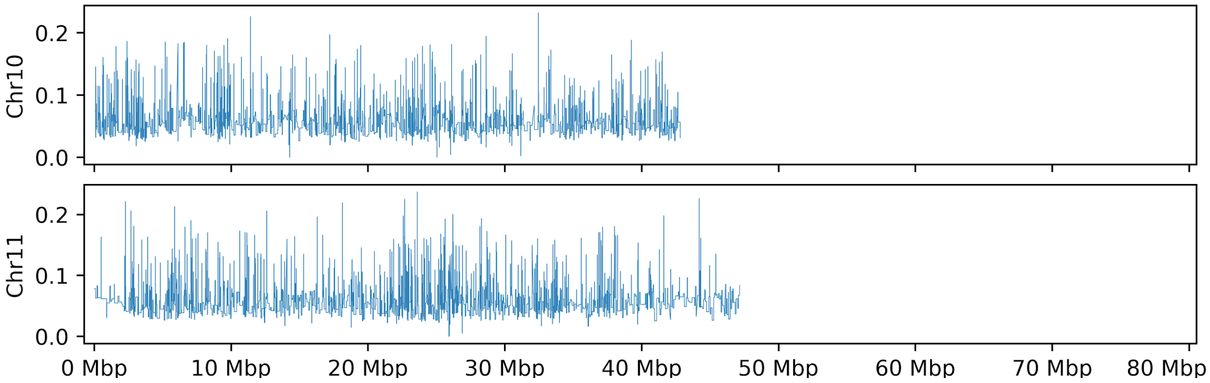

Supplementary Figure S7. *E. melliodora* recombination

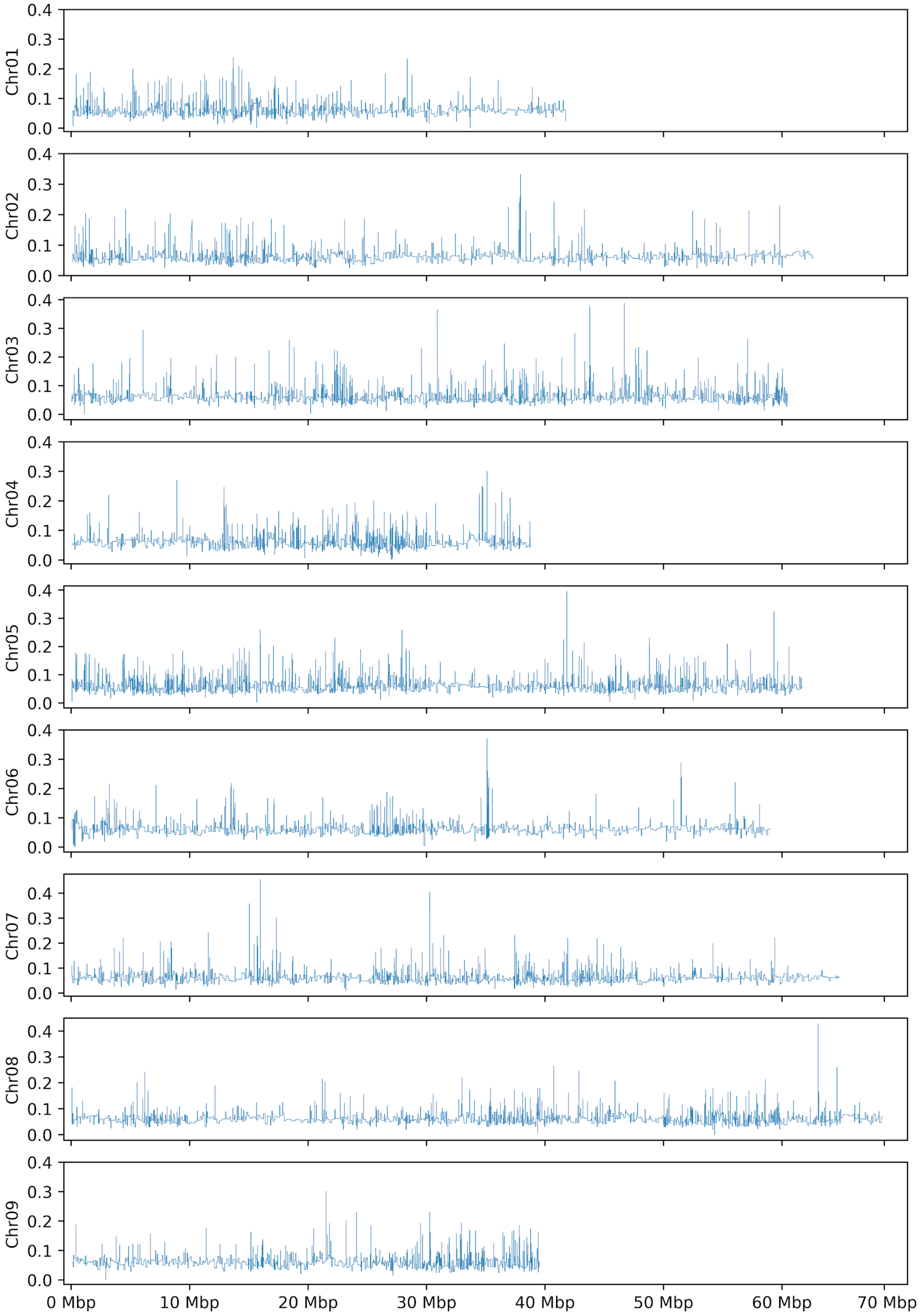
Chr01
Chr02
Chr03
Chr04
Chr05
Chr06
Chr07
Chr08
Chr09
0.0
0.1
0.2
0.3
0.4
0 Mbp
10 Mbp
20 Mbp
30 Mbp
40 Mbp
50 Mbp
60 Mbp
70 Mbp

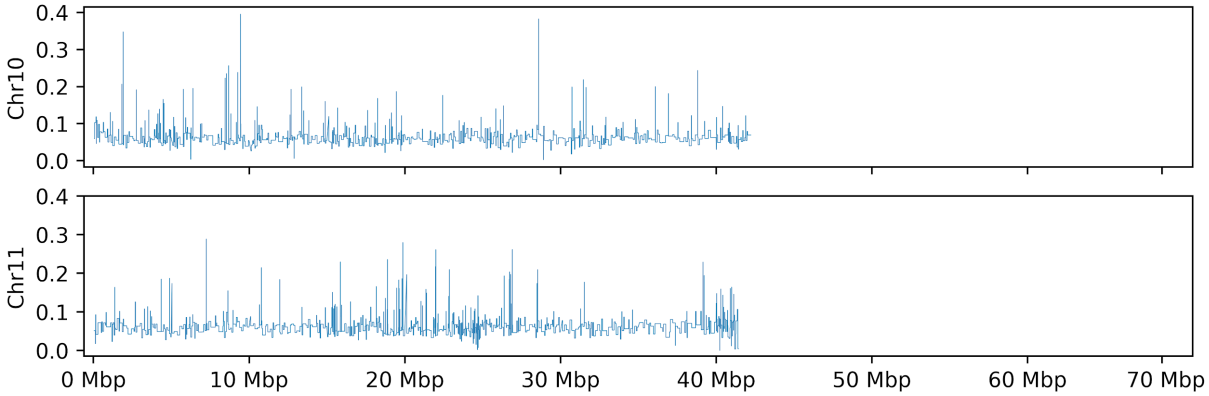

Supplementary Figure S8. *E. sideroxylon* recombination

		E. melliodora	*E. sideroxylon*
**Syntenic**	**Gene**	0	0
	**Transposon**	0	0
	**Inverted**	0.9982	0.9942
	**Translocated**	1	0.5106
	**Duplicated**	0.0023	0.0151
	**Unaligned**	1	0.9969
**Gene**	**Syntenic**	0	0
	**Transposon**	0	0
	**Inverted**	0.9504	0.5622
	**Translocated**	0	0
	**Duplicated**	0	0
	**Unaligned**	0	0
**Transposon**	**Syntenic**	0	0
	**Gene**	0	0
	**Inverted**	0.9997	0.9981
	**Translocated**	0	0.002
	**Duplicated**	0	0
	**Unaligned**	0	0
**Inverted**	**Syntenic**	0.9982	0.9942
	**Gene**	0.9504	0.5622
	**Transposon**	0.9997	0.9981
	**Translocated**	0.9983	1
	**Duplicated**	1	1
	**Unaligned**	0.9977	0.9969
**Translocated**	**Syntenic**	1	0.5106
	**Gene**	0	0
	**Transposon**	0	0.002
	**Inverted**	0.9983	1
	**Duplicated**	0.699	1
	**Unaligned**	1	0.801
**Duplicated**	**Syntenic**	0.0023	0.0151
	**Gene**	0	0
	**Transposon**	0	0
	**Inverted**	1	1
	**Translocated**	0.699	1
	**Unaligned**	0.0138	0.3032
**Unaligned**	**Syntenic**	1	0.9969
	**Gene**	0	0
	**Transposon**	0	0
	**Inverted**	0.9977	0.9969
	**Translocated**	1	0.801
	**Duplicated**	0.0138	0.3032

Supplementary Table S5. Pairwise rho Tukey's test p-values. Green indicates a significant difference (P<=0.05).

		E. melliodora	*E. sideroxylon*
**Syntenic**	**Gene**	0	0
	**Transposon**	0	0
	**Inverted**	0.9996	0.999
	**Translocated**	0	0
	**Duplicated**	0	0
	**Unaligned**	0	0
**Gene**	**Syntenic**	0	0
	**Transposon**	0	0
	**Inverted**	1	0.9605
	**Translocated**	0	0
	**Duplicated**	0	0
	**Unaligned**	0	0
**Transposon**	**Syntenic**	0	0
	**Gene**	0	0
	**Inverted**	0.8074	0.8693
	**Translocated**	0.1234	0.9997
	**Duplicated**	0.9983	0
	**Unaligned**	1	0
**Inverted**	**Syntenic**	0.9996	0.999
	**Gene**	1	0.9605
	**Transposon**	0.8074	0.8693
	**Translocated**	0.5745	0.8445
	**Duplicated**	0.7877	0.9975
	**Unaligned**	0.8103	1
**Translocated**	**Syntenic**	0	0
	**Gene**	0	0
	**Transposon**	0.1234	0.9997
	**Inverted**	0.5745	0.8445
	**Duplicated**	0.2856	0.004
	**Unaligned**	0.1464	0
**Duplicated**	**Syntenic**	0	0
	**Gene**	0	0
	**Transposon**	0.9983	0
	**Inverted**	0.7877	0.9975
	**Translocated**	0.2856	0.004
	**Unaligned**	0.9987	0.2449
**Unaligned**	**Syntenic**	0	0
	**Gene**	0	0
	**Transposon**	1	0
	**Inverted**	0.8103	1
	**Translocated**	0.1464	0
	**Duplicated**	0.9987	0.2449

Supplementary Table S6. Pairwise Fst Tukey's test p-values. Green indicates a significant difference (P<=0.05).

		E. melliodora	*E. sideroxylon*
**Syntenic**	**Gene**	0	0
	**Transposon**	0	0
	**Inverted**	0.9349	0.4655
	**Translocated**	0	0.0029
	**Duplicated**	0	0
	**Unaligned**	0	0
**Gene**	**Syntenic**	0	0
	**Transposon**	0	0
	**Inverted**	0.4392	0.3614
	**Translocated**	0	0
	**Duplicated**	0.9991	0
	**Unaligned**	0	0
**Transposon**	**Syntenic**	0	0
	**Gene**	0	0
	**Inverted**	1	0.9755
	**Translocated**	0.0107	0.493
	**Duplicated**	0	0
	**Unaligned**	0	0
**Inverted**	**Syntenic**	0.9349	0.4655
	**Gene**	0.4392	0.3614
	**Transposon**	1	0.9755
	**Translocated**	0.9895	0.8918
	**Duplicated**	0.4565	1
	**Unaligned**	0.4602	0.0158
**Translocated**	**Syntenic**	0	0.0029
	**Gene**	0	0
	**Transposon**	0.0107	0.493
	**Inverted**	0.9895	0.8918
	**Duplicated**	0	0
	**Unaligned**	0	0
**Duplicated**	**Syntenic**	0	0
	**Gene**	0.9991	0
	**Transposon**	0	0
	**Inverted**	0.4565	1
	**Translocated**	0	0
	**Unaligned**	0	0
**Unaligned**	**Syntenic**	0	0
	**Gene**	0	0
	**Transposon**	0	0
	**Inverted**	0.4602	0.0158
	**Translocated**	0	0
	**Duplicated**	0	0

Supplementary Table S7. Pairwise SNP per Kilobase Tukey's test p-values. Green indicates a significant difference (P<=0.05).
