# Supplementary material for: Exploring the role of polymorphic interspecies structural variants in reproductive isolation and adaptive divergence in Eucalyptus
Source: Gigascience. 2024 Jun 13;13:giae029. doi: 10.1093/gigascience/giae029 (PMC11170218; doi:10.1093/gigascience/giae029)
Supplement: giae029_GIGA-D-23-00337_Original_Submission [file giae029_giga-d-23-00337_original_submission.pdf]

GigaScience

Exploring polymorphic interspecies structural variants in Eucalyptus: Unravelling Their Role in Reproductive Isolation and Adaptive Divergence.

--Manuscript Draft--

Manuscript Number:	GIGA-D-23-00337
Full Title:	Exploring polymorphic interspecies structural variants in Eucalyptus: Unravelling Their Role in Reproductive Isolation and Adaptive Divergence.
Article Type:	Research
Funding Information:	Australian Research Council (CE140100008) — Dr Justin Borevitz<br>Australian Research Council (DP150103591) — Dr Rose L Andrew<br>Australian Research Council (DE190100326) — Dr Helen Bothwell
Abstract:	Structural variants (SVs) play a significant role in speciation and adaptation in many species, yet few studies have explored the prevalence and impact of different categories of SVs. We conducted a comparative analysis of long-read assembled reference genomes of closely related Eucalyptus species to identify candidate SVs potentially influencing speciation and adaptation. Interspecies SVs can be either fixed differences, or polymorphic in one or both species. To describe SV patterns, we employed short-read whole-genome sequencing on over 600 individuals of E. melliodora and E. sideroxylon, along with recent high quality genome assemblies. We aligned reads and genotyped interspecies SVs predicted between species reference genomes. Our results revealed that 49,756 of 58,025 and 39,536 of 47,064 interspecies SVs could be typed with short reads, in E. melliodora and E. sideroxylon respectively. Focusing on inversions and translocations, symmetric SVs which are readily genotyped within both populations, 24 were found to be structural divergences, 2,623 structural polymorphisms, and 928 shared structural polymorphisms. We assessed the functional significance of fixed interspecies SVs by examining differences in estimated recombination rates and genetic differentiation between species, revealing a complex history of natural selection. Shared structural polymorphisms displayed enrichment of potentially adaptive genes. Understanding how different classes of genetic mutations contribute to genetic diversity and reproductive barriers is essential for understanding how organisms enhance fitness, adapt to changing environments, and diversify. Our findings reveal the prevalence of interspecies SVs and elucidate their role in genetic differentiation, adaptive evolution, and species divergence within and between populations.
Corresponding Author:	Scott Ferguson<br>Australian National University<br>Acton, AUSTRALIA
Corresponding Author Secondary Information:	
Corresponding Author's Institution:	Australian National University
Corresponding Author's Secondary Institution:	
First Author:	Scott Ferguson
First Author Secondary Information:	
Order of Authors:	Scott Ferguson<br>Ashley Jones<br>Kevin Murray<br>Rose L Andrew<br>Helen Bothwell

Powered by Editorial Manager® and ProduXion Manager® from Aries Systems Corporation

	Benjamin Schwessinger
	Justin Borevitz
**Order of Authors Secondary Information:**	
**Additional Information:**	
**Question**	**Response**
Are you submitting this manuscript to a special series or article collection?	No
**Experimental design and statistics**<br><br>Full details of the experimental design and statistical methods used should be given in the Methods section, as detailed in our Minimum Standards Reporting Checklist. Information essential to interpreting the data presented should be made available in the figure legends.<br><br>Have you included all the information requested in your manuscript?	Yes
**Resources**<br><br>A description of all resources used, including antibodies, cell lines, animals and software tools, with enough information to allow them to be uniquely identified, should be included in the Methods section. Authors are strongly encouraged to cite Research Resource Identifiers (RRIDs) for antibodies, model organisms and tools, where possible.<br><br>Have you included the information requested as detailed in our Minimum Standards Reporting Checklist?	Yes
**Availability of data and materials**<br><br>All datasets and code on which the conclusions of the paper rely must be either included in your submission or deposited in publicly available repositories (where available and ethically appropriate), referencing such data using	Yes

a unique identifier in the references and in the “Availability of Data and Materials” section of your manuscript.<br><br>Have you have met the above requirement as detailed in our Minimum Standards Reporting Checklist?	

Powered by Editorial Manager® and ProduXion Manager® from Aries Systems Corporation

Manuscript Click here to access/download;Manuscript;version 5.Gigascience.unlink.pdf

Title: Exploring polymorphic interspecies structural variants in Eucalyptus: Unravelling Their Role in Reproductive Isolation and Adaptive Divergence.

Running title: Interspecies polymorphic and fixed structural variations

Scott Ferguson[1*], Ashley Jones[1], Kevin Murray[1,2], Rose L. Andrew[3], Benjamin Schwessinger[1], Helen Bothwell[1,4] , and Justin Borevitz[1]

1. Research School of Biology, Australian National University, Canberra, Australian Capital Territory, Australia
2. Department of Molecular Biology, Max Planck Institute for Biology Tübingen, Tübingen,Germany
3. Botany & N.C.W. Beadle Herbarium, School of Environmental and Rural Science, University of New England, Armidale, NSW 2351, Australia.
4. Warnell School of Forestry & Natural Resources, University of Georgia, 180 E Green St, Athens, 30602, GA, United States

*. First author

Corresponding author
Scott Ferguson
scott.ferguson.papers@gmail.com

Abstract

Structural variants (SVs) play a significant role in speciation and adaptation in many species, yet few studies have explored the prevalence and impact of different categories of SVs. We conducted a comparative analysis of long-read assembled reference genomes of closely related *Eucalyptus* species to identify candidate SVs potentially influencing speciation and adaptation. Interspecies SVs can be either fixed differences, or polymorphic in one or both species. To describe SV patterns, we employed short-read whole-genome sequencing on over 600 individuals of *E. melliodora* and *E. sideroxylon*, along with recent high quality genome assemblies. We aligned reads and genotyped interspecies SVs predicted between species reference genomes. Our results revealed that 49,756 of 58,025 and 39,536 of 47,064 interspecies SVs could be typed with short reads, in *E. melliodora* and *E. sideroxylon* respectively. Focusing on inversions and translocations, symmetric SVs which are readily genotyped within both populations, 24 were found to be structural divergences, 2,623 structural polymorphisms, and 928 shared structural polymorphisms. We assessed the functional significance of fixed interspecies SVs by examining differences in estimated recombination rates and genetic differentiation between species, revealing a complex history of natural selection. Shared structural polymorphisms displayed enrichment of potentially adaptive genes. Understanding how different classes of genetic mutations contribute to genetic diversity and reproductive barriers is essential for understanding how organisms enhance fitness, adapt to changing environments, and diversify. Our findings reveal the prevalence of interspecies SVs and elucidate their role in genetic differentiation, adaptive evolution, and species divergence within and between populations.

Keywords

1. Eucalyptus
2. Structural variations

3. Adaptive evolution
4. Genome divergence
5. Comparative genomics

Introduction

Structural mutations that alter stretches of DNA greater than 50 bp in length have the potential to drastically change phenotypes [1–3] and contribute to population divergence and speciation [4,5]. Typically termed chromosomal rearrangements or structural variations (SV), these large mutations include inversions, translocations, duplications, insertions, and deletions [6]. Until recently however, technological constraints, namely sequencing read lengths, have inhibited their discovery [7], and their role in population evolutionary processes remains poorly understood [8]. Using third-generation long-read sequencing, such as those offered by Oxford Nanopore Technologies and PacBio, evolutionary genomic studies can now affordably assemble highly contiguous genomes of several individuals across related species. The next challenge is to perform population scale SV discovery and examine the role of SVs in population divergence and speciation.

Structural variation can occur in all parts of the genome: coding, noncoding, and repetitive regions such as transposons, telomeres and centromeres. When they occur within coding regions, they may alter regulatory elements, introns, exons, whole genes, or multiple genes [9,10]. Even when they do not occur within coding regions they can change the chromatin structure and impact gene expression [11,12]. Different SV types are known or predicted to have different genomic effects. Inversions can inhibit recombination between different arrangements, reducing the overall recombination rates between homologous chromosome pairs, and fixing the alleles captured within their bounds [13]. Inversion-linked, cosegregating alleles can become reproductively isolated and purged through underdominant selection, due to increased sterility of heterozygous individuals [14–16]. However, a novel inversion, if

adaptive, may provide enough selective advantages to outweigh its disadvantages, be selected for, and rise to high frequency within populations [17,18]. Translocations, while less studied than other rearrangements [19], may have similar genomic effects as inversions [20]. Duplications, highly common and also likely to be selected against [21,22], could be preserved due to their ability to acquire new function (neofunctionalisation) or by retaining a subset of original function (subfunctionalisation) [22–25]. Large (> 50 bp) insertions and deletions, which are often genotyped as presence/absence variants (PAVs), copy number variations (CNV), or gene duplications, are also very common within genomes [8,26]. These SVs are known to impact genes and gene structure, and to affect phenotypes [27,28], although many can also be neutral.

An ancestral population, once highly syntenic, undergoes division into two non-interbreeding groups, with structural variations (SVs) emerging between them, Figure 1. These interspecies SVs can be genotyped as fixed within one species, leading to structural divergence (SD) or polymorphic within one species, termed structural polymorphisms (SP) [29]. Adding complexity, SVs can also be genotyped as polymorphic in both populations, referred to as shared structural polymorphisms (SSP). To classify interspecies SVs, genotyping within both species is essential, enabling us to categorise them based on their presence/absence in population 1 and population 2 as fixed/absent (SD), fixed/polymorphic (SP), absent/polymorphic (SP), or polymorphic/polymorphic (SSP). The rate at which SVs are SD, SP, or SSP is unknown; however, rates will depend on the evolutionary distance between populations or species, effective population size, and mutation rate, among other factors. If the status of an SV remains uncertain, inferences of its impact on divergence and adaptation are difficult.

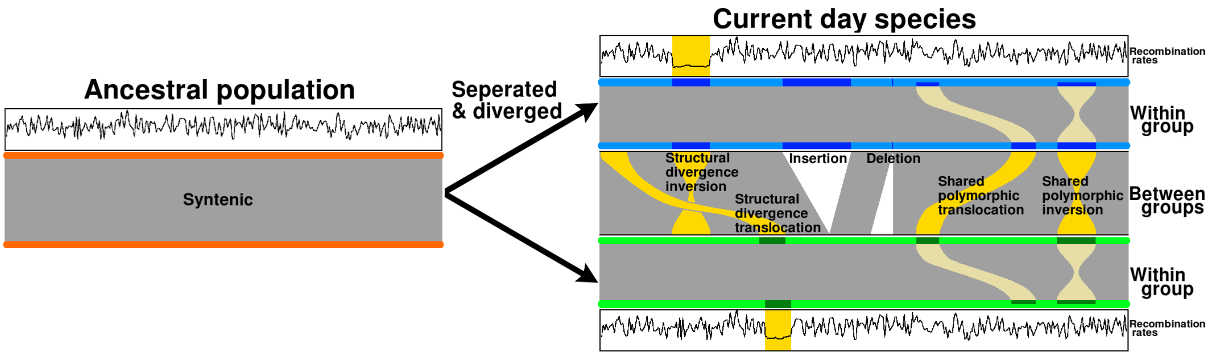

Figure 1. Structural variations within sister species. The once highly syntenic ancestral population separates and divides into two non-interbreeding groups. Structural variations, which reduce genome-wide synteny, discovered between the two groups may be genotyped within populations as fixed or polymorphic. When fixed in a single population, SVs become a structural divergence (SD). If polymorphic within one population, SVs become structural polymorphisms (SP), or if polymorphic in both populations a shared structural polymorphisms (SSP). The different classes of population genotyped SVs may have different impacts on recombination rates, divergence, and adaptation.

Analysing the genomic differences between recently diverged species has revealed genome regions involved in reproductive isolation [30], adaptive genes [31], and the genome-wide landscape of diversification between and within chromosomes [32–34]. Here using two closely related *Eucalyptus* species, *E. melliodora* and *E. sideroxylon* [35,36], we genotype SVs within their respective populations and calculate their rates of population variability. Structural variation rates are compared to find evidence of SD, SP, and SSP. Additionally, we examine recombination rates and Fixation Index (F_{ST}) within population fixed SVs to assess allele fixation and accelerated evolution between populations.

Results

Genome scaffolding

We generated Hi-C data and performed Hi-C scaffolding to order, orient, and combine contigs into pseudo-chromosomes for *E. melliodora*. Hi-C sequencing generated 45.48 Gbp in 151,590,503 paired reads, giving an estimated genome coverage of 71.14x. After aligning Hi-C reads to *E. melliodora's* contigs and identifying PCR duplicates, 18,507,548 (12.21%) read pairs were found to contain linkage information. Further examination showed that 9,612,532 (6.34%) read pairs spanned contigs, and 8,895,016 (5.87%) read pairs were

contained within a single contig. Non-informative reads were either chimeric, unmapped, PCR duplicates, or had low mapping quality (MAPQ < 30, mostly due to multi-mapping of short reads to repeat regions). For all Hi-C statistics see Supplementary Table S1. Using 3D-DNA *E. melliodora's* contigs were scaffolded (Supplementary Figures S1 and S2). Contigs for *E. sideroxylon* were syntenically scaffolded against *E. melliodora's* Hi-C scaffolded genome. Both BUSCO and LAI scores indicate that both genomes are highly complete (Table 1).

Table 1. Genome assembly statistics for *E. melliodora* and *E. sideroxylon*.

	E. melliodora	***E. sideroxylon***
**Scaffolded genome Size (bp)**	639,266,298	592,154,182
**% of genome in scaffolds**	97.60%	98.15%
**Scaffold N50 (Mbp)**	59.47	60.48
**Contig N50 (Mbp)**	1.87	5.22
**Contig count**	564	297
**BUSCO complete**	98.54%	96.47%
**LAI**	18.31	18.70
**Repetitive % (TE %)**	48.50% (47.13%)	47.83% (46.58%)
**Gene candidates**	58,902	57,299
**Proportion of genome in gene candidates**	21.85%	21.04%

Annotation (repeats & genes)

Both genomes were annotated for transposable elements (TE), simple repeats, and genes (Table 1). Transposable elements and simple repeats were annotated with genome-specific *de novo* repeat libraries. Soft repeat masked genomes were next annotated for genes.

Synteny and structural variation annotation

Shared sequences between *E. melliodora* and *E. sideroxylon* were identified using nucmer from the MUMmer toolset. Subsequently, using SyRI, shared sequences were classified as syntenic, inverted, translocated, or duplicated, and both genomes accordingly annotated for these regions. Additionally, both genomes were annotated for unaligned regions, which are unique to each genome, resulting from insertions, deletions, or divergence beyond recognition. 85.94% of *E. melliodora's* genome was found to be shared with *E. sideroxylon's*

genome; conversely, 87.70% of *E. sideroxylon's* genome was found to be shared with *E. melliodora's* genome. The majority of shared sequences were syntenic. A more detailed analysis of alignment types showed that syntenic regions are, on average, frequent and large, inversions are rare and typically very large, translocations are moderately sized and frequent, duplications are very frequent and small, and unaligned regions are very frequent and small (Table 2, Figure 2).

Table 2. Proportion, number of regions, and total amount the genome that was found to be syntenic, rearranged, and unaligned within *E. melliodora* and *E. sideroxylon* when their genomes were aligned.

Genome	Statistic	Syntenic	Inversion	Translocation	Duplication	Unaligned
E. melliodora	**Count**	19,137	232	10,645	26,762	20,386
	**Average size (Kbp)**	16.18 ± 20.93	202.96 ± 1097.87	11.41 ± 69.65	5.25 ± 32.57	4.30 ± 7.13
	**Total (Mbp)**	309.60	47.09	121.49	140.63	87.74
	**Proportion**	49.62%	7.55%	19.47%	22.54%	14.06%
E. sideroxylon	**Count**	19,137	232	10,645	20,102	18,777
	**Average size (Kbp)**	16.14 ± 20.87	177.67 ± 851.99	11.29 ± 65.34	4.30 ± 33.33	3.81 ± 6.67
	**Total (Mbp)**	308.78	41.22	120.22	86.44	71.51
	**Proportion**	53.13%	7.09%	20.69%	14.87%	12.30%

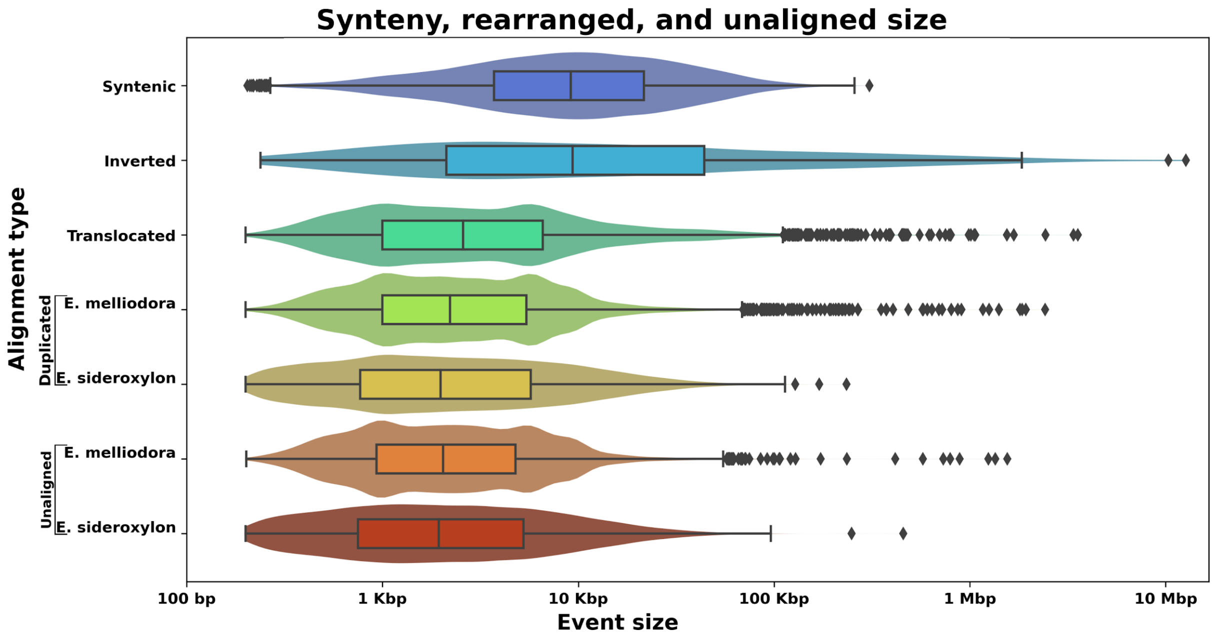

Figure 2. Synteny, rearranged, and unaligned event sizes. As syntenic, inverted, and translocated regions are approximately the same size with each genome (differing only by small indels) these alignment types are only shown for *E. melliodora*. Duplications and

unaligned regions are unique to each genome and as such are shown for both *E. melliodora* and *E. sideroxylon*. See Supplementary Figure S3 for all event sizes for both genomes.

Variant calling and PCA

After calculating the total number of sequenced bases per sample and removing samples which had low coverage (< 10x), *E. melliodora's* samples yielded on average 9.49 Gbp (range: 6.27 Gbp - 27.22 Gbp). Similarly, *E. sideroxylon's* samples yielded on average 9.10 Gbp (range: 5.82 Gbp - 28.87 Gbp). Examined across both populations and both reference genomes, coverage averaged 15.40x (range: 10.00x - 48.7x). After aligning both populations sequences to both reference genomes, and filtering out samples with low alignment (< 75%), an average of 96.55% (range: 77.91% - 98.80%) of reads aligned to both genomes. Variants were called for the remaining samples, resulting in four dataset; (reference genome - population species) *E. melliodora - E. melliodora*, *E. melliodora - E. sideroxylon*, *E. sideroxylon - E. melliodora*, and *E. sideroxylon - E. sideroxylon* (Table 3, Figure 3).

Principal component analysis identified 15 samples that were mislabelled, hybrid, and outlier samples, which were removed (supplementary Figures S4). After removal of these samples the PCA showed two distinct species groups, Figure 4. Within the combined *E. melliodora* dataset, 32.45 million sites, or 5.20% of the genome, were found to be variable. 49.61% of these SNPs were found segregating within both species, 21.76% were private to *E. melliodora,* and as expected a larger proportion, 28.63%, were private to the non-reference species *E. sideroxylon*. Within the combined *E .sideroxylon* SNP dataset we observed the same pattern; 31.28 million SNPs (5.38% of the genome) were found, of which 49.68% segregated within both species, and 20.24% were private to *E. sideroxylon*, while a larger proportion, 30.08%, was found within the non-reference species (Table 3).

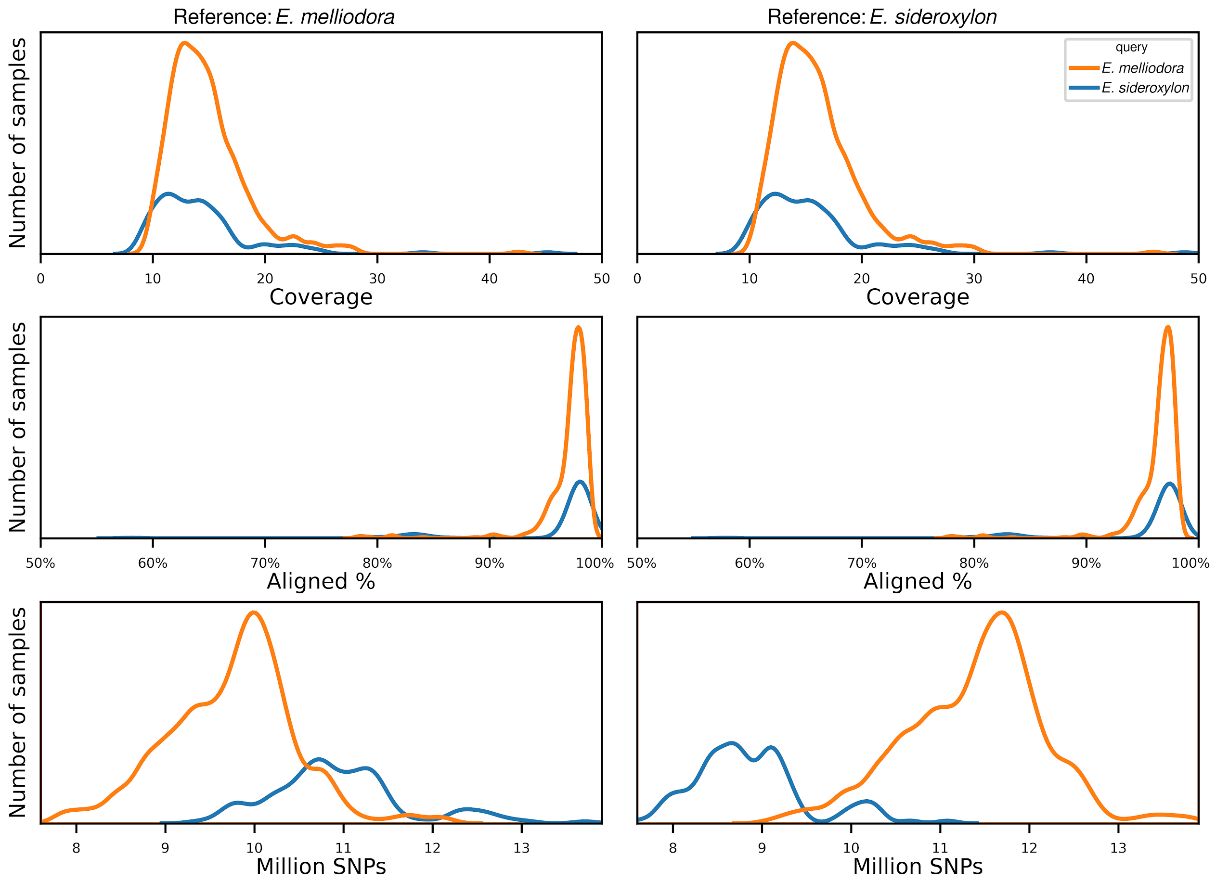

Figure 3. Sample coverage, alignment, and SNP distributions. Left figures use *E. melliodora* as the reference, showing the per sample histogram of sample coverage, percent of reads successfully aligned to reference, and the number of SNPs detected. Right figures are identical, except use *E. sideroxylon* as the reference.

Table 3. Short-read sequencing, alignment, SNP, and recombination rate estimate statistics.

	**Reference species**	***E. melliodora***		***E. sideroxylon***	
	**Population species**	***E. melliodora***	***E. sideroxylon***	***E. melliodora***	***E. sideroxylon***
	All samples	459	154	459	154
	Filtered samples	425	138	425	138
**Estimated read coverage**	Average	14.90	16.03	14.82	15.52
	Range	10.00 - 42.58	10.59 - 45.97	10.11 - 45.16	10.06 - 48.76
**Read alignment**	Average	97.06%	96.43%	96.32%	95.81%
	Range	78.40% - 98.80%	78.70% - 98.56%	77.91% - 98.10%	78.38% - 98.02%
**SNPs (million)**	Average	9.74	10.93	11.36	8.88
	Range	6.77 - 13.50	8.30 - 13.80	7.07 - 14.80	7.61 - 12.05
	Total	23.16	25.39	24.96	21.87
	Grand total	32.46		31.28	
**Recombination rate estimates**	Genome-wide	0.050 ± 0.031	-	0.049 ± 0.033	-
	Chromosome average range	0.049 - 0.052	-	0.047 - 0.049	-

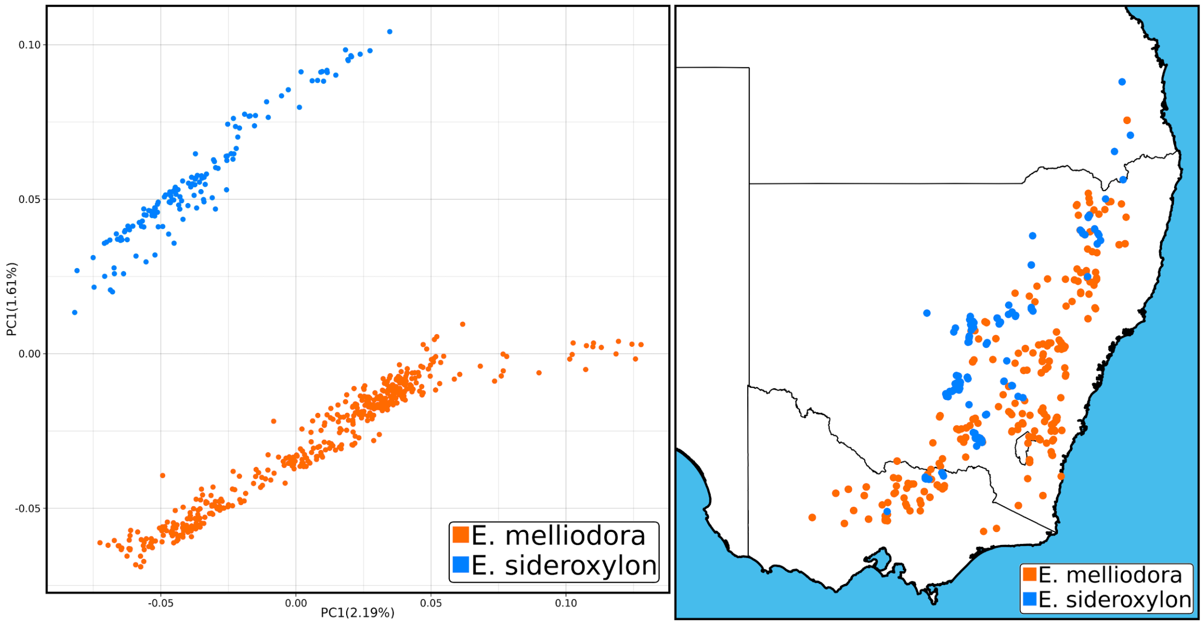

Figure 4. Principal component analysis and sample distribution. Left PCA plot uses *E. melliodora* as the reference genome following the removal of mislabelled, hybrid, and outlier samples. Right map shows the spatial distribution of samples across south eastern Australia. For PCA using *E. sideroxylon* as the reference see Supplementary Figure S5.

Structural variation genotyping

Interspecies SVs identified between *E. melliodora* and *E. sideroxylon* may be categorised as SD, SP, or SSP. Structural divergences are any event fixed within one species and absent from the other. Structural polymorphisms are any event fixed or absent in one species and polymorphic in the other. Shared structural polymorphisms are SVs that are polymorphic in both populations, Figure 1. Genotyping an SV as SD, SP, or SSP requires examination within both species. While symmetric rearrangements, such as inversions and translocations, can be directly genotyped in both populations, duplications pose challenges due to their asymmetry. Although converting duplications into insertions for short-read genotyping is possible, accurately placing them within the opposite genome is difficult and may result in false negative genotypes. Additionally, genotyping unaligned regions as insertions or deletions introduces uncertainties, especially as they may represent insertions, deletions, or divergent sequences. Short-read alignments with low mapping scores may confound genotyping of unaligned regions [66,67]. Hence, we approach unaligned regions with caution, refrain from categorising duplications as SD, SP, or SSP, and focus our

analysis on inversions and translocations for more reliable results. All analyses are performed per allele (2 x population size), not per sample.

Genotyping SVs with short-read alignments resulted in the successful genotyping of 81.11% and 79.46% of SVs in *E. melliodora* and *E. sideroxylon*, respectively (Figure 5). The majority of SVs were found to be fixed (60.65% - 85.10%) or polymorphic (14.84% - 38.57%), with the remaining small proportion (0% - 1.45%) being private to the reference or assembly/scaffolding artefacts. To categorise symmetric interspecies SVs as SD, SP, or SSP we combined the status of fixed inversions (*E. melliodora*: 130; *E. sideroxylon*: 174), polymorphic inversions (*E. melliodora*: 66; *E. sideroxylon*: 37), fixed translocations (*E. melliodora*: 5,652; *E. sideroxylon*: 6,634), polymorphic translocations (*E. melliodora*: 3,288; *E. sideroxylon*: 2,117) across both species, Figure 6. The analysis revealed that the majority of inversions and translocations were either fixed in both species or not successfully genotyped in both species, representing SVs private to the reference genome or assembly/scaffolding artefacts. The remaining proportion consisted of SPs (inversions: 25.98%, translocations: 24.80%) or SSPs (inversions: 7.79%, translocations: 8.81%).

Examination of polymorphic SVs revealed a bimodal distribution of alleles containing the SV, Figure 5. Polymorphic SVs were either very frequently (> 90%) genotyped or very infrequently (< 10%) genotyped within the two species. However, while bimodally distributed, the very frequent SV peak was found to be much higher than the very infrequent.

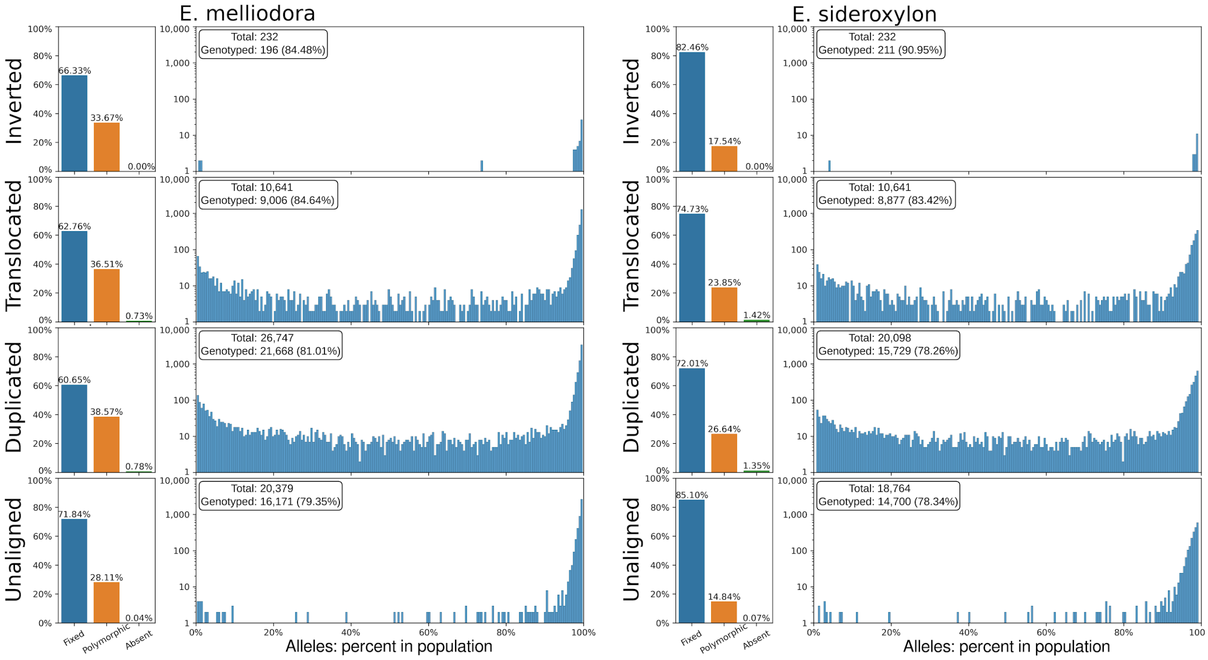

Figure 5. Interspecies SVs and unaligned region frequencies within *E. melliodora* and *E. sideroxylon*.

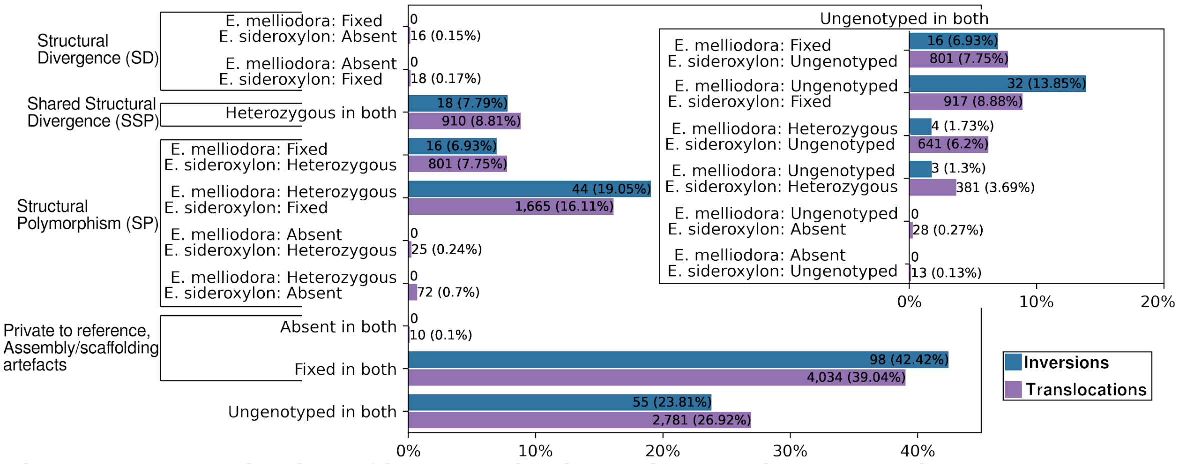

Figure 6. Categorisation of interspecies inversions and translocations as SD, SP, and SSP.

Structural variation linkage

Linked variations are those that co-occur more often than would be expected by random chance. Structural variations may be linked by physical proximity, drift or evolution. Evolutionarily linked SVs are likely to contribute to an individual's survivability and be required for gamete viability and/or the offspring's adaptive potential. To find evidence of SV linkage we measured correlations among all inversions and translocations for all individuals within both species. For efficient analysis, inversions and duplications were grouped by type (SD, SP, and SSP). Visual inspection of the resulting correlation heatmaps shows several

SVs are linked across all categories, Figure 7. To examine the potential role of physical proximity on SV linkage, we examined the distance between correlated SV pairs. 86.64% of SV pairs were found on different chromosomes. When on the same chromosome, SVs were at least 51 Kbp separated.

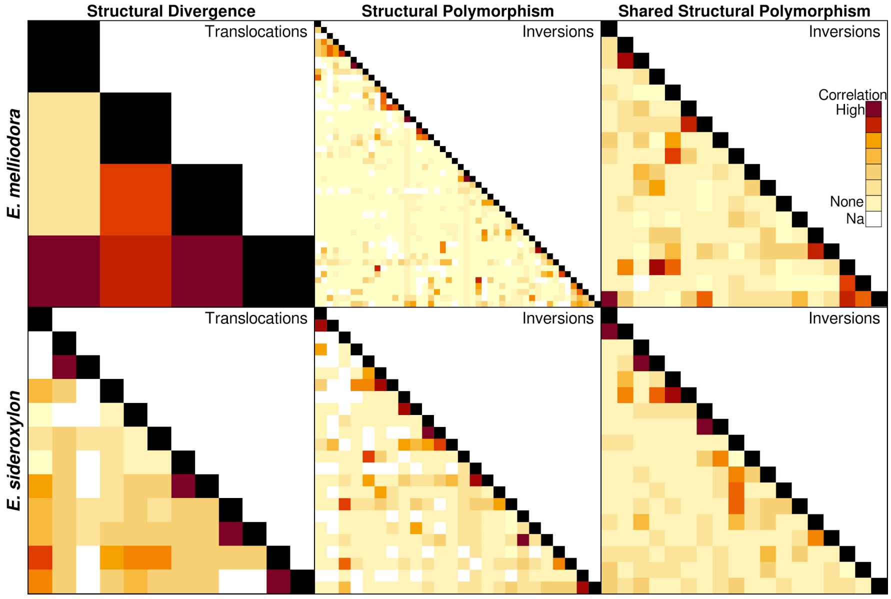

Figure 7. Correlation of SVs between samples. A positive correlation between SV implies that SVs exhibit a non-random association and suggests that these variants tend to co-occur within the population. Categories of SV not present were either empty, as in the case of inversion SD, or contained too many SV to visualise clearly, as in the case of translocation SP and translocation SSP. Undefined correlations, resulting from the failure of short-read to resolve presence/absence of SVs, were removed.

Shared Structural Polymorphisms COG terms

As SSPs are likely ancestral SVs that have survived drift, underdominant selection, and lineage divergence, they may contain genes of adaptive or other evolutionarily significant value. Examination of SSPs identified 281 gene candidates (*E. melliodora*: 145 and *E. sideroxylon*: 136), of which 247 (87%; *E. melliodora*: 125 and *E. sideroxylon*: 122) were functionally annotated into eggNOG orthogroups and grouped into COG (Clusters of Orthologous Groups) [68] categories, Figure 8. Similarly, all genes were functionally annotated and placed within COG categories. Comparing all genes to SSP genes indicates that SSP genes have an increased association with DNA replication, DNA recombination, DNA repair, post-translational modification, protein turnover, chaperones, signal

transduction, intercellular communication, and unexplored aspects of biology. Similarly, genes within SSPs have a decreased association with categories for fundamental cellular functions, such as protein synthesis, defence against pathogens, maintaining cellular integrity, providing structural support, and regulating crucial molecular processes involving amino acids, nucleotides, and coenzymes.

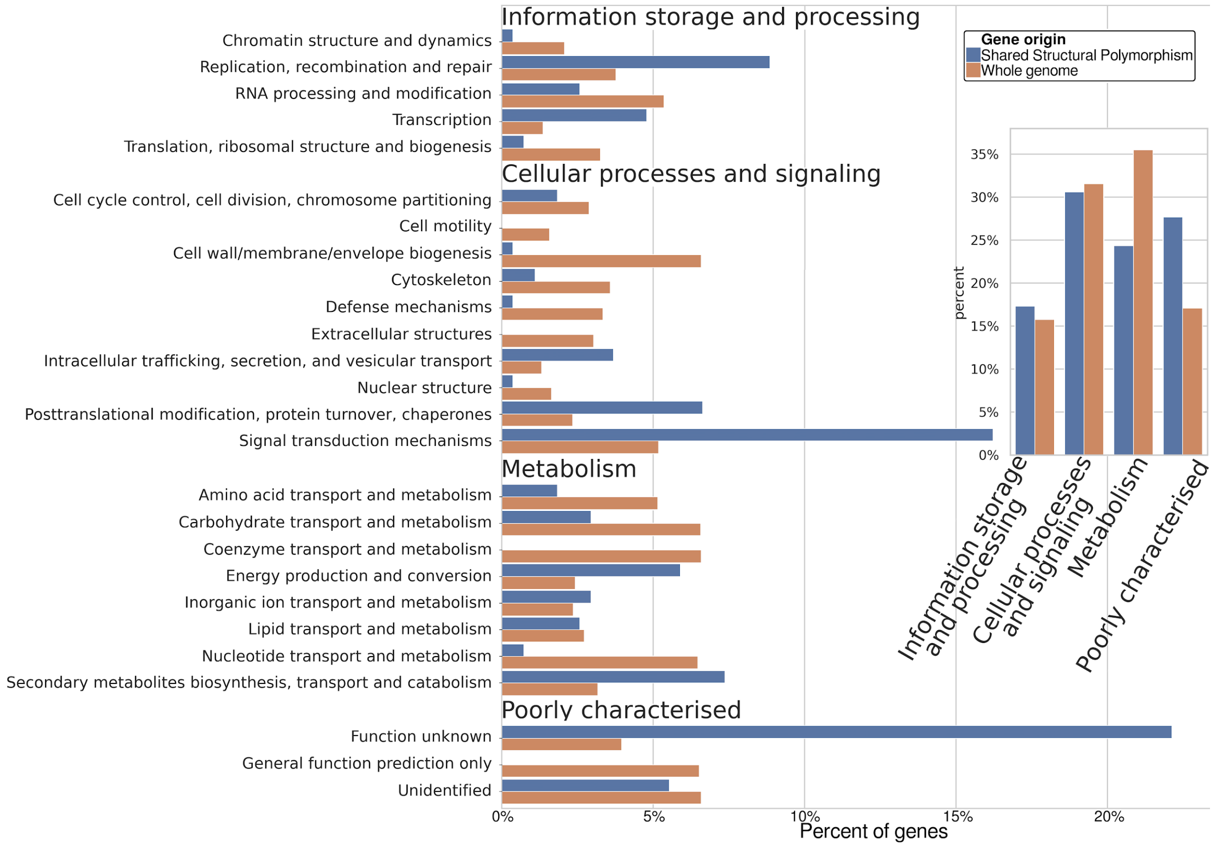

Figure 8. Clusters of Orthologous Groups (COG) terms for all genes and genes found within SSPs.

Effect of Synteny, rearranged, unaligned, and genes on recombination rates

After annotating SVs in both species and genotyping their frequencies, we calculated ρ across the reference genomes. As low-frequency SVs are unlikely to have a detectable effect on ρ, we considered only fixed SVs and excluded events shorter than 2 Kbp, as ρ was calculated within 1 Kbp windows. We also assessed the impact of genes and transposons larger than 2 Kbp on ρ. Prior to ρ calculations, we phased SNPs, initially achieving 20.56% linkage within haplotype blocks using read alignments, and subsequently completing

phasing with a HMM-based approach. After separation of SNPs into parental haplotypes, we found that *E. sideroxylon* consistently exhibited higher and more variable ρ compared to *E. melliodora*. Chromosome-specific recombination rates displayed notable variability without discernible patterns, Table 3, Supplementary Table S2, and Supplementary Figures S6 and S7.

An initial ANOVA assessment indicated differences in ρ for our different categories of genome regions, for both species (p-value; *E. melliodora*: 8.35×10^{-276} and *E. sideroxylon*: 1.85×10^{-272}). To determine if any region type/s were contributing to differences in ρ, we performed Tukey’s test, adjusting p-values to account for the total species error rate. Tukey's test for *E. melliodora* revealed that, in comparison to syntenic regions, average ρ was higher for genes, transposons, inversions, and duplications, Figure 9A. However, statistically significant differences were observed only for genes, transposons, and duplications. Notably, genes followed by transposons exhibited significantly higher ρ than all other types of regions, while duplications showed higher values than translocations and unaligned regions. Inversions exhibited a wider confidence interval (CI) due to their lower number of events. A similar pattern was observed by Tukey's test for *E. sideroxylon*. While genome-wide statistical observations of ρ were unrevealing, many SVs were observed having ρ less than the mean syntenic, Figure 9C and 9D. For detailed significance testing results refer to Supplementary Table S3.

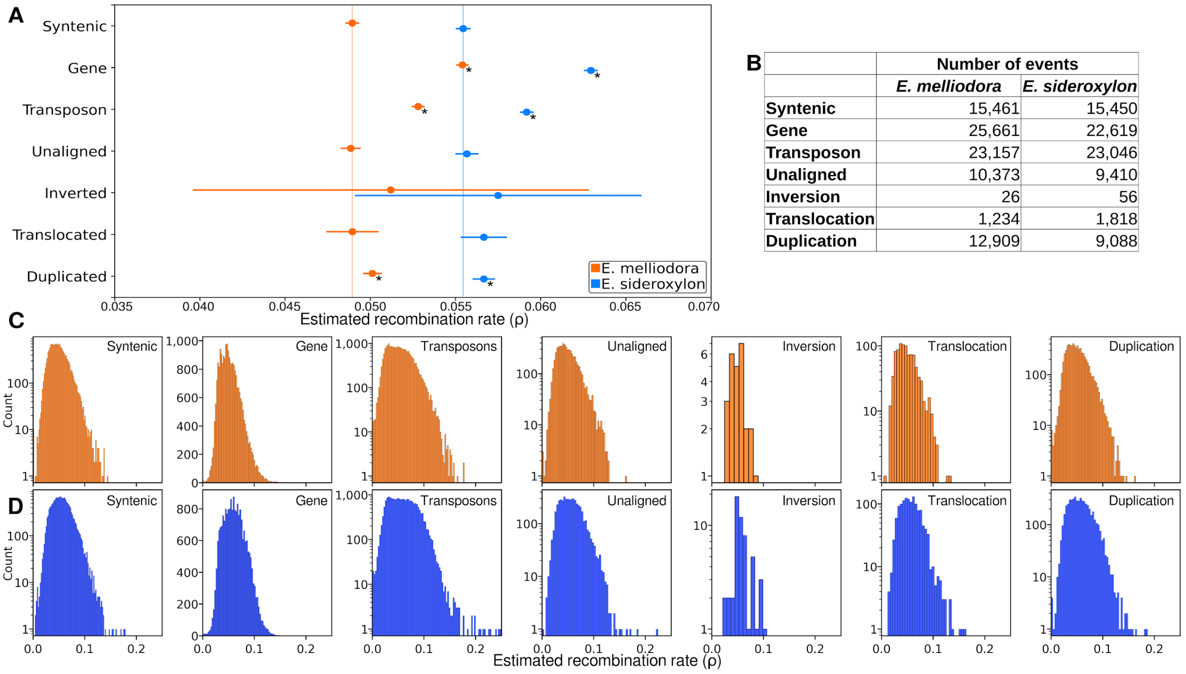

B

	Number of events	
	E. melliodora	***E. sideroxylon***
**Syntenic**	15,461	15,450
**Gene**	25,661	22,619
**Transposon**	23,157	23,046
**Unaligned**	10,373	9,410
**Inversion**	26	56
**Translocation**	1,234	1,818
**Duplication**	12,909	9,088

Figure 9. Tukey's test for estimated recombination rates of fixed SVs, unaligned regions, genes, and transposons. A) Shows mean and 95% confidence interval for all events. Vertical lines show average ρ for syntenic regions. * indicates region types that are significantly different from syntenic regions ($P \leq 0.05$). B) The number of events included in the analysis. C) Estimated recombination rate distribution for E. melliodora. D) Estimated recombination rate distribution for E. sideroxylon.

Effect of Synteny, rearranged, unaligned, and genes on Fixation index (F_{ST})

To measure the amount of shared genetic diversity that exists between *E. melliodora* and *E. sideroxylon*, we combined SNPs for both populations under each reference and calculated the fixation index (F_{ST}). The fixation index, calculated per SNP, scores the amount of genetic differentiation between populations or species and ranges from 0 to 1, where 0 indicates no difference in allele frequencies and 1 indicates a fixed difference. In real world usage, per SNP F_{ST} values are typically far below one, even in the case of isolated populations and should be interpreted relative to the study [69]. Here we use them to quantify how similar, or dissimilar, all region types are between *E. melliodora* and *E. sideroxylon*.

As per our examination of ρ, we calculated the average F_{ST} for all fixed SVs, and genes and transposons greater than 2 Kbp in length and performed Tukey's test, Figure 10A. Syntenic regions were used as the reference point to evaluate the extent of genetic differentiation of SVs. Using *E. melliodora* as the reference, all region types had significantly less divergence

between species except genes and inversions. Genes had significantly more divergence and inversions were sparse and as such had a wide confidence interval. A similar pattern was observed for *E. sideroxylon*. While genome-wide statistical observations of F_{ST} were unrevealing, many SVs were observed having F_{ST} less than the mean syntenic, Figure 10C and 10D. Examination of F_{ST} histograms for all event types showed a left shifted Poisson distribution, with many events having low F_{ST} scores. For detailed significance testing results refer to Supplementary Table S4.

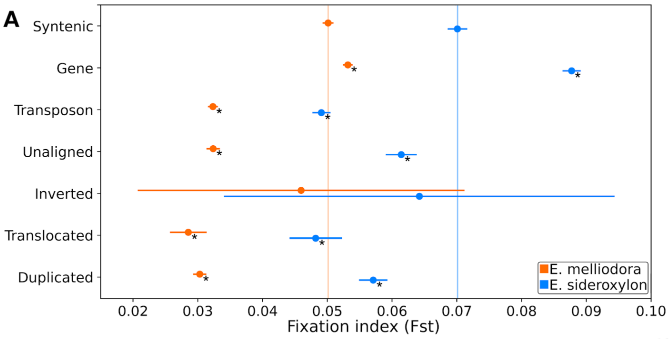

B

	Number of events	
	E. melliodora	***E. sideroxylon***
**Syntenic**	15,461	15,450
**Gene**	25,661	22,619
**Transposon**	23,157	23,046
**Unaligned**	10,373	9,410
**Inversion**	26	56
**Translocation**	1,234	1,818
**Duplication**	12,909	9,088

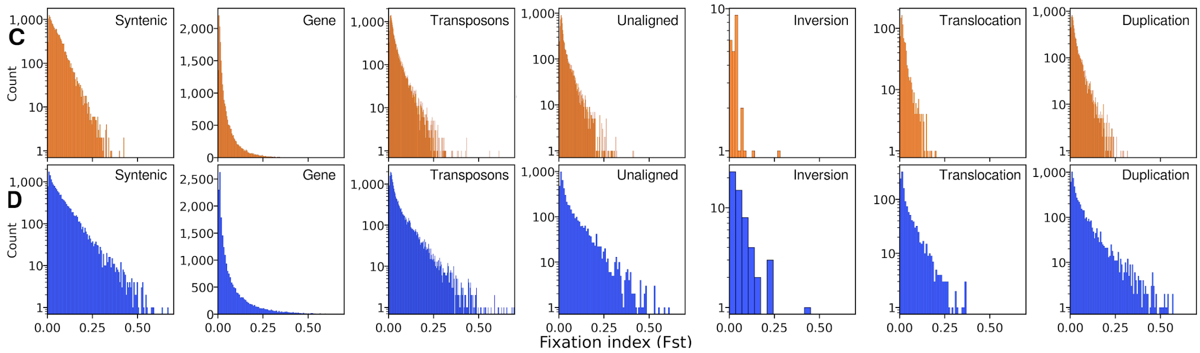

Figure 10. Tukey's test for Fixation index of fixed SVs, unaligned regions, genes, and transposons. A) Shows average FST and 95% confidence intervals calculated from average FST values for all regions. For each reference genome, SNPs from both species were combined and FST calculated. Vertical lines show average ρ for syntenic regions. * indicates region types that are significantly different from syntenic regions (P ≤ 0.05). B) The number of events included in the analysis. C) Fixation index distribution for E. melliodora. D) Fixation index distribution for E. sideroxylon.

Effect of Synteny, rearranged, unaligned, and genes on SNPs

SNP density can significantly impact the precision and resolution of both ρ and F_{ST} [70–72]. Higher SNP density enables finer-scale mapping of recombination events and more accurate population differentiation measurements, while lower SNP density gives coarser results with

reduced precision. Due to inconclusive results in both ρ and F_{ST} analyses, we examined SNP densities of SVs, genes, and TEs.

As per our ρ and F_{ST} analyses, we used Tukey's test and histograms to examine the differences in SNP densities for all fixed SVs, and genes and transposons greater than 2 Kbp in length, Figures 11A, 11C, and 11D. For detailed significance testing results refer to Supplementary Table S5. Reassuring to our SV annotation method, unaligned regions were the most diverged region type, containing the largest number of SNPs. Similarly reassuring for our annotation method, genes were the least diverged, containing the fewest SNPs. No significant correlations between the number of SNPs and ρ were observed. Notably, genes, transposons and duplications had high ρ, while only transposons had a high SNP density. Conversely, unaligned and translocated regions had low ρ, while only translocation had few SNPs. Similarly, no distinct correlations between SNPs and F_{ST} values were observed. Genes, despite having few SNPs, contained high F_{ST} values, whereas unaligned regions, with many SNPs, displayed low F_{ST} values. Translocated regions, with an intermediate number of SNPs, also exhibited low F_{ST} values. Although SNP densities contribute to the complex pattern of genomic differentiation, they showed no clear association with ρ and F_{ST} calculations.

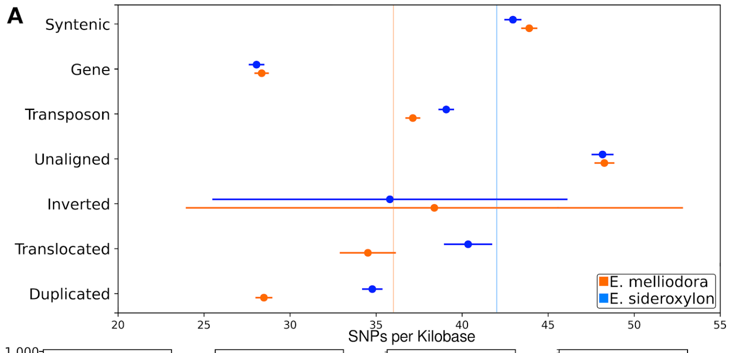

B

	Number of events	
	E. melliodora	*E. sideroxylon*
**Syntenic**	15,461	15,450
**Gene**	25,661	22,619
**Transposon**	23,157	23,046
**Unaligned**	10,373	9,410
**Inversion**	26	56
**Translocation**	1,234	1,818
**Duplication**	12,909	9,088

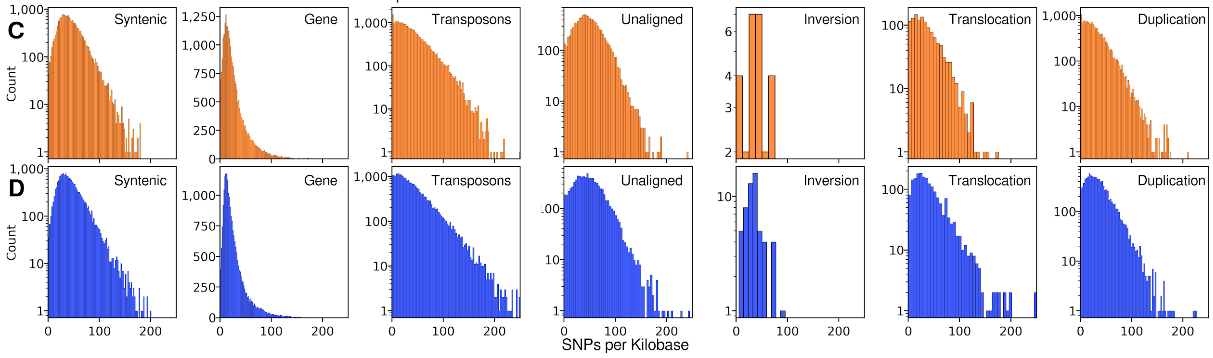

Figure 11. Tukey's test for SNP density of fixed SVs, unaligned regions, genes, and transposons. A) Shows mean and 95% confidence interval for all events. Vertical lines show average ρ for syntenic regions. B) The number of events included in the analysis. C) SNP density distribution for E. melliodora. D) SNP density distribution for E. sideroxylon.

Discussion

Structural variants are a major form of genomic variation, affecting more nucleotides than SNPs [73]. Despite their prominence, the functional and evolutionary impacts of SV's remain poorly understood [74–76]. To date, the majority of population-scale SV studies have focused on within population SV discovery and association with environments or phenotypes [77,78]. Several studies have also directly examined SV and their contribution to functional changes [79,80]. Here we genotyped interspecies SVs and described their frequencies within and among both species. Of particular novelty is our comparison of translocations and inversions, symmetric SVs that may be present within one or both species and at different frequencies. Between our recently diverged *Eucalyptus* species pair, our results demonstrate that SVs contribute to genome divergence, intra-species genetic diversity, and shared genetic diversity. Potentially of great interest are shared structural polymorphisms (SSP); these large mutations predate lineage divergence and remain polymorphic within both species, potentially containing locally adaptive or otherwise important genes and allele

combinations. Additionally, examination of average ρ and F_{ST} within fixed SVs demonstrates the variable effects of these genetic variations on genome differentiation and recombination.

Genetic mutations promote and reinforce lineage divergence, are the genetic basis of reproductive isolation, and are essential to the process of speciation. Structural mutations, by affecting recombination, phenotypes, or altering/removing/sub-functionalising genes, are of particular importance to speciation processes (Zhang et al., 2021). Barrier complexity and asymmetry are underappreciated components of reproductive isolation. Barrier complexity involves the combinatorial interplay of genetic barriers that collectively reduce reproductive success between individuals [81]. Successful offspring are survivors of genetic combinations, possessing genomes sufficiently free from barrier loci to allow reproduction to occur. Barrier asymmetry refers to the relative effectiveness of reproductive barriers between two groups, resulting in different hybridisation success rates [82]. *Eucalyptus melliodora* and *E. sideroxylon* are known to hybridise, and successful hybridisation likely results from the complex interplay between the numerous SD, SP, and SSP that come together in a particular hybrid. Evidence of linked SDs, SPs, and SSPs was observed within both *Eucalyptus* species. These linked SV combinations may be required for reproductive success or there could be some other fitness consequence that is maintaining selection for that linked state. Barrier SVs potentially exhibit a higher degree of reproductive isolation compared to non-SV regions, increasing genetic differentiation within these loci [83–85]. However, variation in F_{ST} did not provide sufficient evidence on average to support this conclusion, possibly due to the recent divergence of our species and the importance of only a few key interacting loci.

Similar to reproductive isolation, understanding how all types of genetic mutations contribute to the creation and maintenance of genetic diversity is crucial to understanding how organisms improve fitness and adapt to their changing environments [8,86,87]. Inversions and translocations aid in adaptive evolution by fixing allele combinations, duplications

contribute to the development of new genes, and insertions and deletions, often described as PAVs, modify gene expression and gene content [88–90]. A substantial number of inversions and translocations were successfully genotyped within both species. The majority of inversions and translocations were SP or SSP, making them candidates for exploring adaptive genes and alleles. Of particular note are SSP inversions and translocations which showed evidence of gene enrichment in potentially adaptive genes.

Duplications are known to be highly common and an important source of evolutionary novelty [91,92,92], and were the most common type of SV in our analysis. Most duplications were found to be fixed, with the remainder being almost entirely polymorphic. Given their asymmetry, duplications were genotyped only within their respective host genomes, resulting in an inability to categorise them as SD, SP, or SSP. Nonetheless, duplications successfully genotyped in our study are potential candidates for adaptive loci, likely having withstood the influences of genetic drift and purifying selection. Predicting the adaptive effects of unaligned regions presents a significant challenge, given their potential to encompass insertions, deletions, or highly divergent sequences. When unaligned regions result from highly divergent sequences, short reads will align poorly, confounding genotyping [66,67]. Genotyped as deletions, the majority of unaligned regions were fixed, and the remainder highly frequent. Fixed unaligned regions are possibly highly diverged regions or a deletion in the opposite species genome. When unaligned regions are polymorphic could represent insertions within the host species genome or deletions within the opposite species genome. These difficult to interpret regions may be PAVs, adaptive loci, or selectively neutral or deleterious loci undergoing potential purifying selection. Further investigations are essential to uncover their precise roles and implications.

It is now clear that SVs are of great evolutionary importance and must be considered when studying genetic diversity and genome evolution [90]. To better evaluate the impact of SVs on evolution a combination of interspecies and intraspecies studies are crucial. While

structural polymorphisms may be reproductive barriers or adaptive loci, they could also be neutral or deleterious, especially as these species separated very recently. Given that SVs are rarely conserved (i.e., typically purged over short time scales) [93,94], and many of the SV examined here were genotyped at high frequencies, there is potential for common SVs to be investigated for functional associations with traits or environments, thus warranting future scrutiny regarding their contribution to adaptive evolution. Future studies are needed to test whether these SVs contribute to adaptive evolution. To assess their potential role as barrier loci, breeding experiments could be employed. A problem encountered here was the number of SVs within individuals that could not be genotyped. Many statistical tests require all samples to be genotyped for all genetic variants, employing imputation to fill in missing genotypes. However, all current imputation processes are designed for SNPs captured within haplotype blocks. Statistical association programs that can incorporate SVs are needed. With the decreasing cost and increasing accuracy of long-read sequencing, particularly Oxford Nanopore (Ferguson et al., 2022), future studies could utilise high-throughput long-read sequencing to overcome the limitations of short-read SV genotyping. However advances in analysis software are still a limiting constraint for fully understanding the contribution of SVs to adaptive evolution and speciation.

Methods

Population sampling and sequencing

Yellow box (*Eucalyptus melliodora*) and red ironbark (*E. sideroxylon*) are closely-related eucalypts of the box-gum grassy woodland endangered ecological community. These species are often found growing in sympatry or parapatry, and widely hybridise throughout their ranges in southeastern Australia. We collected 472 *E. melliodora* and 180 *E. sideroxylon*, all samples being wild and undomesticated. Samples were environmentally stratified to capture major clines in climate-adaptive genomic variation across the species'

distributions. GPS data was recorded for each sample, and leaf material was dried in silica desiccant (Figure 4).

Twenty 3 mm disc punches (UniCore, Qiagen) from each leaf sample were placed in mini-tubes with a 3 mm ball bearing, frozen with liquid nitrogen and ground in a TissueLyser II (Qiagen). Genomic DNA was extracted using a 96-well plate column-based kit; Stratec Invisorb DNA Plant HTS 96 Kit/ C, according to the manufacturer's instructions (Stratec SE, Birkenfeld, Germany). DNA was quantified using a Infinite M1000 PRO Tecan fluorescence microplate reader (Tecan Trading AG, Switzerland), and standardised to 1 ng/µL, using a liquid handling robot. Library preparation was performed using a modified Illumina Nextera DNA Library Prep Kit workflow, which is available in Protocols.io and described in Jones et al [37]. Libraries were then quantified using GXII and Quant-iT, and pooled for equal representation. Prior to size selection, samples were concentrated using 2x binding buffer and 100 µL of Sera-Mag Speedbeads Carboxylate-Modified Particles (Thermo Scientific, Fremont, CA, USA). Size selection was then performed on a Pippin Prep (Sage Science, Inc., Beverly, MA, USA), for 400-650 bp fragments. Samples were again concentrated with 2x binding buffer and 100 µL of Sera-Mag beads, then quantified using both a Qubit Fluorometer (Thermo Scientific, Fremont, CA, USA) and Bioanalyzer high sensitivity DNA chips (Agilent Technologies, Santa Clara, CA, USA). Whole genome sequencing was performed on an Illumina NovaSeq 6000, 150 bp paired end sequencing, by Novogene (HK) Co., Ltd (Hong Kong).

Genome scaffolding

We performed Hi-C scaffolding, grouping, ordering, and orienting of our previously assembled *E. melliodora* genome into pseudo-chromosomes [29]. Leaves were obtained from the reference tree, and a proximity ligation library for chromosome conformation capture was created with a Phase Genomics Proximo Hi-C (Plant) Kit (version 4), according to the manufacturer's instructions (document KT3040B). DpnII, HinFI, MseI, DdeI to digest

the genome. Sequencing was performed on an Illumina NovaSeq 6000, 150 bp paired end sequencing. Hi-C scaffolding began by aligning all Hi-C reads to *E. melliodora's* contigs using bwa mem [38] (version: 0.7.17; parameters: -5SP). Next, PCR duplicates were identified with Samblaster [39] (version: 0.1.26). Linkage information captured within Hi-C reads was assessed with Juicer [40] (version: 1.6) and scaffolding was performed using 3D-DNA [41] (version: 190716; parameter: -i 1000). Due to the high repeat content, Hi-C read coverage was highly variable and resulted in poor quality scaffolding. To account for variability in read coverage, we ran 3D-DNA with "--editor-repeat-coverage 5", altering the misjoin detection threshold. After initial scaffolding the Hi-C contact map was manually edited with Juicebox [42] (version: 2.16). Previously assembled contigs for *E. sideroxylon* [29] were scaffolded with RagTag [43] (version: v2.1.0) using synteny to our Hi-C scaffolded *E. melliodora* genome.

Genome completeness was measured with BUSCO [44] (version 5) and long terminal repeat assembly index (Ou et al., 2018) (LAI). BUSCO scores genome completeness by identifying and reporting on the proportion of lineage specific highly conserved single-copy genes; more complete genomes have a high proportion of identified BUSCO genes. LAI identifies long terminal repeat (LTR) sequences and reports on the proportion that are intact; more complete genomes have a high proportion of intact LTR sequences.

Genome annotation

Genomes were annotated for transposable elements (TE) using genome-specific, *de novo* repeat libraries created with EDTA [46]; version: 1.9.6) and RepeatMasker [47] (version: 4.1.1). RepeatMasker additionally annotated our genomes for simple repeats. Repeat masked genomes were next annotated for genes using BRAKER2 [48] (version 2.1.6). BRAKER2 was run with ProtHint [49] (version2.6.0) and GeneMark-EP [49] (version: 4). ProtHint analysed training proteins to determine their evolutionary distance to the genome, aiding GeneMark-EP to train a gene detection model. Training protein sequences were

obtained from the National Center for Biotechnology Information (NCBI) [50] and included all available transcripts for Myrtaceae (Taxonomy ID: 3931) and *Arabidopsis thaliana* (Taxonomy ID: 3702).

Candidate genes were functionally annotated for eggNOG orthogroup, COG category, GO term, KEGG term, and PFAM using eggNOG-mapper [51] (version: 2.1.12; parameters: -m diamond --itype CDS --tax_scope Viridiplantae).

Synteny and structural variation annotation

Shared sequences were identified between genomes by alignment with nucmer (parameters: --maxmatch -l 40 -b 500 -c 200), from the MUMmer [52] (version: 3.23) toolset. Nucmer identifies all shared 40-mers between the two genomes and joins all 40-mers within 500 bp into single alignments. After aligning the two genomes MUMmer's delta-filter (parameters: -i 80 -l 200) tool removes all alignments < 200 bp and with an identity < 80%. A low sequence identity score (80%) was used due to the high heterozygosity of *Eucalyptus* genomes [53], and a higher score may incorrectly filter out real alignments. Using SyRI [54] (version: 1.5), filtered nucmer alignments were analysed and subsequently genomes were annotated for synteny, inverted, translocated, duplicated, and not-alignable regions.

All inversions, translocations, duplications, and unaligned regions described by SyRI were genotyped for all 563 samples within both species using Paragraph [55] and our short-read alignments.

A 0/1/2 matrix was created for all genotyped SV within both species and for all categories of SV. Using the R [56] function Cor, the correlation between SVs of interest was calculated and visualised with heatmap.

Alignment and variant calling

Raw population sequences were trimmed (sequencing adaptors and barcodes), quality filtered (average quality score < 20), and merged (overlapping read pairs were combined into single reads) using AdapterRemoval [57] (version: 2.3.0). Genome coverage was estimated for each sample and samples with low coverage (< 10x) were removed. Quality filtered reads were next aligned to both reference genomes (*E. melliodora* and *E. sideroxylon*) using bwa mem (parameters: -p). Samples with <75% alignment were then removed. Aligned reads for all remaining samples were variant called with bcftools [58] (version: 1.12) mpileup (parameters: MAPQ > 30, base quality > 15). The default mutation rate (0.0011) was increased to 0.01, making variant calling more robust when calling low coverage heterozygous SNPs. Variant files were then merged, resulting in four datasets; (reference genome - population species) *E. melliodora - E. melliodora*, *E. melliodora - E. sideroxylon*, *E. sideroxylon - E. melliodora*, and *E. sideroxylon - E. sideroxylon*.

Variant filtering

Using bcftools norm, multiallelic variants for each variant dataset were decomposed into multiple single variants. Decomposed variants were filtered, removing variants present in < 10% of samples and with less than 20 supporting reads, within each dataset using bcftools view. Variants were next recomposed, all remaining multiallelic variants rejoined, and each dataset further filtered to remove all indels and multiallelic SNPs [59].

High-quality, biallelic SNP datasets for each reference genome were combined, and a principal component analysis (PCA) performed with PCAngsd [60] (version: 1.10). Visual inspection of PCA plots allowed identification and removal of hybrids, outliers, and incorrectly labelled samples.

SNP phasing and recombination calculation

Before computing ρ (estimated recombination rate) within our four datasets, SNPs first required phasing. Phasing links each variant allele, placing them into haplotype blocks,

separating maternal and paternal variants. As the linkage information provided by paired-end short reads is not capable of phasing all SNPs, a two-step phasing process was used. First, individual samples were extracted from species variant files into a single sample variant file and using read alignments, SNPs, when possible, were phased with WhatsHap [61] (version: 1.7). Second, partially phased sample variant files were re-merged and the Hidden Markov Model (HMM) phaser SHAPEIT4 [62] (version: 4.2.2) inferred haplotypes and phased the remaining unphased SNPs. Parameters (--use-PS 0.0001 --mcmc-iterations 6b,1p,1b,1p,1b,1p,1b,1p,8m --pbwt-depth 6 --sequencing) specified for SHAPEIT4 were optimised by balancing maximum accuracy and runtime. At the completion of this two-stage phasing approach all SNPs for each dataset were phased. After phasing, ρ was calculated for each dataset using LDJump [63] (parameters: alpha = 0.05; version: 0.3.1), specifying a window size of 1 kb. LDJump made use of LDHat [64] (version: 2.2a) to decrease runtime.

Fixation index (F_{ST})

Filtered SNP datasets were combined for each reference genome, and subsequently F_{ST} was calculated for each SNP using PLINK [65] (version: 1.9). Per SNP F_{ST} values were averaged for each region of interest for further analysis.

Data access

Sequencing data and reference genomes generated in this project are publicly available on the Sequence Read Archive (SRA) and NCBI genome repository under BioProject PRJNA509734 and XXX. Gene predictions and repeat annotations have been deposited in FigShare and are available at: XXX. All analysis scripts created and used by this project have been deposited within our github repository: https://github.com/fergsc/Polymorphic-interspecies-SVs.

Competing interest statement

The authors declare that they have no competing interests.

Funding

This work was supported by the Australian Research Council (CE140100008; DP150103591; DE190100326) and an Australian Government Research Training Program scholarship.

Authors' contributions

Scott Ferguson led the project and ran all the analysis. The project was conceived and designed by all authors. Scott Ferguson wrote the first manuscript draft. All authors contributed to writing and review of the final manuscript.

Acknowledgements

This research was undertaken with the assistance of resources from the National Computational Infrastructure (NCI Australia), an NCRIS enabled capability supported by the Australian Government.

We would like to thank the Australian National Botanic Gardens in Canberra, Australia for providing plant samples and associated metadata for the two reference genomes, *E. melliodora* and *E. sideroxylon*. This research acknowledges the support provided by the Director of National Parks, the park staff of the Australian National Botanic Gardens, and Parks Australia. The views expressed in this document do not necessarily represent the views of the Australian Government.

We thank David Stanley and Cynthia Torkel for their technical assistance in the laboratory and their friendship throughout the times.

References

1. Alonge M, Wang X, Benoit M, Soyk S, Pereira L, Zhang L, et al.. Major Impacts of Widespread Structural Variation on Gene Expression and Crop Improvement in Tomato. *Cell*. 2020; doi: 10.1016/j.cell.2020.05.021.

2. Imprialou M, Kahles A, Steffen JG, Osborne EJ, Gan X, Lempe J, et al.. Genomic Rearrangements in Arabidopsis Considered as Quantitative Traits. *Genetics*. 2017; doi: 10.1534/genetics.116.192823.

3. Weischenfeldt J, Symmons O, Spitz F, Korbel JO. Phenotypic impact of genomic structural variation: insights from and for human disease. *Nat Rev Genet*. 2013; doi: 10.1038/nrg3373.

4. Marques DA, Meier JI, Seehausen O. A Combinatorial View on Speciation and Adaptive Radiation. *Trends Ecol Evol*. 2019; doi: 10.1016/j.tree.2019.02.008.

5. Zhang L, Reifová R, Halenková Z, Gompert Z. How Important Are Structural Variants for Speciation? *Genes*. 2021; doi: 10.3390/genes12071084.

6. Savocco J, Piazza A. Recombination-mediated genome rearrangements. *Curr Opin Genet Dev*. 71:92021;

7. Sedlazeck FJ, Rescheneder P, Smolka M, Fang H, Nattestad M, von Haeseler A, et al.. Accurate detection of complex structural variations using single-molecule sequencing. *Nat Methods*. 2018; doi: 10.1038/s41592-018-0001-7.

8. Pokrovac I, Pezer Ž. Recent advances and current challenges in population genomics of structural variation in animals and plants. *Front Genet*. 132022;

9. Radke DW, Lee C. Adaptive potential of genomic structural variation in human and mammalian evolution. *Brief Funct Genomics*. 2015; doi: 10.1093/bfgp/elv019.

10. Stewart NB, Rogers RL. Chromosomal rearrangements as a source of new gene formation in Drosophila yakuba. Malik HS, editor. *PLOS Genet*. 2019; doi: 10.1371/journal.pgen.1008314.

11. Kim K, Eom J, Jung I. Characterization of Structural Variations in the Context of 3D Chromatin Structure. *Mol Cells*. 2019; doi: 10.14348/molcells.2019.0137.

12. Shanta O, Noor A, Chaisson MJP, Sanders AD, Zhao X, Malhotra A, et al.. The effects of common structural variants on 3D chromatin structure. *BMC Genomics*. 2020; doi: 10.1186/s12864-020-6516-1.

13. Thompson MJ, Jiggins CD. Supergenes and their role in evolution. *Heredity*. 2014; doi: 10.1038/hdy.2014.20.

14. Kirkpatrick M, Barton N. Chromosome Inversions, Local Adaptation and Speciation. *Genetics*. 2006; doi: 10.1534/genetics.105.047985.

15. Lande R. The fixation of chromosomal rearrangements in a subdivided population with

local extinction and colonization. *Heredity*. Nature Publishing Group; 1985; doi: 10.1038/hdy.1985.43.

16. Walsh JB. Rate of Accumulation of Reproductive Isolation by Chromosome Rearrangements. *Am Nat*. The University of Chicago Press; 1982; doi: 10.1086/284008.

17. Rieseberg LH. Chromosomal rearrangements and speciation. :82001;

18. Harringmeyer OS, Hoekstra HE. Chromosomal inversion polymorphisms shape the genomic landscape of deer mice. *Nat Ecol Evol*. Nature Publishing Group; 2022; doi: 10.1038/s41559-022-01890-0.

19. Robberecht C, Voet T, Esteki MZ, Nowakowska BA, Vermeesch JR. Nonallelic homologous recombination between retrotransposable elements is a driver of de novo unbalanced translocations. *Genome Res*. 2013; doi: 10.1101/gr.145631.112.

20. Ortiz-Barrientos D, Engelstädter J, Rieseberg LH. Recombination Rate Evolution and the Origin of Species. *Trends Ecol Evol*. 2016; doi: 10.1016/j.tree.2015.12.016.

21. Flagel LE, Wendel JF. Gene duplication and evolutionary novelty in plants. *New Phytol*. 2009; doi: 10.1111/j.1469-8137.2009.02923.x.

22. Wu B, Cox MP. Greater genetic and regulatory plasticity of retained duplicates in Epichloë endophytic fungi. *Mol Ecol*. 2019; doi: 10.1111/mec.15275.

23. Braasch I, Gehrke AR, Smith JJ, Kawasaki K, Manousaki T, Pasquier J, et al.. The spotted gar genome illuminates vertebrate evolution and facilitates human-teleost comparisons. *Nat Genet*. Nature Publishing Group; 2016; doi: 10.1038/ng.3526.

24. Freeling M, Scanlon MJ, Fowler JE. Fractionation and subfunctionalization following genome duplications: mechanisms that drive gene content and their consequences. *Curr Opin Genet Dev*. 2015; doi: 10.1016/j.gde.2015.11.002.

25. Lien S, Koop BF, Sandve SR, Miller JR, Kent MP, Nome T, et al.. The Atlantic salmon genome provides insights into rediploidization. *Nature*. Nature Publishing Group; 2016; doi: 10.1038/nature17164.

26. Conrad DF, Hurles ME. The population genetics of structural variation. *Nat Genet*. 2007; doi: 10.1038/ng2042.

27. Sun Y, Wang J, Li Y, Jiang B, Wang X, Xu W-H, et al.. Pan-Genome Analysis Reveals the Abundant Gene Presence/Absence Variations Among Different Varieties of Melon and Their Influence on Traits. *Front Plant Sci*. 132022;

28. Yuan Y, Bayer PE, Batley J, Edwards D. Current status of structural variation studies in plants. *Plant Biotechnol J*. 2021; doi: 10.1111/pbi.13646.

29. Ferguson S, Jones A, Murray K, Schwessinger B, Borevitz JO. Interspecies genome divergence is predominantly due to frequent small scale rearrangements in Eucalyptus. *Mol Ecol*. 2022; doi: 10.1111/mec.16608.

30. Hejase HA, Salman-Minkov A, Campagna L, Hubisz MJ, Lovette IJ, Gronau I, et al.. Genomic islands of differentiation in a rapid avian radiation have been driven by recent selective sweeps. *Proc Natl Acad Sci*. Proceedings of the National Academy of Sciences; 2020; doi: 10.1073/pnas.2015987117.

31. Eshel G, Araus V, Undurraga S, Soto DC, Moraga C, Montecinos A, et al.. Plant ecological genomics at the limits of life in the Atacama Desert. *Proc Natl Acad Sci*. Proceedings of the National Academy of Sciences; 2021; doi: 10.1073/pnas.2101177118.

32. Henderson EC, Brelsford A. Genomic differentiation across the speciation continuum in three hummingbird species pairs. *BMC Evol Biol*. 2020; doi: 10.1186/s12862-020-01674-9.

33. Piatkowski B, Weston DJ, Aguero B, Duffy A, Imwattana K, Healey AL, et al.. Divergent selection and climate adaptation fuel genomic differentiation between sister species of Sphagnum (peat moss). *Ann Bot*. 2023; doi: 10.1093/aob/mcad104.

34. Zhang J, Zhang S, Zheng Z, Lu Z, Yang Y. Genomic divergence between two sister Ostrya species through linked selection and recombination. *Ecol Evol*. 2022; doi: 10.1002/ece3.9611.

35. Ferguson S, Jones A, Murray K, Andrew R, Schwessinger B, Borevitz J. Plant genome evolution in the genus Eucalyptus driven by structural rearrangements that promote sequence divergence. *bioRxiv*. 2023; doi: 10.1101/2023.04.19.537464.

36. Thornhill AH, Crisp MD, Külheim C, Lam KE, Nelson LA, Yeates DK, et al.. A dated molecular perspective of eucalypt taxonomy, evolution and diversification. *Aust Syst Bot*. 2019; doi: 10.1071/SB18015.

37. Jones A, Stanley D, Ferguson S, Schwessinger B, Borevitz J, Warthmann N. Cost-conscious generation of multiplexed short-read DNA libraries for whole-genome sequencing. *PLOS ONE*. Public Library of Science; 2023; doi: 10.1371/journal.pone.0280004.

38. Li H. Aligning sequence reads, clone sequences and assembly contigs with BWA-MEM. *ArXiv13033997 Q-Bio*. 2013;

39. Faust GG, Hall IM. SAMBLASTER: fast duplicate marking and structural variant read extraction. *Bioinformatics*. 2014; doi: 10.1093/bioinformatics/btu314.

40. Durand NC, Shamim MS, Machol I, Rao SSP, Huntley MH, Lander ES, et al.. Juicer Provides a One-Click System for Analyzing Loop-Resolution Hi-C Experiments. *Cell Syst*. 2016; doi: 10.1016/j.cels.2016.07.002.

41. Dudchenko O, Batra SS, Omer AD, Nyquist SK, Hoeger M, Durand NC, et al.. De novo assembly of the Aedes aegypti genome using Hi-C yields chromosome-length scaffolds. *Science*. American Association for the Advancement of Science; 2017; doi: 10.1126/science.aal3327.

42. Durand NC, Robinson JT, Shamim MS, Machol I, Mesirov JP, Lander ES, et al.. Juicebox Provides a Visualization System for Hi-C Contact Maps with Unlimited Zoom. *Cell Syst*. Elsevier; 2016; doi: 10.1016/j.cels.2015.07.012.

43. Alonge M, Lebeigle L, Kirsche M, Jenike K, Ou S, Aganezov S, et al.. Automated assembly scaffolding using RagTag elevates a new tomato system for high-throughput genome editing. *Genome Biol*. 2022; doi: 10.1186/s13059-022-02823-7.

44. Manni M, Berkeley MR, Seppey M, Simão FA, Zdobnov EM. BUSCO Update: Novel and Streamlined Workflows along with Broader and Deeper Phylogenetic Coverage for Scoring of Eukaryotic, Prokaryotic, and Viral Genomes. Kelley J, editor. *Mol Biol Evol*. 2021; doi: 10.1093/molbev/msab199.

45. Ou S, Chen J, Jiang N. Assessing genome assembly quality using the LTR Assembly Index (LAI). *Nucleic Acids Res*. 2018; doi: 10.1093/nar/gky730.

46. Ou S, Su W, Liao Y, Chougule K, Agda JRA, Hellinga AJ, et al.. Benchmarking transposable element annotation methods for creation of a streamlined, comprehensive pipeline. *Genome Biol*. 2019; doi: 10.1186/s13059-019-1905-y.

47. Smit A, Hubley R, Green P: RepeatMasker Open-4.0. <http://www.repeatmasker.org> (2020). Accessed 2020 Feb 11.

48. Brůna T, Hoff KJ, Lomsadze A, Stanke M, Borodovsky M. BRAKER2: automatic eukaryotic genome annotation with GeneMark-EP+ and AUGUSTUS supported by a protein database. *NAR Genomics Bioinforma*. 2021; doi: 10.1093/nargab/lqaa108.

49. Brůna T, Lomsadze A, Borodovsky M. GeneMark-EP+: eukaryotic gene prediction with self-training in the space of genes and proteins. *NAR Genomics Bioinforma*. 2020; doi: 10.1093/nargab/lqaa026.

50. Sayers EW, Beck J, Bolton EE, Bourexis D, Brister JR, Canese K, et al.. Database resources of the National Center for Biotechnology Information. *Nucleic Acids Res*. 2021; doi: 10.1093/nar/gkaa892.

51. Cantalapiedra CP, Hernández-Plaza A, Letunic I, Bork P, Huerta-Cepas J. eggNOG-mapper v2: Functional Annotation, Orthology Assignments, and Domain Prediction at the Metagenomic Scale. *Mol Biol Evol*. 2021; doi: 10.1093/molbev/msab293.

52. Kurtz S, Phillippy A, Delcher AL, Smoot M, Shumway M, Antonescu C, et al.. Versatile and open software for comparing large genomes. *Genome Biol*. :9 2004;

53. Murray KD, Janes JK, Jones A, Bothwell HM, Andrew RL, Borevitz JO. Landscape drivers of genomic diversity and divergence in woodland Eucalyptus. *Mol Ecol*. 2019; doi: 10.1111/mec.15287.

54. Goel M, Sun H, Jiao W-B, Schneeberger K. SyRI: finding genomic rearrangements and local sequence differences from whole-genome assemblies. *Genome Biol*. 2019; doi: 10.1186/s13059-019-1911-0.

55. Chen S, Krusche P, Dolzhenko E, Sherman RM, Petrovski R, Schlesinger F, et al.. Paragraph: a graph-based structural variant genotyper for short-read sequence data. *Genome Biol*. 2019; doi: 10.1186/s13059-019-1909-7.

56. R Core Team. R: A Language and Environment for Statistical Computing. Vienna, Austria: R Foundation for Statistical Computing;

57. Schubert M, Lindgreen S, Orlando L. AdapterRemoval v2: rapid adapter trimming, identification, and read merging. *BMC Res Notes*. 2016; doi: 10.1186/s13104-016-1900-2.

58. Danecek P, Bonfield JK, Liddle J, Marshall J, Ohan V, Pollard MO, et al.. Twelve years of SAMtools and BCFtools. *GigaScience*. 2021; doi: 10.1093/gigascience/giab008.

59. Murray K. kdm9/Acanthophis: Version 0.2.0. Zenodo;

60. Meisner J, Albrechtsen A. Inferring Population Structure and Admixture Proportions in Low-Depth NGS Data. *Genetics*. 2018; doi: 10.1534/genetics.118.301336.

61. Martin M, Patterson M, Garg S, O Fischer S, Pisanti N, Klau GW, et al.. WhatsHap: fast and accurate read-based phasing. Bioinformatics; 2016 Nov.

62. Delaneau O, Zagury J-F, Robinson MR, Marchini JL, Dermitzakis ET. Accurate, scalable and integrative haplotype estimation. *Nat Commun*. Nature Publishing Group; 2019; doi: 10.1038/s41467-019-13225-y.

63. Hermann P, Heissl A, Tiemann-Boege I, Futschik A. LDJump: Estimating variable recombination rates from population genetic data. *Mol Ecol Resour*. 2019; doi: 10.1111/1755-0998.12994.

64. Auton A, McVean G. Recombination rate estimation in the presence of hotspots. *Genome Res*. 2007; doi: 10.1101/gr.6386707.

65. Chang CC, Chow CC, Tellier LC, Vattikuti S, Purcell SM, Lee JJ. Second-generation PLINK: rising to the challenge of larger and richer datasets. *GigaScience*. 2015; doi: 10.1186/s13742-015-0047-8.

66. Alser M, Rotman J, Deshpande D, Taraszka K, Shi H, Baykal PI, et al.. Technology dictates algorithms: recent developments in read alignment. *Genome Biol*. 2021; doi: 10.1186/s13059-021-02443-7.

67. Valiente-Mullor C, Beamud B, Ansari I, Francés-Cuesta C, García-González N, Mejía L, et al.. One is not enough: On the effects of reference genome for the mapping and subsequent analyses of short-reads. *PLOS Comput Biol*. Public Library of Science; 2021; doi: 10.1371/journal.pcbi.1008678.

68. Galperin MY, Wolf YI, Makarova KS, Vera Alvarez R, Landsman D, Koonin EV. COG database update: focus on microbial diversity, model organisms, and widespread pathogens. *Nucleic Acids Res*. 2020; doi: 10.1093/nar/gkaa1018.

69. Kitada S, Nakamichi R, Kishino H. Understanding population structure in an evolutionary context: population-specific *F* ST and pairwise *F* ST. Ross-Ibarra J, editor. *G3 GenesGenomesGenetics*. 2021; doi: 10.1093/g3journal/jkab316.

70. Akey JM, Zhang G, Zhang K, Jin L, Shriver MD. Interrogating a High-Density SNP Map for Signatures of Natural Selection. *Genome Res*. 2002; doi: 10.1101/gr.631202.

71. Bhatia G, Patterson N, Sankararaman S, Price AL. Estimating and interpreting FST: The impact of rare variants. *Genome Res*. 2013; doi: 10.1101/gr.154831.113.

72. Chan AH, Jenkins PA, Song YS. Genome-Wide Fine-Scale Recombination Rate Variation in Drosophila melanogaster. *PLOS Genet*. Public Library of Science; 2012; doi: 10.1371/journal.pgen.1003090.

73. Escaramís G, Docampo E, Rabionet R. A decade of structural variants: description, history and methods to detect structural variation. *Brief Funct Genomics*. 2015; doi: 10.1093/bfgp/elv014.

74. Chain FJJ, Feulner PGD. Ecological and evolutionary implications of genomic structural variations. *Front Genet*. 2014; doi: 10.3389/fgene.2014.00326.

75. Ho SS, Urban AE, Mills RE. Structural variation in the sequencing era. *Nat Rev Genet*. Nature Publishing Group; 2020; doi: 10.1038/s41576-019-0180-9.

76. Yan SM, Sherman RM, Taylor DJ, Nair DR, Bortvin AN, Schatz MC, et al.. Local adaptation and archaic introgression shape global diversity at human structural variant loci. Perry GH, editor. *eLife*. eLife Sciences Publications, Ltd; 2021; doi: 10.7554/eLife.67615.

77. Gui S, Wei W, Jiang C, Luo J, Chen L, Wu S, et al.. A pan-Zea genome map for enhancing maize improvement. *Genome Biol*. 2022; doi: 10.1186/s13059-022-02742-7.

78. Hufford MB, Seetharam AS, Woodhouse MR, Chougule KM, Ou S, Liu J, et al.. De novo assembly, annotation, and comparative analysis of 26 diverse maize genomes. :92021;

79. Ishikawa A, Kabeya N, Ikeya K, Kakioka R, Cech JN, Osada N, et al.. A key metabolic gene for recurrent freshwater colonization and radiation in fishes. *Science*. American Association for the Advancement of Science; 2019; doi: 10.1126/science.aau5656.

80. Zhao Y, Long L, Wan J, Biliya S, Brady SC, Lee D, et al.. A spontaneous complex

structural variant in rcan-1 increases exploratory behavior and laboratory fitness of Caenorhabditis elegans. *PLOS Genet*. Public Library of Science; 2020; doi: 10.1371/journal.pgen.1008606.

81. Shang H, Hess J, Pickup M, Field DL, Ingvarsson PK, Liu J, et al.. Evolution of strong reproductive isolation in plants: broad-scale patterns and lessons from a perennial model group. *Philos Trans R Soc B Biol Sci*. Royal Society; 2020; doi: 10.1098/rstb.2019.0544.

82. Christie K, Fraser LS, Lowry DB. The strength of reproductive isolating barriers in seed plants: Insights from studies quantifying premating and postmating reproductive barriers over the past 15 years. *Evolution*. 2022; doi: 10.1111/evo.14565.

83. Berg PR, Star B, Pampoulie C, Sodeland M, Barth JMI, Knutsen H, et al.. Three chromosomal rearrangements promote genomic divergence between migratory and stationary ecotypes of Atlantic cod. *Sci Rep*. Nature Publishing Group; 2016; doi: 10.1038/srep23246.

84. Huang K, Andrew RL, Owens GL, Ostevik KL, Rieseberg LH. Multiple chromosomal inversions contribute to adaptive divergence of a dune sunflower ecotype. *Mol Ecol*. 2020; doi: 10.1111/mec.15428.

85. Lucek K, Gompert Z, Nosil P. The role of structural genomic variants in population differentiation and ecotype formation in Timema cristinae walking sticks. *Mol Ecol*. 2019; doi: 10.1111/mec.15016.

86. Gregory TR. Understanding Natural Selection: Essential Concepts and Common Misconceptions. *Evol Educ Outreach*. 2009; doi: 10.1007/s12052-009-0128-1.

87. Loewe L, Hill WG. The population genetics of mutations: good, bad and indifferent. *Philos Trans R Soc B Biol Sci*. 2010; doi: 10.1098/rstb.2009.0317.

88. De Oliveira R, Rimbert H, Balfourier F, Kitt J, Dynomant E, Vrána J, et al.. Structural Variations Affecting Genes and Transposable Elements of Chromosome 3B in Wheats. *Front Genet*. 112020;

89. Mérot C, Oomen RA, Tigano A, Wellenreuther M. A Roadmap for Understanding the Evolutionary Significance of Structural Genomic Variation. *Trends Ecol Evol*. 2020; doi: 10.1016/j.tree.2020.03.002.

90. Wellenreuther M, Mérot C, Berdan E, Bernatchez L. Going beyond SNPs: The role of structural genomic variants in adaptive evolution and species diversification. *Mol Ecol*. 2019; doi: 10.1111/mec.15066.

91. Cohen ZP, Schoville SD, Hawthorne DJ. The role of structural variants in pest adaptation and genome evolution of the Colorado potato beetle, Leptinotarsa decemlineata (Say). *Mol Ecol*. 2023; doi: 10.1111/mec.16838.

92. Hanada K, Zou C, Lehti-Shiu MD, Shinozaki K, Shiu S-H. Importance of lineage-specific expansion of plant tandem duplicates in the adaptive response to environmental stimuli. *Plant Physiol*. 2008; doi: 10.1104/pp.108.122457.

93. Inoue J, Sato Y, Sinclair R, Tsukamoto K, Nishida M. Rapid genome reshaping by multiple-gene loss after whole-genome duplication in teleost fish suggested by mathematical modeling. *Proc Natl Acad Sci*. Proceedings of the National Academy of Sciences; 2015; doi: 10.1073/pnas.1507669112.

94. Naseeb S, Ames RM, Delneri D, Lovell SC. Rapid functional and evolutionary changes follow gene duplication in yeast. *Proc R Soc B Biol Sci*. Royal Society; 2017; doi:

10.1098/rspb.2017.1393.

95. Ferguson S, McLay T, Andrew RL, Bruhl JJ, Schwessinger B, Borevitz J, et al.. Species-specific basecallers improve actual accuracy of nanopore sequencing in plants. *Plant Methods*. 2022; doi: 10.1186/s13007-022-00971-2.

Supplementary file

Click here to access/download
Supplementary Material
Supps.pdf
