# Supplementary material for: Exploring the role of polymorphic interspecies structural variants in reproductive isolation and adaptive divergence in Eucalyptus
Source: Gigascience. 2024 Jun 13;13:giae029. doi: 10.1093/gigascience/giae029 (PMC11170218; doi:10.1093/gigascience/giae029)
Supplement: giae029_Response_to_Reviewer_Comments_Original_Submission [file giae029_response_to_reviewer_comments_original_submission.pdf]

Author's Response To Reviewer Comments

Dear Editor and reviewers,

We would like to extend a warm thank you for taking the time to review our manuscript, identify the positive values in our re
entitled "Exploring polymorphic interspecies structural variants in Eucalyptus: Unravelling Their Role in Reproductive Isolation
Kevin Murray, Rose L. Andrew, Benjamin Schwessinger, Helen Bothwell, and Justin Borevitz, for consideration for publication

As a result of the review, we have altered our manuscript, carefully considering the comments by all reviewers. All comments
explanation of how we have addressed each comment and describing the changes made to the manuscript. During the review
manuscript, making it shorter and more concise. The new title is "Exploring the role of polymorphic interspecies structural va
Eucalyptus"

A revised manuscript has been submitted with changes highlighted in yellow. All data generated and scripts created during th
repository, as detailed in our "Data availability" section.

Yours sincerely,

Scott Ferguson, on behalf of all authors.

Reviewer #1:
Ferguson et al have performed a thorough analysis of two species of Eucalyptus, quantifying the extent of structural variation
how prevalent those variations are across a selection of wild material. I believe this study is of sufficient quality for publicatio
grammatical issues are addressed, and a few supporting analyses are performed.

The major changes I would like to see include the addition of a syri plot of the complete set of SVs between E. melliodora and
on the plots of recombination in Figure S6/S7 would allow for a better comparison of how recombination rate is interacting wi
enrichment for COG terms, to better support the statements of "enrichment" in the discussion.
We thank the reviewer for their efforts in reviewing our manuscript and seeing the quality of our work. In response to this rev
test that found 51 GO terms (shared: 31; E. melliodora: 11; E. sideroxylon: 9) to be significantly less represented in shared s
terms found to be significantly higher. These GO terms were associated with biological process, cellular component, and mole
Supplementary Figures, and added a SyRI plot. We have addressed and incorporated all of Reviewer 1's comments extensive

Suggested changes by line:

Line 142 - This section is quite short, I would either merge this section into the Genome scaffolding (and annotation) section,
We agree that this section is very short, and have combined it with genome scaffolding. This has improved the flow of inform

Line 182 - (Supplementary Figure S4)
"Figures" has been changed to "Figure"

Line 183 (and throughout) - Please be consistent with your references to tables and figures.
We have revised our manuscript and ensured all references to tables and figures are consistent. Multiple instances were foun
manuscript

Line 186 - delete comma after 28.63%
Comma removed.

Line 194 - These are density plots rather than histograms
Histogram has been corrected to density.

Figure 4 - Both axes are labelled as PC1
Y-axis has been corrected and now reads PC2.

Line 217 (page 10, line numbers are doubled up) - This seems repetitive, perhaps "…especially as they may also represent di
Modified, from the original:
"Additionally, genotyping unaligned regions as insertions or deletions introduces uncertainties, especially as they may represe
To an improved:
"Genotyping unaligned regions introduces uncertainties, especially as they may represent insertions, deletions, or divergent s

Line 221 (page 11) - Please insert "and" before polymorphic translocations
We have added "and" into this sentence.
It now reads as:
"To categorise symmetric interspecies SVs as SD, SP, or SSP we combined the status of fixed inversions (E. melliodora: 130;
66; E. sideroxylon: 37), fixed translocations (E. melliodora: 5,652; E. sideroxylon: 6,634), and polymorphic translocations (E

Line 223 - You have stated that those not successfully genotyped in both species are private or artefacts earlier in the paragr
Changed:
"The analysis revealed that the majority of inversions and translocations were either fixed in both species or not successfully
reference genome or assembly/scaffolding artefacts."
To
"The analysis revealed that the majority of inversions and translocations were either fixed in both species or not successfully

Figure 6 - I don't find this figure particularly informative (and somewhat confusing to interpret). I think showing the percenta
equivalence in genomic impact, which is difficult to reconcile with the raw difference in numbers. I think a supplemental table
better.
Figure 6 has been replaced with Table 4. Table 4 lists the number and percent of events classified as SD, SP, and SSP. Additi
on inversion and translocation classifications. This has improved the readability and interpretability of these complex findings

Accordingly, manuscript text has changed from:
"The remaining proportion consisted of SPs (inversions: 25.98%, translocations: 24.80%) or SSPs (inversions: 7.79%, transl
To:
"The remaining proportion consisted of SPs or SSPs, and a small number of SD. For details on inversion and translocation clas
classification, see Supplementary Table S2."

Line 246 - There is no mention in the methods about what r threshold was used to declare a pair "correlated", please state it
Per the reviewer's request, we have modified this section of the manuscript. Figure 7 now includes a legend for each correlati
text in results has been modified to indicate what R2 threshold we used. We also took this opportunity to make our threshold

Line 265 - This line was confusing to interpret. A suggested alteration: "significant value. After attempting to functionally ann
COG categories, 247 of the total 281 gene candidates in SSPs were annotated. These genes were enriched for...."
We thank the reviewer for their suggestion and have incorporated it as follows, changing the original:
"Examination of SSPs identified 281 gene candidates (E. melliodora: 145 and E. sideroxylon: 136), of which 247 (87%; E. me
annotated into eggNOG orthogroups and grouped into COG (Clusters of Orthologous Groups) [39] categories, Figure 8. Simila
COG categories. Comparing all genes to SSP genes indicates that SSP genes have an increased association with DNA replicati
modification, protein turnover, chaperones, signal transduction, intercellular communication, and unexplored aspects of biolo
with categories for fundamental cellular functions, such as protein synthesis, defence against pathogens, maintaining cellular
molecular processes involving amino acids, nucleotides, and coenzymes."
To an updated:
"After attempting to functionally annotate all genes across the genome and placing them within COG categories [39], 247 of
Figure 8). These genes were enriched for DNA replication, DNA recombination, DNA repair, post-translational modification, pr
communication, and unexplored aspects of biology. SSP genes were found to be underrepresented in categories related to fu
against pathogens, maintaining cellular integrity, providing structural support, and regulating crucial molecular processes inv

Line 266 - I would like to see a formal enrichment analysis rather than "increased/decreased association", so we could have a
represented in SSPs. You could subsequently limit Figure 8 to those that show a difference.
We agree that is an appropriate analysis to add and have performed and included a GO term enrichment analysis. The manus
following text has been added:

Methods:
“Gene Ontology (GO) terms were extracted from all eggNOG annotated genes and a GO term enrichment analysis performed
[89] (version: 1.3.11).”

Results:
“Additionally, we performed a Gene Ontology (GO) [42] enrichment test for all genes identified in SSPs. We found 51 GO terr
significantly less represented in SSP genes compared to all genes, with no GO terms found to be significantly higher. Gene Or
cellular component (15), and molecular function (8). Further details can be found in Supplementary Table S3.”

Line 275 - The grammar of this title is a bit off, perhaps "Effect of syntenic, rearranged, unaligned regions and genes on reco
We have incorporated the reviewer's suggestion verbatim, changing the title from:
“Effect of Synteny, rearranged, unaligned, and genes on recombination rates”
To
“Effect of syntenic, rearranged, unaligned regions and genes on recombination rate (ρ)”

Line 276 - This is the first mention of p, please define it as recombination rate
Added ρ definition to introduction text, as follows in the improved manuscript.

Changed
“Structural variation rates are compared to find evidence of SD, SP, and SSP. Additionally, we examine recombination rates a
allele fixation and accelerated evolution between populations.”
To
“Structural variation rates are compared to find evidence of SD, SP, and SSP. Additionally, we examine recombination rates (
assess allele fixation and accelerated evolution between populations.”

Line 283 - The supplemental Figure S6 and S7 seem to have regions of heightened recombination, but this is difficult to inter
make these consistent. I would also like to see the syri graph of the two aligned genomes, as this would allow for a visual cor
Figures S6 and S7 have been replaced with improved figures for ease of interpretability. X-axis is now constant across all chr
SyRI plot has been added into the supplementary. Citation for plotter was also added to methods. The following text has been
annotation”, introducing and discussing the SyRI plot.

“The distribution of synteny, inverted, translocated, and duplicated regions between the genomes of E. melliodora and E. side
all chromosomes exhibited a substantial number of rearrangements distributed across their entire length. Notably, chromosor
inversion. These observations highlight the complexity of genome structural evolution and emphasise the need to investigate

Line 290 - How were p-values adjusted?
P-values were adjusted by using Tukey’s test. Text has been modified to make this more clear.

Changed:
“To determine if any region type/s were contributing to differences in ρ, we performed Tukey’s test, adjusting p-values to acc
To:
“To determine if any region type/s were contributing to differences in ρ, we performed Tukey’s test. Tukey’s test adjusted p-v

Line 294 - More information about this 'significantly' higher recombination rate would be good, either in the figure or further e
We have modified this text to make it more biological and less technical, better explaining our results.

Changed:
“Notably, genes followed by transposons exhibited significantly higher ρ than all other types of regions, while duplications sho
To:
“Notably, our results suggest that genes and transposons undergo recombination more frequently than other genomic regions
passed onto offspring may be the most highly diverse among the regions tested. Furthermore, duplications showed higher va

Line 307 - Italics for species names (repeated in Figure 10 and Figure 11 caption)
All instances of species names have now been checked for italics and updated accordingly.

Line 310 - Similar problem to line 275

Title changed from:
"Effect of Synteny, rearranged, unaligned, and genes on Fixation index (FST)"
To:
"Effect of syntenic, rearranged, unaligned regions and genes on Fixation index (FST)"

Figure 10 - Having Figure 9b repeated in Figure 10 and Figure 11 is unnecessary.
We have removed the table from Figs 10 and 11 and altered text to suit. Captions for Figures 10 and 11 now direct reader to

Line 336 - Vertical lines show average FST, not p
We have corrected this to be Fst not ρ.

Line 341 - Similar problem to line 275
Title changed from:
"Effect of Synteny, rearranged, unaligned, and genes on SNPs"
To
"Effect of syntenic, rearranged, unaligned regions and genes on SNPs"

Line 356 - translocations should be plural
Changed:
"Conversely, unaligned and translocated regions had low ρ, while only translocation had few SNPs."
To
"Conversely, unaligned and translocated regions had low ρ, while only translocations had few SNPs."

Line 367 - Vertical lines show average SNP density, not p
Changed:
"Vertical lines show average ρ for syntenic regions."
To
"Vertical lines show average SNP density for syntenic regions."

Line 391 - This is the first mention of barrier loci, please define
A brief definition and citation has been added.
Changed:
"Successful offspring are survivors of genetic combinations, possessing genomes sufficiently free from barrier loci to allow rep
To:
"Successful offspring are survivors of genetic combinations, possessing genomes sufficiently free from barrier loci (genomic lo
allow reproduction to occur."

55. Ravinet M, Faria R, Butlin RK, Galindo J, Bierne N, Rafajlović M, et al.. Interpreting the genomic landscape of speciation:
doi: 10.1111/jeb.13047.

Line 413 - As mentioned above, I would recommend a formal enrichment test to support this statement
Gene ontology (GO) term enrichment test has been added, see response above to "Line 266".

Line 428 - Grammar is poor here, please correct
Changed:
"Fixed unaligned regions are possibly highly diverged regions or a deletion in the opposite species genome. When unaligned r
host species genome or deletions within the opposite species genome."
To:
"Fixed unaligned regions may correspond to highly divergent regions or deletions in the genome of the other species. Polymo
species genome or deletions within the genome of the other species."

Line 490 - Please make this a complete sentence
Changed:
"DpnII, HinFI, MseI, DdeI to digest the genome."
To:
"The restriction enzymes DpnII, HinFI, MseI, and DdeI were used to digest the genome."

Line 499 - Please state how the Hi-C map was manually edited, and what informed the position of those edits.
Extra detail on manual Hi-C editing has been added to methods.

Added text:
"After initial scaffolding the Hi-C contact map was manually edited with Juicebox [78] (version: 2.16). Briefly, the Hi-C contac
scaffolds. For example, scaffolds 2 and 3, and 4 and 5 (Supplementary Figure S1) were manually joined, as indicated by their
heatmap. Additionally, contigs displaying strong off-diagonal signals were reviewed, and if the off-diagonal signal was strong
the off-diagonal signal."

Line 508 - Please provide an example of how well your LAI score of ~18 compares. The LAI paper seems to intimate that 10
Changed
"LAI identifies long terminal repeat (LTR) sequences and reports on the proportion that are intact; more complete genomes h
To
"LAI identifies long terminal repeat (LTR) sequences and reports on the proportion that are intact. Within their publication, Ou
draft genomes, scores of 10-20 indicate reference genomes, and scores of 20 or higher represent gold-quality genomes."

Line 513 - Missing bracket for version number
Fixed as follows:
"Genomes were annotated for transposable elements (TE) using genome-specific, de novo repeat libraries created with EDTA
To:
"Genomes were annotated for transposable elements (TE) using genome-specific, de novo repeat libraries created with EDTA

Line 536 - Syntenic rather than synteny
Changed:
"Using SyRI [85] (version: 1.5), filtered nucmer alignments were analysed and subsequently genomes were annotated for sy
regions."
To:
"Using SyRI [85] (version: 1.5), filtered nucmer alignments were analysed and subsequently genomes were annotated for sy
regions."
Line 717 - Formatting error in references
We have checked the references in the submitted manuscript and updated accordingly, complying with GigaScience's guidelin

Supp table S3-S4-S5 - Space between E. and sideroxylon
Table headings have been fixed.

Reviewer #2:
Structural variation plays an important role in the domestication and adaptability of species. The author compared the structu
populations. This is a very interesting study, but it feels that the author is just statistical data. However, the biological proble
as the impact of structural variation on recombination. What effect does it have on the differentiation of the two populations?
not very clear, and some of the results are described too simply, resulting in unclear conclusions. When formatting pictures, t
them. However, some obvious issues, but not limited, are listed above.

We thank the reviewer for the time and effort taken to review our manuscript and are grateful that they see value in this area
all of reviewer 2s comments below and used their suggestions to improve the quality of our manuscript.

Here are other minor issues:
1. Lines 62-64: References are required.
The following two citations have been added to the introduction. Supporting the claim that "Using third-generation long-read
Technologies and PacBio, evolutionary genomic studies can now affordably assemble highly contiguous genomes of several in

Kovaka S, Ou S, Jenike KM, Schatz MC. Approaching complete genomes, transcriptomes and epi-omes with accurate long-rea
doi: 10.1038/s41592-022-01716-8.

Marx V. Method of the year: long-read sequencing. Nat Methods. Nature Publishing Group; 2023; doi: 10.1038/s41592-022-0

2. Lines 145-150: It is recommended to put it in the materials and methods section.
We agree that this writing was indeed too focused on methodology, however we also want to aid the reader in their understa
and reduced the volume of text, leaving only the basic outline of the analysis performed.

Changing:
Shared sequences between E. melliodora and E. sideroxylon were identified using nucmer from the MUMmer toolset. Subsequ
syntenic, inverted, translocated, or duplicated, and both genomes accordingly annotated for these regions. Additionally, both
unique to each genome, resulting from insertions, deletions, or divergence beyond recognition.
To:
Shared sequences between E. melliodora and E. sideroxylon were identified, classified as syntenic, inverted, translocated, or
Additionally, unaligned regions in each genome, arising from insertions, deletions, or divergence, were annotated.

3. The Synteny and structural variation annotation section requires a detailed explanation of the results in Figure 2 and Table
In response to comment "Line 283" from Reviewer 1 this section now contains additional details on structural variation annot
in understanding our results.

Details of manuscript modifications, copied from response to Reviewer 1:

Figures S6 and S7 have been replaced with improved figures for ease of interpretability. X-axis is now constant across all chr
SyRI plot has been added into the supplementary. Citation for plotter was also added to methods. The following text has bee
annotation", introducing and discussing the SyRI plot.

"The distribution of synteny, inverted, translocated, and duplicated regions between the genomes of E. melliodora and E. side
all chromosomes exhibited a substantial number of rearrangements distributed across their entire length. Notably, chromosor
inversion. These observations highlight the complexity of genome structural evolution and emphasise the need to investigate

4. It is recommended to make Table 2 into a picture, the effect will be better.
Table 2 is intended to be read alongside Figure 2. Combined they provide the reader with the following information for each r
average size, total amount of the genome occupied by the event type. We prefer to leave the table as is, as it provides an ac

5. The form should be a three-line grid.
We are unable to understand what this comment refers to. It could be interpreted as a suggestion on formatting, however ou
guidelines without explicit need for further modification.

6. Why does the recombination rate in Table 3 have positive and negative errors at the genome level, but only negative error
Within this table we present averages and ranges. For recombination rate estimates we also provided the standard deviation,
To reduce confusion and enhance readability, we have omitted the standard deviation, as we already present ranges.

7. 219-220 It is recommended that methods not appear in the results section. It is recommended to put it in the methods sec
The sentence in question reads "Short-read alignments with low mapping scores may confound genotyping of unaligned regio
however as this is an important point that affects how results are analysed and discussed we prefer to leave it in for clarity ar
to support the point made in the sentence. If moved to the methods section, these references may be overlooked by the reac

8. The Structural variation genotyping in the results section needs to be modified.
Without more specific comments from reviewer 2 we are unable to ascertain the concern that has been raised here. However
made from reviewers 1 and 3. Accordingly, we believe this section has been improved overall, with better clarity and interpre
concern.

9. Figure 6 is a bit confusing. It is recommended to revise it to make it clearer.
We agree that this table was confusing, and not the best method to present our results. We have removed Figure 6 and repla
number and percent of events classified as SD, SP, and SSP. Supplementary Table S2 gives details on inversion and transloca
interpretability of these complex findings.

Accordingly, manuscript text has changed from:
"The remaining proportion consisted of SPs (inversions: 25.98%, translocations: 24.80%) or SSPs (inversions: 7.79%, transl
To:

“The remaining proportion consisted of SPs or SSPs, and a small number of SD. For details on inversion and translocation clas
classification, see Supplementary Table S2.”

10. The results section of Figure 7 is not clearly described and the notes are not clear. What do the different colors represent
In response to this comment and also to a comment from reviewer 1, Figure 7 has been updated. Figure 7 now includes a leg

11. Lines 263-264: It is recommended that methods should not appear in the results section, but can be placed in the materi
Due to similar comments from reviewer 1, this text has been modified. The methods have now been removed from the result
section, where it is more appropriately placed.

Accordingly, the original:
“Examination of SSPs identified 281 gene candidates (E. melliodora: 145 and E. sideroxylon: 136), of which 247 (87%; E. me
annotated into eggNOG orthogroups and grouped into COG (Clusters of Orthologous Groups) [39] categories, Figure 8. Simila
COG categories. Comparing all genes to SSP genes indicates that SSP genes have an increased association with DNA replicati
modification, protein turnover, chaperones, signal transduction, intercellular communication, and unexplored aspects of biolo
with categories for fundamental cellular functions, such as protein synthesis, defence against pathogens, maintaining cellular
molecular processes involving amino acids, nucleotides, and coenzymes.”
Has been succinctly changed to:
“After attempting to functionally annotate all genes across the genome and placing them within COG categories [39], 247 of
Figure 8). These genes were enriched for DNA replication, DNA recombination, DNA repair, post-translational modification, pr
communication, and unexplored aspects of biology. SSP genes were found to be underrepresented in categories related to fu
against pathogens, maintaining cellular integrity, providing structural support, and regulating crucial molecular processes inv

12. It is recommended that Figure 8 be divided into Figure 8A and Figure 8B. Try not to have pictures within pictures, which
As the data presented covers the same categories and dividing into 8A and 8B would require enlarging the figure we have lef
readability we have moved the embedded barplot out and made the figure bigger.

13. Lines 276-281: It is recommended to put it in the method section.
We have made this section more brief, leaving only the essential details, and moved the mentioned methodological text into
the readability.

Results changed from:
“After annotating SVs in both species and genotyping their frequencies, we calculated ρ across the reference genomes. As lov
we considered only fixed SVs and excluded events shorter than 2 Kbp, as ρ was calculated within 1 Kbp windows. We also as
on ρ. Prior to ρ calculations, we phased SNPs, initially achieving 20.56% linkage within haplotype blocks using read alignmen
approach.”
To
“After annotating SVs in both species and determining their frequencies, we calculated ρ for fixed SVs longer than 2 Kbp acro
phased SNPs, initially achieving 20.56% linkage within haplotype blocks using read alignments, and then finalised the phasin

Text added to methods:
“As low-frequency SVs are unlikely to have a detectable effect on ρ, we considered only fixed SVs and excluded events short
also assessed the impact of genes and transposons larger than 2 Kbp on ρ. Prior to ρ calculations, we phased SNPs, initially a
alignments, and subsequently completing phasing with a HMM-based approach.”

14. Lines 289-290: It is recommended to put it in the method section.
The text in question (see below) we prefer to leave in place as it gives the reader a better understanding of the logic behind

“To determine if any region type/s were contributing to differences in ρ, we performed Tukey’s test, adjusting p-values to ac

15. Lines 307-308: E. melliodora and E. sideroxylon italics
All instances of species names have been checked for italics and updated accordingly, as suggested by the reviewer.

16. Lines 311-318, lines 320-321: It is recommended to put them in the method section.
Lines 311-318
We agree with the reviewers recommendation and have moved this text to the methods section “Fixation index (FST)”.

320-321:
The text in question (see below) is short (31 words), and serves to aid the reader in quickly understanding our methods. The

“As per our examination of ρ, we calculated the average FST for all fixed SVs, and genes and transposons greater than 2 Kbp

17. Lines 338-339: E. melliodora and E. sideroxylon italics.
All instances of species names have been checked for italics, and updated accordingly, as suggested by the reviewer.

18. Line 342: It is recommended to put it in the discussion.
We believe that retaining this sentence is essential as it provides motivation to the reader regarding the purpose of our analy

“SNP density can significantly impact the precision and resolution of both ρ and FST [70–72].”

19. It is recommended to change Figure 9B, Figure 10B and Figure 11B to Figure
We have removed the table from Figs 10 and 11 and altered text to suit. Captions for Figures 10 and 11 now direct the reade

20. Line 561: Add references.
A citation for BCFtools has been added as recommended.

Reviewer #3:
The manuscript sheds light on the impact of different categories of structural variants (SVs) in two closely related Eucalyptus
improvement. Here are some detailed comments:

1. Please thoroughly revise the formatting of the entire manuscript, including figures and tables, to comply with the journal's
text using brackets at the end of corresponding sentences. Latin names should be italicized in the main text, figures, and tab

We thank reviewer 3 for their time and suggestions, which have helped improve our manuscript for publication. We have che
listed.Our new improved manuscript incorporated the suggestions by the reviewer and is also now in full compliance with Gig
similar problems, which have all been updated, in a highly improved manuscript.

2. In the Introduction, figures and tables are generally not cited. Please adjust the corresponding text accordingly and cite Fi
Figure 1 is from the results of this manuscript or from published literature. If it's from this study, it should be cited at the enc
appropriate citations should be included.

Our introduction contains a single figure (Figure 1), created by us to aid in understanding the purpose of our study. It demon
two species. When unique to a single species, SVs can additionally be fixed or polymorphic.

As this figure was created by us based on the information presented in our introduction, it is not cited. Additionally, the use c
common in many studies. In recognition of the concern by reviewer 3 and to ensure clarity as to the origin of this figure, we
highlights the figure is indeed created by us for illustration purposes based on the literature discussed in the introduction.

Changed:
“An ancestral population, once highly syntenic, undergoes division into two non-interbreeding groups, with structural variatio
To:
“An ancestral population, once highly syntenic, undergoes division into two non-interbreeding groups, with structural variatio
Figure 1. “

3. The B panels in Fig. 9, Fig. 10, and Fig. 11 are all the same. It's redundant to present them three times. Please remove the
Reviewer 2 also raised this concern (comment 19), and we have since improved these figures for clarity. We have removed t
for Figures 10 and 11 now direct the reader to Figure 9 for counts. Overall, redundancy has been removed and succinct, clea

4. Please remove Table 1. The data in this table has already been presented in a published paper ("Interspecies genome dive
rearrangements in Eucalyptus"). Reusing data in this manner is unreasonable.

We agree that the contigs scale data presented within both manuscripts are identical, however scaffolding level data is differe
tools have been improved and their reported results differ (see example of genome repeat content below). Due to these char
Table 1 as is.

Scaffold N50 (bp)
Interspecies genome divergence is predominantly due to frequent small-scale rearrangements in Eucalyptus (Molecular Ecolo
E. melliodora: 60,825,648 bp
E. sideroxylon: 62,127,342 bp

Exploring polymorphic interspecies structural variants in Eucalyptus: Unravelling Their Role in Reproductive Isolation and Ada
E. melliodora: 59.47 Mbp
E. sideroxylon: 60.48 Mbp

Repetitive % (TE %)
Interspecies genome divergence is predominantly due to frequent small-scale rearrangements in Eucalyptus.
E. melliodora: 48.41% (47.20%)
E. sideroxylon: 47.82% (46.57%)

Exploring polymorphic interspecies structural variants in Eucalyptus: Unravelling Their Role in Reproductive Isolation and Ada
E. melliodora: 48.50% (47.13%)
E. sideroxylon: 47.83% (46.58%)

5. Please provide a rationale for selecting these two species for the study in the Introduction section. This background inform
We have added details on study species choice to the “Population sampling and sequencing” section of methods.

From:
“Yellow box (Eucalyptus melliodora) and red ironbark (E. sideroxylon) are closely-related eucalypts of the box-gum grassy wo
often found growing in sympatry or parapatry, and widely hybridise throughout their ranges in southeastern Australia. We co
To
“Yellow box (Eucalyptus melliodora) and red ironbark (E. sideroxylon) are closely-related eucalypts of the box-gum grassy wo
often found growing in sympatry or parapatry, and widely hybridise throughout their ranges in southeastern Australia. Additio
adaptation and introgression studies [70–72], contributing to the availability of large genetic datasets for these species, maki
melliodora and 180 E. sideroxylon, …”

6. Please make all data used in this study available before the manuscript is accepted for publication.
All data and scripts have been made publicly available as requested. Additionally, we have updated the “Data access” section
genomes, analysis data, etc can be publicly found.

Data access
“Sequencing data and reference genomes generated in this project are publicly available on the Sequence Read Archive (SRA
and PRJNA578806. Gene predictions, repeat annotations, SNP vcf, eggNog annotations, PCA data, recombination rate estima
and SyRI output have been deposited in FigShare and are available at:
https://figshare.com/projects/Exploring_polymorphic_interspecies_structural_variants_in_Eucalyptus_Unravelling_Their_Role
. All analysis scripts created and used by this project have been deposited within our github repository: https://github.com/fe
