# Supplementary material for: Exploring the role of polymorphic interspecies structural variants in reproductive isolation and adaptive divergence in Eucalyptus
Source: Gigascience. 2024 Jun 13;13:giae029. doi: 10.1093/gigascience/giae029 (PMC11170218; doi:10.1093/gigascience/giae029)
Supplement: giae029_Reviewer_1_Report_Original_Submission — Jakob Butler -- 12/3/2023 [file giae029_reviewer_1_report_original_submission.pdf]

Reviewer Report

Title: Exploring the role of polymorphic interspecies structural variants in reproductive isolation and adaptive divergence in Eucalyptus

Version: Original Submission **Date:** 12/3/2023

Reviewer name: Jakob Butler

Reviewer Comments to Author:

Ferguson et al have performed a thorough analysis of two species of Eucalyptus, quantifying the extent of structural variation between assembled genomes of the species and determining how prevalent those variations are across a selection of wild material. I believe this study is of sufficient quality for publication in GigaScience, if some minor inconsistencies and grammatical issues are addressed, and a few supporting analyses are performed.

The major changes I would like to see include the addition of a syri plot of the complete set of SVs between E. melliodora and E. sideroxylon. I believe this, along with correcting the scale on the plots of recombination in Figure S6/S7 would allow for a better comparison of how recombination rate is interacting with the SVs. I would also suggest a more formal test of enrichment for COG terms, to better support the statements of "enrichment" in the discussion.

Suggested changes by line:

Line 142 - This section is quite short, I would either merge this section into the Genome scaffolding (and annotation) section, or expand on the results of the gene annotation.

Line 182 - (Supplementary Figure S4)

Line 183 (and throughout) - Please be consistent with your references to tables and figures.

Line 186 - delete comma after 28.63%

Line 194 - These are density plots rather than histograms

Figure 4 - Both axes are labelled as PC1

Line 217 (page 10, line numbers are doubled up) - This seems repetitive, perhaps "…especially as they may also represent divergent sequences".

Line 221 (page 11) - Please insert "and" before polymorphic translocations

Line 223 - You have stated that those not successfully genotyped in both species are private or artefacts earlier in the paragraph, please reduce the repetition.

Figure 6 - I don't find this figure particularly informative (and somewhat confusing to interpret). I think showing the percentages of each different SV in a visual form implies a level of equivalence in genomic impact, which is difficult to reconcile with the raw difference in numbers. I think a supplemental table with the focus on the percentages would illustrate the point better.

Line 246 - There is no mention in the methods about what r threshold was used to declare a pair "correlated", please state it here or in the methods.

Line 265 - This line was confusing to interpret. A suggested alteration: "significant value. After attempting to functionally annotating all genes across the genome and placing them within COG categories, 247 of the total 281 gene candidates in SSPs were annotated. These genes were enriched

for...."
Line 266 - I would like to see a formal enrichment analysis rather than "increased/decreased association", so we could have a clearer picture of which gene functions are truly over/under-represented in SSPs. You could subsequently limit Figure 8 to those that show a difference.
Line 275 - The grammar of this title is a bit off, perhaps "Effect of syntenic, rearranged, unaligned regions and genes on recombination rates"
Line 276 - This is the first mention of p, please define it as recombination rate
Line 283 - The supplemental Figure S6 and S7 seem to have regions of heightened recombination, but this is difficult to interpret and compare with the current variable axis scales. Please make these consistent. I would also like to see the syri graph of the two aligned genomes, as this would allow for a visual comparison of SV regions with recombination rate.
Line 290 - How were p-values adjusted?
Line 294 - More information about this 'significantly' higher recombination rate would be good, either in the figure or further expanded in the text.
Line 307 - Italics for species names (repeated in Figure 10 and Figure 11 caption)
Line 310 - Similar problem to line 275
Figure 10 - Having Figure 9b repeated in Figure 10 and Figure 11 is unnecessary.
Line 336 - Vertical lines show average FST, not p
Line 341 - Similar problem to line 275
Line 356 - translocations should be plural
Line 367 - Vertical lines show average SNP density, not p
Line 391 - This is the first mention of barrier loci, please define
Line 413 - As mentioned above, I would recommend a formal enrichment test to support this statement
Line 428 - Grammar is poor here, please correct
Line 490 - Please make this a complete sentence
Line 499 - Please state how the Hi-C map was manually edited, and what informed the position of those edits.
Line 508 - Please provide an example of how well your LAI score of ~18 compares. The LAI paper seems to intimate that 10 is low quality?
Line 513 - Missing bracket for version number
Line 536 - Syntenic rather than synteny
Line 717 - Formatting error in references
Supp table S3-S4-S5 - Space between E. and sideroxylon

Methods

Are the methods appropriate to the aims of the study, are they well described, and are necessary controls included? Choose an item.

Conclusions

Are the conclusions adequately supported by the data shown? Choose an item.

Reporting Standards

Does the manuscript adhere to the journal's guidelines on minimum standards of reporting? Choose an item.

Choose an item.

Statistics

Are you able to assess all statistics in the manuscript, including the appropriateness of statistical tests used? Choose an item.

Quality of Written English

Please indicate the quality of language in the manuscript: Choose an item.

Declaration of Competing Interests

Please complete a declaration of competing interests, considering the following questions:

- Have you in the past five years received reimbursements, fees, funding, or salary from an organisation that may in any way gain or lose financially from the publication of this manuscript, either now or in the future?
- Do you hold any stocks or shares in an organisation that may in any way gain or lose financially from the publication of this manuscript, either now or in the future?
- Do you hold or are you currently applying for any patents relating to the content of the manuscript?
- Have you received reimbursements, fees, funding, or salary from an organization that holds or has applied for patents relating to the content of the manuscript?
- Do you have any other financial competing interests?
- Do you have any non-financial competing interests in relation to this paper?

If you can answer no to all of the above, write 'I declare that I have no competing interests' below. If your reply is yes to any, please give details below.

I declare that I have no competing interests

I agree to the open peer review policy of the journal. I understand that my name will be included on my report to the authors and, if the manuscript is accepted for publication, my named report including any attachments I upload will be posted on the website along with the authors' responses. I agree for my report to be made available under an Open Access Creative Commons CC-BY license (http://creativecommons.org/licenses/by/4.0/). I understand that any comments which I do not wish to be included in my named report can be included as confidential comments to the editors, which will not be published.

Choose an item.

To further support our reviewers, we have joined with Publons, where you can gain additional credit to further highlight your hard work (see: https://publons.com/journal/530/gigascience). On publication of this paper, your review will be automatically added to Publons, you can then choose whether or not to claim your Publons credit. I understand this statement.

Yes Choose an item.
