# Supplementary material for: Exploring the role of polymorphic interspecies structural variants in reproductive isolation and adaptive divergence in Eucalyptus
Source: Gigascience. 2024 Jun 13;13:giae029. doi: 10.1093/gigascience/giae029 (PMC11170218; doi:10.1093/gigascience/giae029)
Supplement: giae029_Reviewer_2_Report_Original_Submission — Lejun Ouyang -- 1/27/2024 [file giae029_reviewer_2_report_original_submission.pdf]

Reviewer Report

Title: Exploring the role of polymorphic interspecies structural variants in reproductive isolation and adaptive divergence in Eucalyptus

Version: Original Submission **Date:** 1/27/2024

Reviewer name: Lejun Ouyang

Reviewer Comments to Author:

Structural variation plays an important role in the domestication and adaptability of species. The author compared the structural variation between E. melliodora and E. sideroxylon populations. This is a very interesting study, but it feels that the author is just statistical data. However, the biological problems caused by these differences have not been condensed, such as the impact of structural variation on recombination. What effect does it have on the differentiation of the two populations? Is it promoting or inhibiting? Secondly, the author's writing is not very clear, and some of the results are described too simply, resulting in unclear conclusions. When formatting pictures, try to avoid nesting pictures, and use A, B, C, etc. to represent them. However, some obvious issues, but not limited, are listed above.
Here are other minor issues:

1. Lines 62-64: References are required.
2. Lines 145-150: It is recommended to put it in the materials and methods section.
3. The Synteny and structural variation annotation section requires a detailed explanation of the results in Figure 2 and Table 2.
4. It is recommended to make Table 2 into a picture, the effect will be better.
5. The form should be a three-line grid.
6. Why does the recombination rate in Table 3 have positive and negative errors at the genome level, but only negative errors at the chromosome average level?
7. 219-220 It is recommended that methods not appear in the results section. It is recommended to put it in the methods section.
8. The Structural variation genotyping in the results section needs to be modified.
9. Figure 6 is a bit confusing. It is recommended to revise it to make it clearer.
10. The results section of Figure 7 is not clearly described and the notes are not clear. What do the different colors represent?
11. Lines 263-264: It is recommended that methods should not appear in the results section, but can be placed in the materials and methods section.
12. It is recommended that Figure 8 be divided into Figure 8A and Figure 8B. Try not to have pictures within pictures, which can easily lead to unclear references.
13. Lines 276-281: It is recommended to put it in the method section.
14. Lines 289-290: It is recommended to put it in the method section.
15. Lines 307-308: E. melliodora and E. sideroxylon italics
16. Lines 311-318, lines 320-321: It is recommended to put them in the method section.
17. Lines 338-339: E. melliodora and E. sideroxylon italics.

18. Line 342: It is recommended to put it in the discussion.
19. It is recommended to change Figure 9B, Figure 10B and Figure 11B to Figure
20. Line 561: Add references.

Methods

Are the methods appropriate to the aims of the study, are they well described, and are necessary controls included? Choose an item.

Conclusions

Are the conclusions adequately supported by the data shown? Choose an item.

Reporting Standards

Does the manuscript adhere to the journal's guidelines on minimum standards of reporting? Choose an item.

Choose an item.

Statistics

Are you able to assess all statistics in the manuscript, including the appropriateness of statistical tests used? Choose an item.

Quality of Written English

Please indicate the quality of language in the manuscript: Choose an item.

Declaration of Competing Interests

Please complete a declaration of competing interests, considering the following questions:

- Have you in the past five years received reimbursements, fees, funding, or salary from an organisation that may in any way gain or lose financially from the publication of this manuscript, either now or in the future?
- Do you hold any stocks or shares in an organisation that may in any way gain or lose financially from the publication of this manuscript, either now or in the future?
- Do you hold or are you currently applying for any patents relating to the content of the manuscript?
- Have you received reimbursements, fees, funding, or salary from an organization that holds or has applied for patents relating to the content of the manuscript?
- Do you have any other financial competing interests?
- Do you have any non-financial competing interests in relation to this paper?

If you can answer no to all of the above, write 'I declare that I have no competing interests' below. If your reply is yes to any, please give details below.

I declare that I have no competing interests

I agree to the open peer review policy of the journal. I understand that my name will be included on my report to the authors and, if the manuscript is accepted for publication, my named report including any attachments I upload will be posted on the website along with the authors' responses. I agree for my report to be made available under an Open Access Creative Commons CC-BY license (http://creativecommons.org/licenses/by/4.0/). I understand that any comments which I do not wish to be included in my named report can be included as confidential comments to the editors, which will not be published.

Choose an item.

To further support our reviewers, we have joined with Publons, where you can gain additional credit to further highlight your hard work (see: https://publons.com/journal/530/gigascience). On publication of this paper, your review will be automatically added to Publons, you can then choose whether or not to claim your Publons credit. I understand this statement.

Yes Choose an item.
